# *Wolbachia* uses ankyrin repeats to target specific fly proteins

**DOI:** 10.1128/mbio.00172-26

**Published:** 2026-04-20

**Authors:** William Hamilton, Erin Hardy, Sergio López-Madrigal, Melissa Phelps, MaryAnn Martin, Irene Newton

**Affiliations:** 1 Department of Biology, Indiana University, Bloomington, Indiana, USA; Max Planck Institute for Chemical Ecology, Jena, Germany

**Keywords:** *Wolbachia*, ankyrin, *Drosophila*, effector

## Abstract

Arthropods, the most diverse phylum on Earth, are hosts to a plethora of bacterial parasites that secrete various effectors of unknown function during infection. The most prevalent of these is the intracellular bacterium *Wolbachia pipientis*. The microbe infects between 40% and 60% of insect species, where it induces a variety of fitness effects ranging from nutritional supplementation to reproductive manipulations and, in some hosts, limiting virus replication. Understanding the molecular basis of *Wolbachia* infection and *Wolbachia-*induced phenotypes is critical to the use of *Wolbachia* in vector control. *Wolbachia* ankyrin repeat proteins (WARPs) represent a highly dynamic and diverse part of the *Wolbachia* pangenome and remain thus far largely uncharacterized. Here, we perform molecular and genetic screens to identify interactions between *Wolbachia w*Mel WARPs and their target host proteins in *Drosophila melanogaster*. Our results identify strong interactions of two *Wolbachia* proteins, WARP434 and WARP754, with multiple host targets. Heterologous expression of these two WARPs is extremely toxic in *Drosophila* tissues, and the toxicity is dependent on the ankyrin repeat domain of each WARP. We use coimmunoprecipitation (coIP) and mass spectrometry to identify native targets of the WARPs, and importantly, knockdown of host targets alleviates toxicity, confirming WARP/target interactions. Antibodies targeting both WARPs show expression by *Wolbachia* during infection of *Drosophila* cells, and expression of WARP754 in adult flies increases *Wolbachia* titer. Understanding how *Wolbachia* manipulates its host biology and which host pathways it targets during infection will help us define how the most prevalent intracellular bacterial parasite on Earth interacts with its insect hosts at the molecular level. Our screen is an important step toward that goal.

**IMPORTANCE:**

Molecular interactions drive co-evolutionary arms races between hosts and pathogens. These interactions shape the structure and function of both host and parasite proteins, enabling immunity or virulence during infection. Understanding the molecular details that unfold during these events illustrates not only how hosts and parasites co-evolve at the molecular level but also may help characterize the function of poorly understood proteins. The most prevalent intracellular infection on earth is *Wolbachia pipientis*, with between 40% and 60% of insects harboring the bacterial symbiont. Understanding how Wolbachia infects host cells and the molecular tools it uses to alter cell biology is critical to the use of the microbe in vector control. Here, we identify *Wolbachia* proteins used by the symbiont to interface with specific host proteins. Understanding the molecular mechanisms underlying this host–microbe interaction will shed light on how an important symbiont, used in the control of vector populations and disease transmission, uses *Wolbachia* ankyrin repeat proteins (WARPs) to interact with host targets and how targeting this host protein contributes to infection.

## INTRODUCTION

*Wolbachia pipientis* is an obligate intracellular microbe and arguably the most successful infection on our planet, colonizing 40%–60% of insect species (1, 2). *Wolbachia* are alpha-proteobacteria, part of the intracellular *Anaplasmataceae,* and related to the important human pathogens *Anaplasma, Rickettsia,* and *Ehrlichia* (3). However, *Wolbachia* do not infect mammals, but instead are well known for their variety of fitness impacts on hosts which range from nutritional supplementation (4) to reproductive manipulations of insect populations (5). In the last decade, *Wolbachia* have also been shown to provide a benefit to insects, where the infection can inhibit RNA virus replication within the host (6), a phenomenon known as pathogen blocking. Because insects are vectors for disease, and *Wolbachia* alter the ability of these vectors to harbor important human pathogens, *Wolbachia* are being used to control the spread of arthropod-borne diseases, such as dengue.

The establishment of the *Wolbachia* infection itself (i.e., host cell invasion, persistence, proliferation, and transmission to the next generation) is a prerequisite for all of the interesting phenotypes induced by the symbiont. This leads us to ask: *how does Wolbachia manipulate host cell biology to establish an infection?* Like all intracellular bacteria, *Wolbachia* need to alter the host cell to invade and persist. Many microbes accomplish this via secretion systems, nanomachines that enable the microbes to directly transfer proteins from the bacterium into the cytosol of host cells. Virtually all *Wolbachia* strains sequenced to date encode a functional type IV secretion system (T4SS) (7–9), which is expressed by *Wolbachia* within its native host (8, 10). In *Wolbachia*, some predicted secreted effectors are co-regulated with the T4SS, and for a handful of these proteins, heterologous T4SS assays support their secretion by the symbiont (11, 12). Identifying the specific proteins *secreted* by *Wolbachia* has been a primary goal of the field. These proteins, referred to as effectors, often act to manipulate or usurp host cell processes to promote bacterial infection (13, 14). These modes include (but are not limited to) attacking the host cell surface to form pores, inactivating host cytosol machinery to collapse the cytoskeleton, or entering the nucleus to manipulate host gene regulation (15). At each stage of attack, the bacterial effectors often interact directly and specifically with host proteins to perturb a biological process that enables pathogen entry into or defense from the host cell (16–22). Understanding how bacterial effectors function, therefore, has taught scientists not only how pathogens cause disease but also how fundamental cell biological mechanisms work in healthy tissue (23). While effectors are encoded in bacteria, they act within eukaryotic cells and hence feature domains that share structural, functional, and sequence similarity with eukaryotic proteins (13, 14, 24, 25).

Ankyrin repeats (ANKs) are one of the most common domains found in eukaryotic proteins. They were first discovered as repeat sequences in yeast (*Saccharomyces cerevisiae*) proteins Swi6 and Cdc10 and the *Drosophila melanogaster* protein Notch (26). They are characterized by a repeating 33 amino acid motif (containing the conserved N-terminal residues G-X-TPLHLA) that folds into a helix–loop–helix–β-hairpin/loop structure (27). It was previously thought that ANKs are primarily restricted to eukaryotic proteins, but evidence suggests they are also prevalent among bacteria and virus genomes, especially in those microbes that interact with eukaryotes (28, 29). Ankyrin repeat domains often mediate protein–protein interactions and act as a scaffold for protein recognition (30). With the sequencing of the first *Wolbachia* genome from strain *w*Mel, which naturally infects *Drosophila melanogaster,* researchers noted the prevalence of ankyrin repeat domains among encoded proteins (7). Subsequent sequencing of more strains from the *Wolbachia* genus revealed that these intracellular microbes dedicate upward of 4% of their genome to ankyrin repeat-containing proteins, a much higher proportion than in other bacterial genera (29, 31). Interestingly, the *Wolbachia* ankyrin repeat proteins across the *Wolbachia* genus are also highly dynamic. Thus, they are known to change size through expansion and contraction, or domain swapping and addition, and are likely horizontally transferred between strains with the aid of bacteriophages (32). Many in the field, therefore, hypothesize that *Wolbachia’s* ankyrin repeat proteins (WARPs) are involved in manipulating host biology and are likely secreted via one of two secretion systems found in virtually all sequenced *Wolbachia* genomes (T1SS or T4SS); indeed, closely related microbes use ankyrin repeat-containing proteins as secreted effectors (28, 33–37). For example, the pathogen *Anaplasma phagocytophilum* secretes at least three ankyrin repeat-containing proteins (e.g., AnkABC) through its T4SS (38), and AnkA is known to localize to the nucleus, modifying host gene expression (39–41).

Here, we present our study of the 25 WARPs found in strain *w*Mel from *Drosophila*. We show first, through bioinformatics predictive software, that these WARPs are likely secreted. We then show that two WARPs cause severe phenotypes in the fly when overexpressed and that these phenotypes are dependent on the presence of the ankyrin domain. A yeast-2-hybrid (Y2H) screen of the *Drosophila* orfeome and a coimmunoprecipitation (coIP) of each of the native WARPs reveal the host targets to which these toxic WARPs bind, and suppression assays in the fly confirm this interaction. We show that these WARPs are made by *Wolbachia* during an infection, and immunohistochemistry suggests they are secreted outside of the bacterial cell. Finally, we were able to identify an increase in *Wolbachia* titer upon heterologous overexpression of WARP754, suggesting a fitness benefit to the microbe. Understanding the molecular mechanisms underlying this host–microbe interaction will shed light on how an important symbiont, used in the control of vector populations and disease transmission, uses WARPs to interact with host targets and how targeting these host proteins contributes to infection.

## RESULTS

### WARPs are predicted secreted effectors

**Fig 1:**
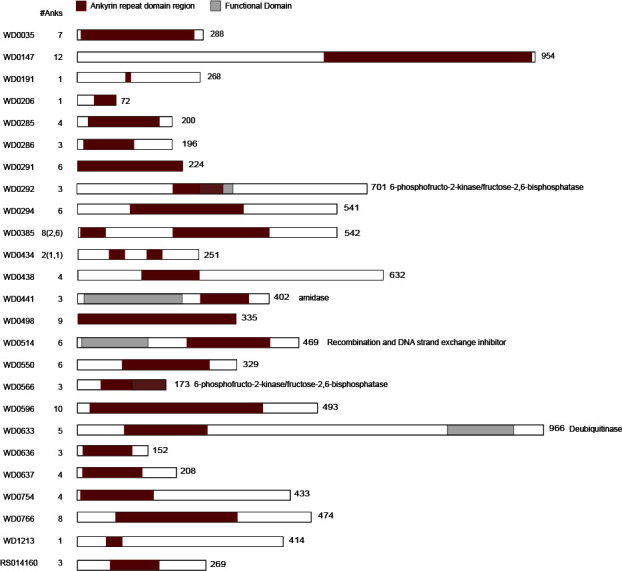
*Wolbachia* WARPs vary in length, number of ankyrin repeats, and functional domain content. For each WARP, the full protein length (right) and number of ankyrin repeats (left) identified by AnkPred software (42) are indicated. Ankyrin repeat boundaries are delineated in red. Predicted functional domains, shown in gray, were identified using HHPred searching the PFAM database (43).

The *Wolbachia* strain *w*Mel, a native symbiont of *Drosophila*, contains 25 WARPs (7), which vary in length from 72 to 966 amino acids and from 1 to 12 ankyrin repeats (Fig. 1). There is also significant variation in predicted functional domains associated with these WARPs, with many of the proteins having no homology to any putative domain and others harboring putative enzymatic domains with predicted functions (such as amidases or glycolysis-associated enzymes). One of the WARPs, WD0633, encodes a deubiquitinase with homology to the OTU protein of *Drosophila melanogaster*.

For *Wolbachia*-predicted effectors to act on host cell biology, they must exit the bacterial cell via some mechanism. *Wolbachia* harbor both a T1SS and a T4SS, and therefore, we sought to use bioinformatic prediction software to determine if any of the *w*Mel WARPs encode signatures of secretion. All 25 WARPs from the *w*Mel genome were used as input to the BastionX v.1.0 software suite (44) (fast mode, all functional analyses checked) (Table 1). BastionX presents model-predicted probabilities from supervised binary classifiers. The prediction module will provide a prediction score and E-value for each secretion system and the most highly scoring mechanism. A little more than half (16/25) of the WARPs were predicted to be secreted via the T4SS. By contrast, only 2/25 WARPs were predicted to be secreted by the T1SS. Few *Wolbachia* proteins have been tested for secretion using a heterologous assay in *Escherichia coli* (12), and only a handful of secreted effectors have been visualized outside of the bacterial cell (i.e., WalE1, the Cif proteins [45, 46]). This computational result, therefore, encouraged us to explore WARP-induced phenotypes in *Drosophila*.

**TABLE 1:** WARP Protein secretion predictions based on BastionX^*a*^^,^^*b*^

	Prediction results based on single-type predictors					Prediction results total	
Accession	Type I	Type II	Type III	Type IV	Type VI	Substrate	Possible secretion system
>Query_WD_0035	0.215	0.195	0.475	0.663	0.275	Yes	IV
>Query_WD_0147	0.088	0.101	0.095	0.607	0.202	Yes	IV
>Query_WD_0191	0.105	0.138	0.154	0.405	0.053	No	–^*c*^
>Query_WD_0206	0.054	0.147	0.337	0.328	0.126	No	–
>Query_WD_0285	0.057	0.042	0.284	0.455	0.109	No	–
>Query_WD_0286	0.029	0.038	0.096	0.345	0.099	No	–
>Query_WD_0291	0.542	0.289	0.62	0.655	0.613	Yes	I III IV VI
>Query_WD_0292	0.205	0.298	0.765	0.904	0.534	Yes	III IV VI
>Query_WD_0294	0.17	0.137	0.187	0.449	0.225	No	–
>Query_WD_0385	0.71	0.14	0.806	0.706	0.443	Yes	I III IV
>Query_WD_0434	0.084	0.217	0.396	0.557	0.085	Yes	IV
>Query_WD_0438	0.074	0.127	0.627	0.953	0.363	Yes	III IV
>Query_WD_0441	0.141	0.101	0.522	0.911	0.25	Yes	III IV
>Query_WD_0498	0.102	0.197	0.562	0.771	0.197	Yes	III IV
>Query_WD_0514	0.164	0.07	0.273	0.662	0.175	Yes	IV
>Query_WD_0550	0.179	0.113	0.126	0.231	0.145	No	–
>Query_WD_0566	0.042	0.112	0.219	0.554	0.108	Yes	IV
>Query_WD_0596	0.081	0.063	0.144	0.342	0.027	No	–
>Query_WD_0633	0.283	0.185	0.838	0.936	0.502	Yes	III IV VI
>Query_WD_0636	0.11	0.088	0.214	0.563	0.115	Yes	IV
>Query_WD_0637	0.08	0.112	0.392	0.681	0.144	Yes	IV
>Query_WD_0754	0.07	0.075	0.619	0.835	0.219	Yes	III IV
>Query_WD_0766	0.162	0.088	0.291	0.495	0.169	No	–
>Query_WD_1213	0.405	0.479	0.871	0.913	0.726	Yes	III IV VI
>Query_WD_RS04160	0.052	0.114	0.414	0.5	0.087	No	–

^*a*^ Putatively secreted *Wolbachia* ankyrin repeat-containing proteins listed by accession and BastionX prediction results highlighted.

^*b*^ The *Wolbachia pipientis w*Mel assembly can be found using NCBI accession AE017196.

^*c*^ The dash is meant to signify that there are no identified secretion systems for that ankyrin repeat protein.

### WARPs cause toxic phenotypes in *Drosophila melanogaster*

**Fig 2:**
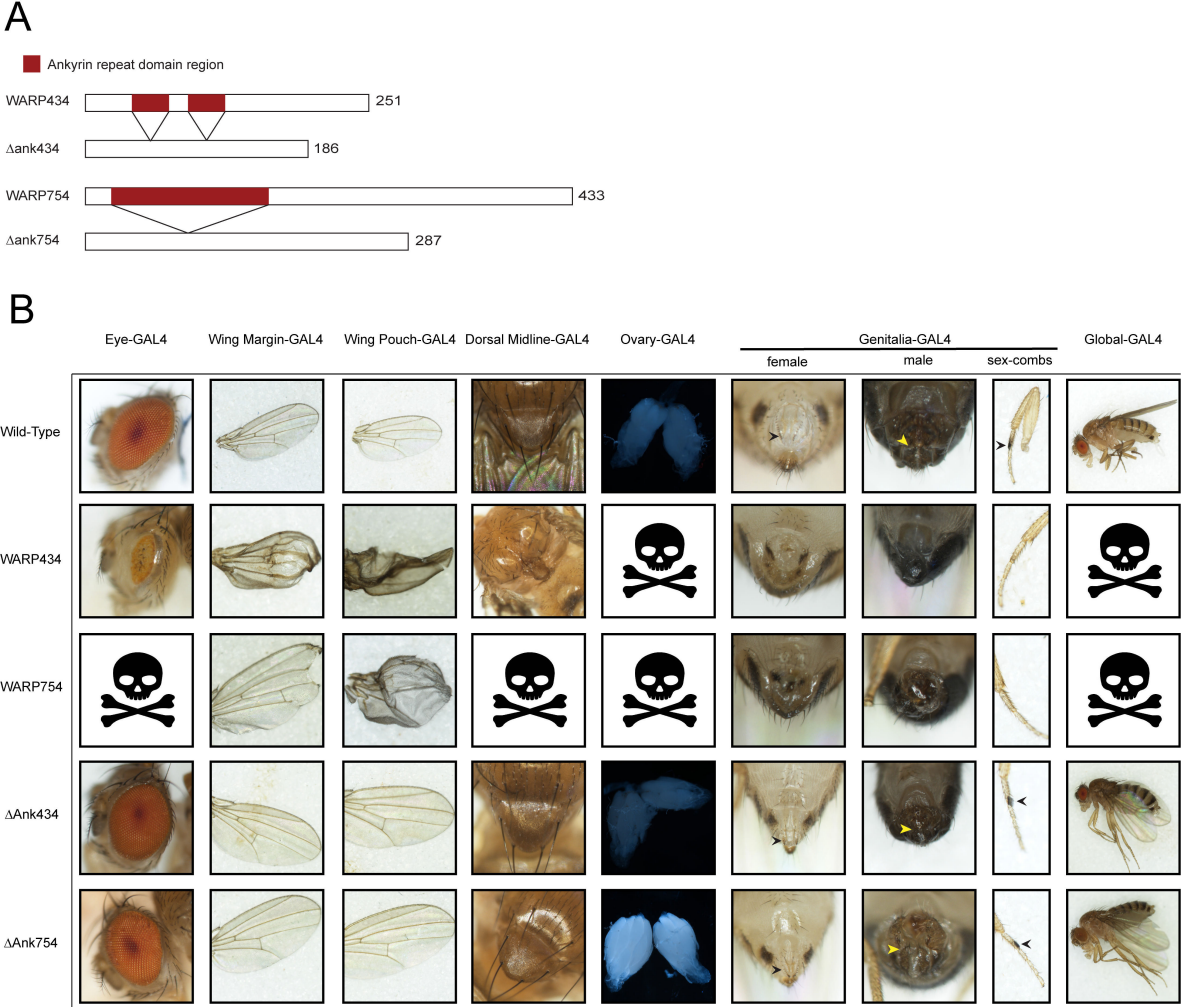
WARP434 and WARP754 induce toxic phenotypes in the fly upon expression. (**A**) Schematic of constructs generated for expression in the fly, both full-length WARPs and those missing the ankyrin repeat domains. (**B**) Wild-type tissues (across top) compared to expression of constructs suggests that the ankyrin repeat domain-containing regions of WARPs are required for toxicity. Arrowheads point to sex combs and genitalia in those relevant panels.

We reasoned that the WARPs, if interfacing with *Drosophila* biology, might induce phenotypes upon overexpression. We built or acquired UAS expression lines for all *w*Mel WARPs (see Materials and Methods). All WARP sequences were codon optimized for *Drosophila* and inserted into attP40 (chromosome II) or attP2 (chromosome III). Flies were homozygosed and crossed to GAL4 expression lines to determine toxicity upon induction. Global expression was compared to expression in the wing pouch and margin, the genitalia and sex combs, the dorsal midline, the eye, and the ovaries. Nearly all WARPs showed no evident phenotype at all upon expression (Fig. S1), although we caution any overinterpretation of this result, which could be related to timing and extent of protein expression. Importantly, expression of the well-characterized *Wolbachia* effector WalE1 also shows no gross phenotype in *Drosophila* (9); lack of phenotype should not be interpreted as lack of function in the symbiosis. Conversely, a severe phenotype upon overexpression should not be interpreted as the natural condition for the fly. We use this model to identify genetic interactions between our WARPs and fly proteins. In our screen, two WARP constructs consistently produced highly toxic phenotypes upon expression in all tested drivers: WARP434 and WARP754 (Fig. 2). Expression of WARP434 leads to death prior to eclosion when expressed systemically. In contrast, expression in the eye led to a glassy-eye phenotype. When expressed in the wings (either margin or pouch), we observed blistering/crumpling of the adult wing that occurs from improper wing vein development. Finally, WARP434 expression caused ablation of both female and male genitalia and ablation of the male sex combs. Like WARP434, WARP754 also caused death when expressed globally, in the ovaries, or in the dorsal midline. WARP754 also induced blistering/crumpling wings when expressed in the wing pouch and ablation of the genitalia. However, unlike WARP434, WARP754 also caused death when expressed in the eye, and the phenotype observed upon expression in the wing margin was clipping on the distal edges of the wing. All the listed phenotypes but the wing pouch were highly prevalent, occurring in all or nearly all observed flies (Table 2).

**TABLE 2:** Ankyrin domains of WARPs mediate toxicity in flies upon expression^*a*^

	Eye	Wing margin	Wing pouch	Dorsal midline	Ovary	Genitalia	Global
WARP434							
Male (phenotype/total)	102/102	107/109	37/173	0/DEAD	0/DEAD	108/116	0/DEAD
Female (phenotype/total)	135/135	122/127	48/180	4/DEAD	0/DEAD	104/113	0/DEAD
?Ank434							
Male (phenotype/total)	0/109	0/115	0/102	0/107	0/144	0/110	0/109
Female (phenotype/total)	0/102	0/110	0/111	0/116	0/131	0/117	0/123
WARP754							
Male (phenotype/total)	0/DEAD	132/132	17/102	0/DEAD	0/DEAD	104/107	0/DEAD
Female (phenotype/total)	0/DEAD	119/119	99/104	0/DEAD	0/DEAD	91/100	0/DEAD
?Ank754							
Male (phenotype/total)	0/100	0/103	0/106	0/101	0/112	0/107	0/148
Female (phenotype/total)	0/114	0/128	0/113	0/119	0/127	0/105	0/133

^*a*^ Counts of flies exhibiting toxic phenotypes (Fig. 2) are shown over the total. In some cases, no transgenic flies eclosed due to WARP-induced lethality (demarcated as DEAD).

Ankyrin repeat domains mediate protein–protein interactions and, for bacterial effectors, are critical to bacterial targeting of specific host proteins (28, 47, 48). Without the ankyrin repeat domain, the bacterial effector no longer properly localizes (28, 48). Therefore, we hypothesized that the ankyrin domains of WARP434 and WARP754 might mediate the toxicity observed upon expression in *Drosophila*. We next generated constructs for both WARPs that lack the ankyrin repeat regions (Fig. 2A).

Interestingly, upon expression of the proteins lacking the ankyrin domains, we no longer observed toxicity, suggesting that the ankyrin repeat domain is necessary for targeting of specific host proteins (Fig. 2B). Yet, it is possible that truncation of the protein abrogated proper folding. We therefore went on to directly test whether the ankyrin repeat domains of WARPs 434 and 754 directly bind *Drosophila* proteins.

### WARPs bind to specific host targets

**Fig 3:**
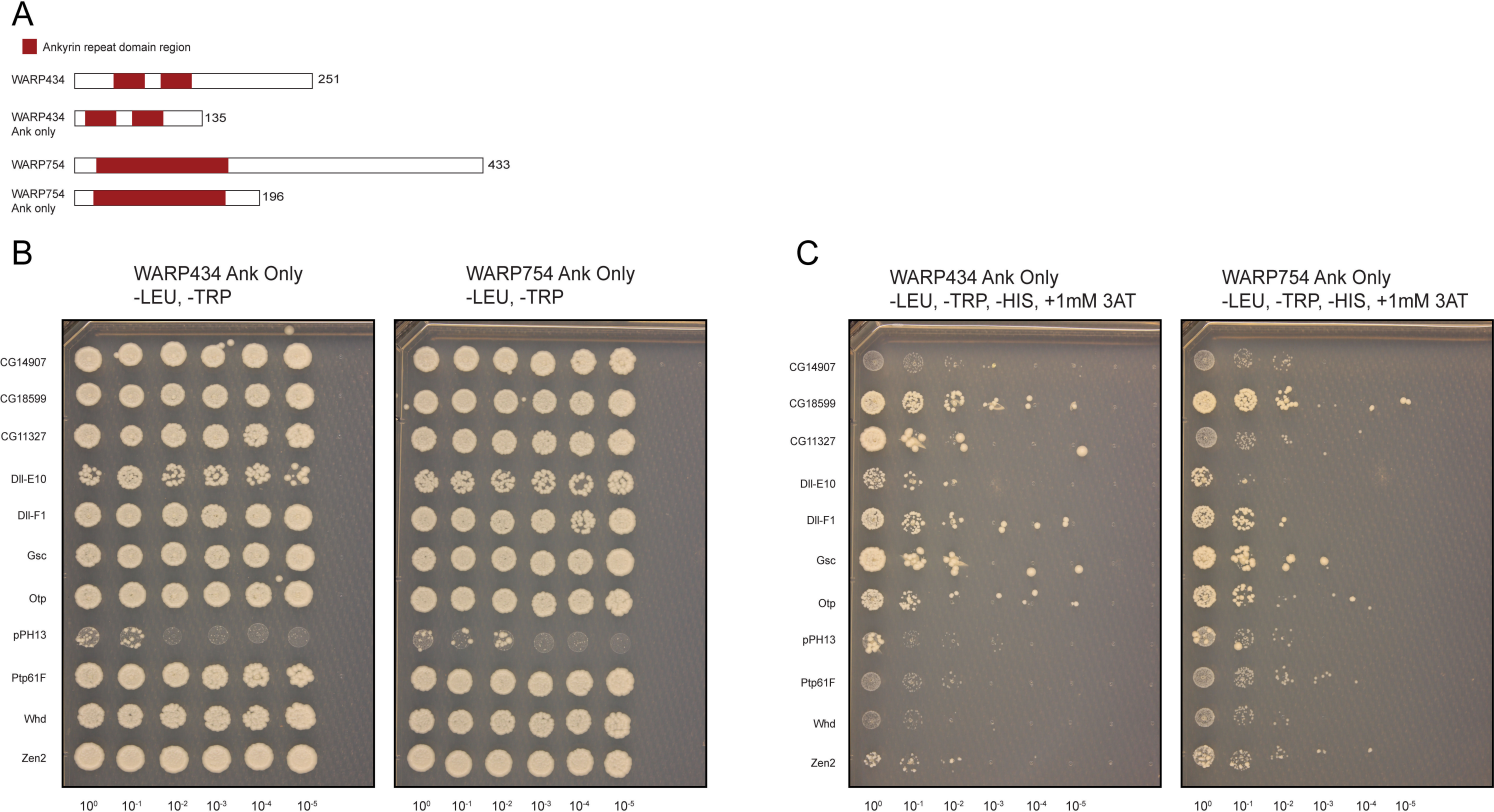
The ankyrin repeat domains of 434 and 754 interact with *Drosophila* proteins in a Y2H assay. (**A**) Schematic of constructs generated for expression in yeast. The N-terminal portions of the proteins containing the ankyrin repeat domains were included in the screen. (**B**) Yeast hybrids grown under permissive conditions were (**C**) replica spotted onto media lacking histidine and containing the drug 3AT at a 1 mM concentration to screen for interactions. WARP434 interacts with CG11327, and WARP754 interacts with Ptp61f.

Our hypothesis was that the ankyrin repeat domains of WARPs mediate interactions with specific *Drosophila* proteins, thereby inducing the phenotypes observed in the whole animal upon overexpression. One way in which to test direct binding is a yeast-2-hybrid assay (49). This assay relies on the reconstitution of a transcription factor in the yeast to drive expression of a metabolic marker gene, allowing for growth in hybrids that harbor interacting proteins. We generated truncated constructs to include primarily the ankyrin repeat domains of WARP434 and 754 (Fig. 3A). These were fused to the DNA binding domain of GAL4 using the pDest-DB-cen plasmid and transformed into *S. cerevisiae* (Y8930). A pool containing both Y8930 DB-ankyrin strains for WARP434 and WARP754 was screened for autoactivation by mating to a pDest-AD-empty Y8800 strain. After 96 h at 30°C, we found no evidence of growth on Y2H selective media containing 1 mM 3AT (3-amino-1,2,4-triazole; Sigma-Aldrich A8056), indicating no autoactivation (49). These WARP-expressing yeasts were then mated to the *S. cerevisiae* activation domain (AD) library containing the Drosophila ORFeome, a collection of 10,490 AD-*Drosophila* ORF fusion strains representing ~2/3 of the *D. melanogaster* proteome. Each stock was mated with the 10,168 viable Y8800 AD-*Drosophila* ORF strains (322 strains did not grow from the glycerol stock; Table S1), plated on Y2H selective media containing 1 mM 3AT, and grown at 30°C for 72–96 h.

The Y2H assay splits a transcription factor (typically GAL4 or LexA) into the DNA-binding domain (BD) and the activation domain (AD). If the WARP protein, fused to the BD, interacts with a *Drosophila* protein fused to the AD domain, the BD and AD are brought together, reconstituting a functional transcription factor. This drives expression of the reporter gene (HIS3), allowing growth on selective media, in this case, media without histidine. We also used 3AT, a competitive inhibitor of the HIS3 gene product, imidazoleglycerol-phosphate dehydratase, which is involved in histidine biosynthesis. In the presence of 3AT, and when HIS3 is used as the reporter gene, yeast cells can grow on media lacking histidine only if HIS3 is sufficiently expressed. Using this methodology, we identified putative host target proteins for both WARPs 434 and 754 (Fig. 3C). Several of our candidate interactors turned out to be auto-inducers (CG18599, DII, Gsc, Otp, pPH13, Zen2; Fig. S2), leaving only two potential host targets: WARP434 interacts with the *Drosophila* protein CG11327 while WARP754 interacts with Ptp61F (Fig. 3C).

In addition to our orfeome screen using the Y2H assay, we also sought to identify native interactions between Wolbachia WARPs and host proteins using coIP. Toward that end, we generated polyclonal antibodies against full-length WARP434 and WARP754 and used the resulting sera, and pre-immune bleeds as controls, for a coIP using Drosophila JW18 cells infected with *Wolbachia w*Mel. Importantly, we do not know when *Wolbachia* may express each WARP during host development, nor in which tissues it is most relevant, but this first approach allowed us to generate enough starting material (*Wolbachia*) to be confident that our coIP was pulling down the native WARPs. For both WARPs, we were able to pull down the target WARP in bulk lysate for the experimental conditions and identified top host protein targets present across all replicates and either not present in our negative controls or significantly diminished in coverage (prebleed controls, Table S2). Interestingly, WARP754 antibody was able to pull down WARP434 by coIP, suggesting a possible interaction between these two proteins. That said, the WARP434 antibody did not reciprocally pull down WARP754, so this result should be cautiously interpreted. In sum, a total of 26 candidate host targets were generated by the coIP, none of which were identified by the Y2H approach.

The Y2H and coIP initial screen suggested interactions between our most toxic WARP proteins and *Drosophila* proteins. However, each of these approaches has pitfalls: for the Y2H, these interactions could be an artifact of the yeast expression system; for the coIP, results could be an artifact of lysate biochemistry. We therefore sought to confirm the involvement of each host protein in the phenotypes induced by the WARPs using *Drosophila* genetics. We established stable lines expressing WARPs in the eye (434; LongGMR-GAL4) or wing (754; AP-GAL4) tissue. Constitutive expression of the WARPs in these tissues leads to severe fly phenotypes (Fig. 2B). We reasoned that if host proteins were directly interacting with WARPs 434 and 754, we should observe a genetic interaction upon knockdown of these loci using RNAi. We acquired flies from the TRiP project harboring a UAS construct driving expression of a short hairpin targeting all host targets available in the BDSC (see Table S3 for fly stocks). We then crossed these TRiP flies to our lines that constitutively express the WARPs in the eye or wing to induce knockdown of the host targets, and the resulting progeny were double-blind scored for the severity of toxicity induced by accompanying WARP expression. A TRiP line targeting GFP was crossed to both WARP434 and WARP754 to verify that any change in phenotype does not occur due to the activation of the RNAi pathway. Additionally, CG11327 and Ptp61F were knocked down in the whole bodies of flies without the WARPs to verify both that no phenotype was seen only upon the *Drosophila* protein knockdown and that we could achieve knockdown (verified by qPCR, Fig. S3). We were able to track the correct genotypes because we used balancer chromosomes with dominant markers (Table S3).

**Fig 4:**
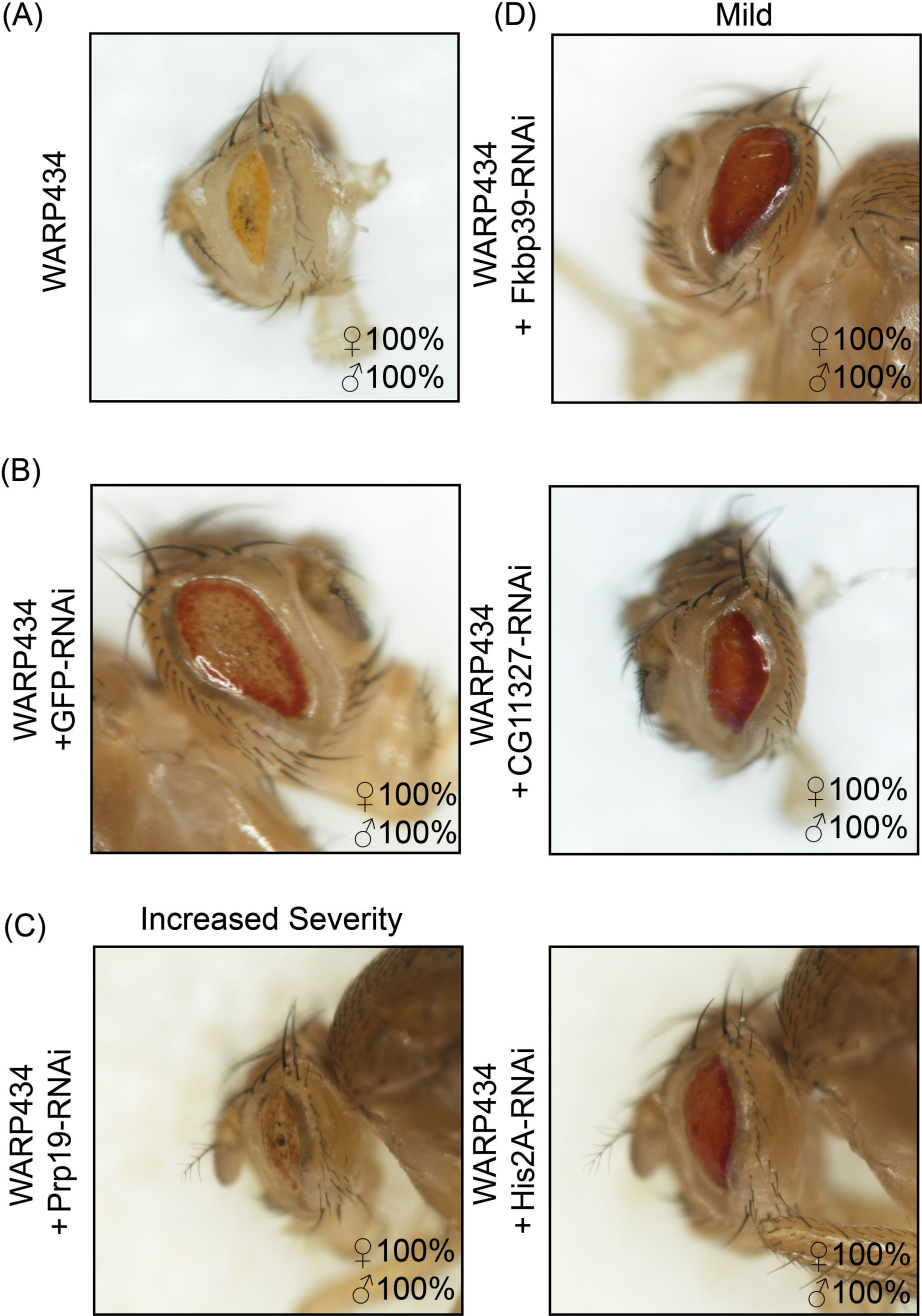
Concomitant knockdown of putative host targets for WARP434 alters observed toxicity in the fly eye. (**A**) Original phenotype induced by expression of WARP434 in the eye; note reduction of pigmentation, rough eye appearance, and reduced size of the eye. (**B**) Knockdown of GFP in the WARP434 expressing background does not recover pigmentation, eye shape, or reduce eye roughness. (**C**) Knockdown of Prp19 in the context of WARP434 expression reduces the eye dramatically, while (**D**) knockdown of Fkbp39, CG11327, and His2A in flies expressing WARP434 recovers eyes with stronger pigmentation over a larger area. Genotype of counted flies shown within each panel; flies were distinguished based on dominant phenotypic markers (i.e., Cyo, Tb, Sb).

**Fig 5:**
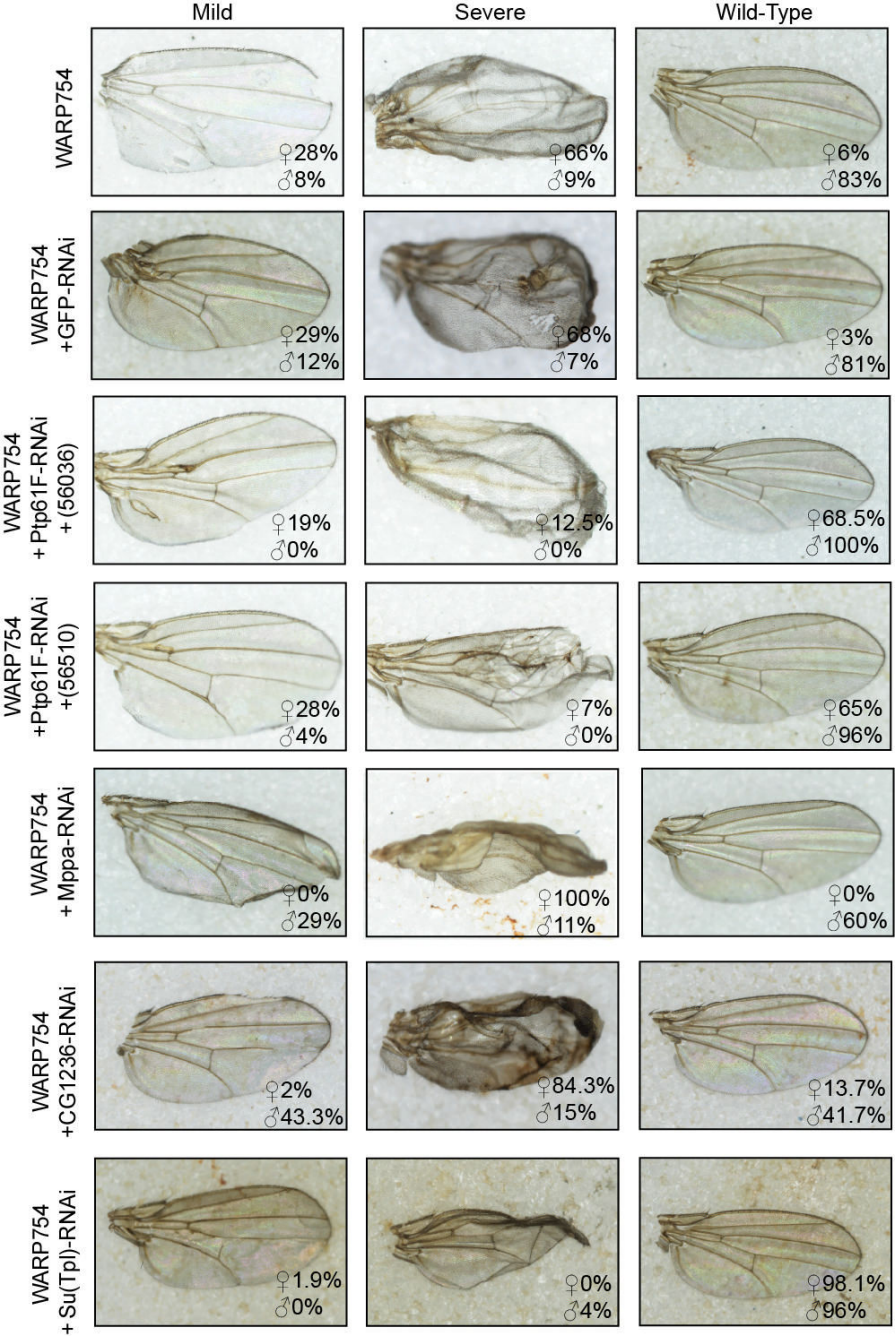
Concomitant knockdown of putative host targets for WARP754 alters observed toxicity in the fly wing. Top, original phenotype induced by expression of WARP754, with proportion of flies with wild-type wings versus those expressing mild or severe wing defects. Below, GFP-RNAi controls show that induction of the RNAi pathway does not alter the original phenotype. Following are RNAi constructs that alter the proportion of flies in each category, with some constructs increasing the fraction of wild-type wings [Ptp61F, Su(Tpl)] and some increasing wing defect severity (Mppa, CG1236). Genotype of counted flies shown within each panel; flies were distinguished based on dominant phenotypic markers (i.e*.*, Cyo, Tb, Sb).

In sum, we were able to knock down 27 putative host targets identified by either the Y2H approach or the coIP (Table S4). In total, 13/27 RNAi knockdowns altered the original toxic phenotypes observed upon expression of WARP434 or 754 in fly tissues (Fig. 4 and 5). Interestingly, we observed a partial recovery of the eye upon knockdown of three targets, CG11327, Fkbp39, and His2A, in the WARP434 expressing line (Fig. 4; Table S4). We interpret this result to mean that WARP434 may alter the function of that protein or increase its activity, leading to misregulation of its associated pathway or other interactions. For one other target—Prp19—we observed an increase in severity under knockdown upon concomitant WARP434 expression. This target was identified from the coIP, and this result could mean that WARP434 functions to reduce the inherent function of Prp19, and the concomitant decrease in protein abundance increases the severity of the phenotype.

For WARP754, we identified 7/15 proteins that altered the fly wing phenotype (Fig. 5; Table S4). An alleviation of the fly wing phenotype was observed for WARP754 expressing flies upon knockdown of Ptp61F and Su(Tpl) in the wing tissue (Fig. 5). Indeed, the fraction of flies exhibiting wild-type wings in the knockdown of Ptp61F and Su(Tpl) along with WARP754 expression increased dramatically from 83% of males and 6% of females to 96%–100% of males and 68%–98% of females. Importantly, the severity of this observed phenotype varied between individual flies. We accounted for this by scoring flies as either mild or severe, depending on whether the observed change was limited to smaller vein aberrations or if it contributed to larger wing blistering. We also observed two cases of increased prevalence of the severe wing phenotype upon knockdown of Mppa and CG1236. We did observe an increase in the percentage of flies with severe wing phenotype from 9% of males and 66% of females to 11%–15% of males and 84%–100% of females. Additionally, we observed females with both wings exhibiting the severe phenotype (60% in CG1236 and 100% in Mppa) contrasted to the one severe wing in the original cross. Collectively, these results strongly suggest that WARPs 434 and 754 do indeed interact with specific fly targets.

### WARPs are expressed by Wolbachia *in vivo*

**Fig 6:**
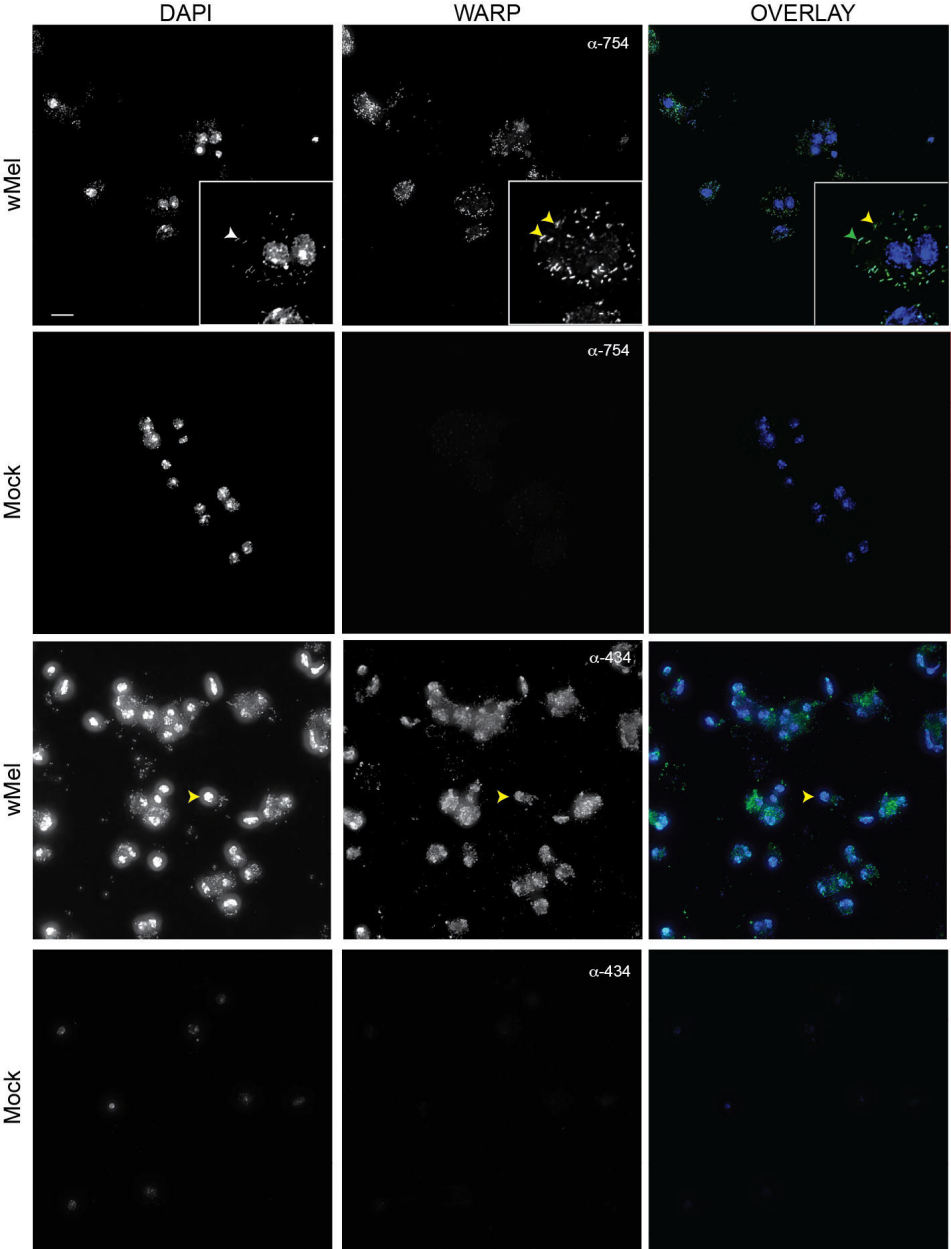
WARPs are expressed by *Wolbachia* during infection of *Drosophila* cells. Antisera against WARPs 434 and 754 detect expression by *Wolbachia* during infection of JW18 cells. Only background staining is visible in uninfected cells (JW18-TET). WARP754 staining (yellow arrowheads) seems to co-localize with *Wolbachia* DAPI staining (white arrowhead in DAPI panel; co-localization indicated by the green arrowhead in overlay panel), but also with other foci within the cell. WARP434 staining seems both localized to the host nucleus (yellow arrowhead) and also diffuse within the cell. Scale bar 10 mm.

If WARPs 434 and 754 are used by *Wolbachia* during infection, we should be able to detect the protein in *Drosophila* cells infected by the bacterium. Toward that end, we used our polyclonal antibodies against full-length WARP434 and WARP754 to perform immunochemistry on a *Wolbachia*-infected *Drosophila* cell line and the uninfected counterpart (Fig. 6). The anti-WARP754 sera was non-reactive in *Wolbachia*-free cells (Fig. 6, JW18-TET) but highly reactive in the presence of *Wolbachia*. For anti-WARP434, we removed non-*Wolbachia* protein-specific staining by purifying the antibody against fly protein blots (described in the Materials and Methods). WARP754 seems to colocalize with DAPI staining of *Wolbachia* in infected cells and also to puncta separate from the microbe (Fig. 6). WARP434 has a more diffuse staining pattern across the cell and could potentially associate with the host nucleus. However, this colocalization requires higher resolution imaging.

### WARP754 is toxic in adult flies

**Fig 7:**
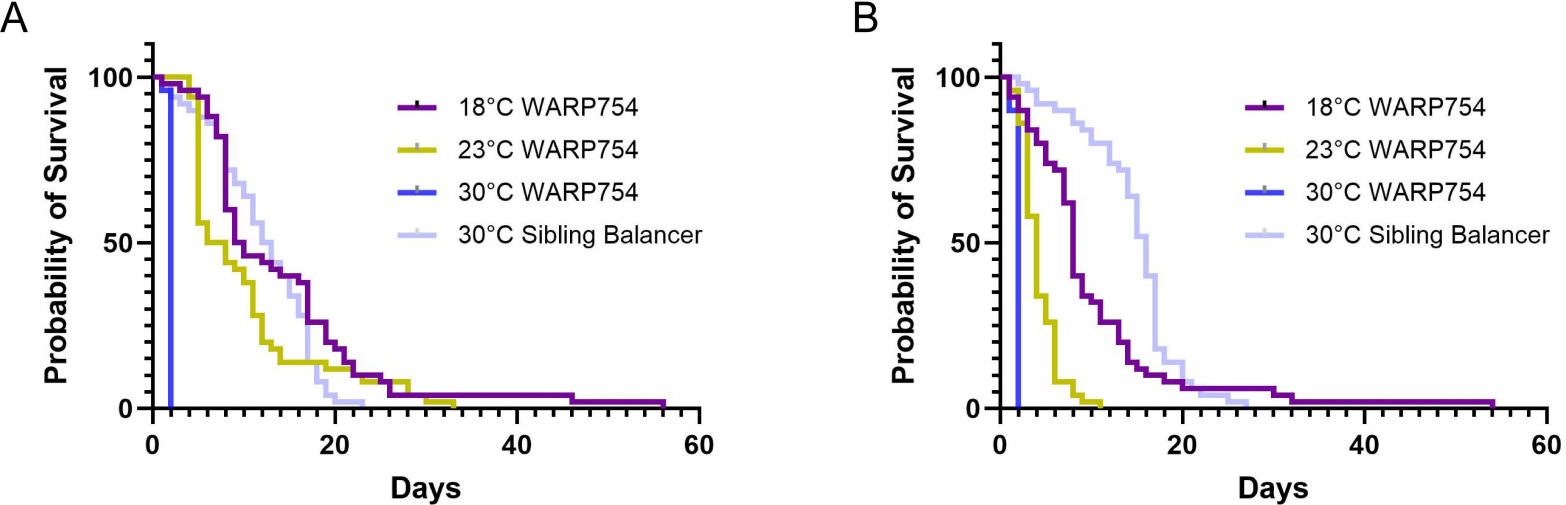
Adult flies quickly succumb when expression of WARP754 is induced. Flies were reared at 18°C under GAL80ts control of expression of the WARP754 construct and either kept at the 18°C control condition or shifted to the 23°C or 30°C experimental conditions upon adult eclosure. Fifty flies were collected per sex for each temperature condition. Sibling balancer flies are shown as a control for the effect of temperature on *Drosophila* lifespan. For both (**A**) female and (**B**) male flies, expression of the construct resulted in a significant reduction in lifespan. Cox proportional hazard model comparing flies expressing WARP754 across temperatures: (**A**) 23°C: 1.506, 0.0451; 30°C: 520.5, *P* < 0.0001, (**B**) 23°C: 4.250, *P* < 0.0001; 30°C: 59.09, *P* < 0.0001. Kaplan-Meier statistics comparing WARP754-expressing flies to sibling balancer controls at 30°C (females: 82.35, 1, *P* < 0.0001; males: 92.10, 1, *P* < 0.001, Fig. S4).

Heterologous expression of WARPs 434 and 754 during fly development is extremely toxic, limiting our ability to interrogate interactions between the expression of these proteins and the fly in the context of the *Wolbachia* symbiosis. We wondered if the toxicity was a result of expression at a specific developmental time point or if adult flies would similarly suffer from WARP toxicity. We therefore used the GAL80ts expression system to dampen expression from the GAL4 transcription factor, allowing recovery of adult flies and induction of global expression after development. The GAL80ts works as a temperature-sensitive transcriptional repressor of GAL4; at cooler temperatures, it binds to GAL4 and prevents it from activating transcription. However, when temperatures are raised, GAL80ts loses its ability to bind GAL4, allowing for GAL4 to activate transcription. Flies containing GAL80ts and the UAS-WARP constructs were crossed to flies infected with *Wolbachia* and carrying the global Actin-GAL4 driver. Crosses were kept at 18°C and progeny at the same temperature until eclosion. Adult flies were then sorted based on genetic markers and kept at 18°C, 23°C, or 30°C, and the survival of adults was quantified. For both WARP constructs, we were able to acquire adult flies at 18°C. Flies expressing WARP434 are indistinguishable from controls when shifted to 23°C or 30°C; however, flies harboring the WARP754 construct quickly perished (Fig. 7; Fig. S4), and this phenotype was exacerbated by increased temperature. Additionally, for adult flies expressing WARP754 at 30°C, we observed a behavioral phenotype whereby adults lose the ability to control their movement (Dryad repository). These results suggest that WARP754 is toxic to flies, both as larvae and as adults.

### WARP754 expression increases Wolbachia titers in adult flies

**Fig 8:**
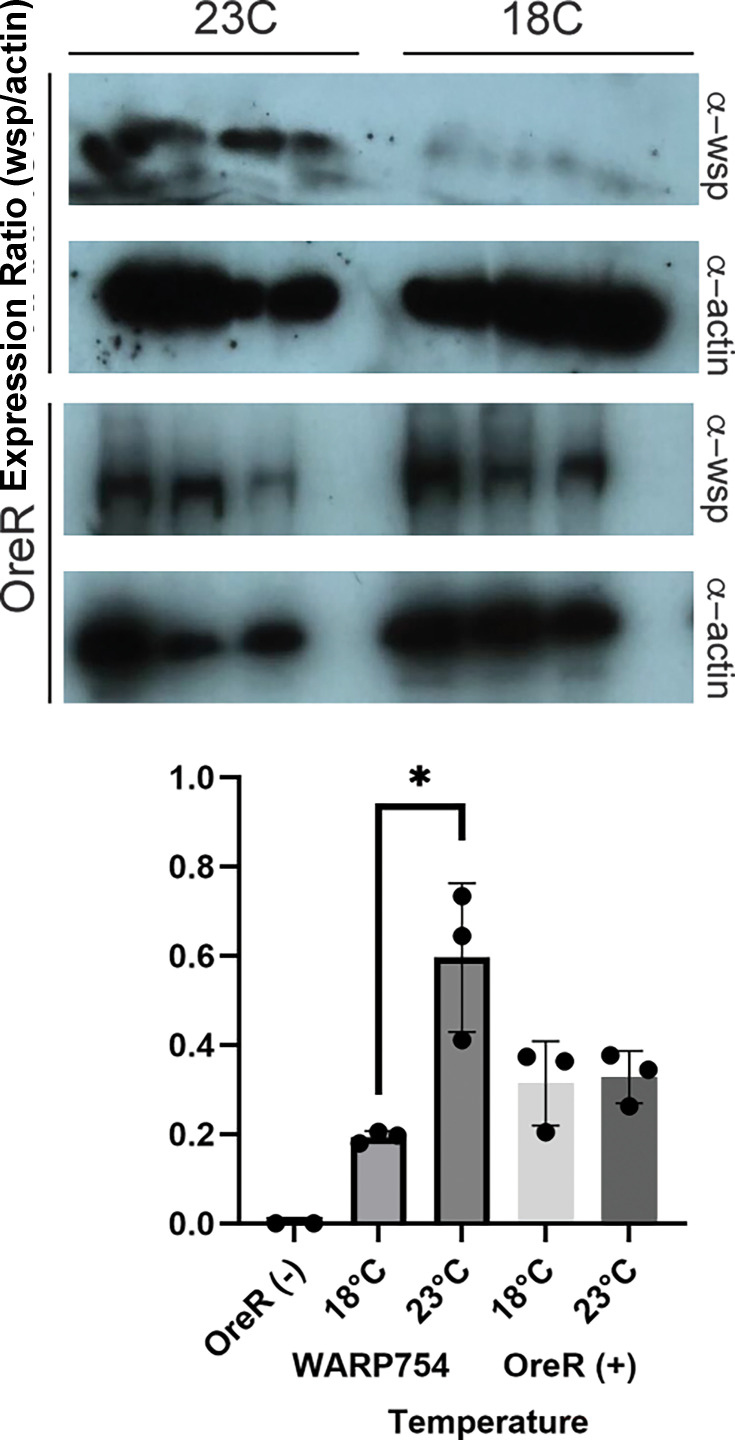
Adult flies expressing WARP754 harbor a higher load of *Wolbachia*. Flies were reared at 18°C under GAL80ts control of expression of the WARP construct, and three groups of three adult female flies for each condition were either kept at 18°C control condition or shifted to 23°C experimental condition upon adult eclosure. OreR(−) flies, which are uninfected with *Wolbachia*, were used as a western blot protocol control, and OreR(+) flies were used as a control for how temperature impacts *Wolbachia* in a wild-type fly. *Wolbachia* titers were measured using the anti-wsp antibody and total host protein by anti-actin. Expression of the construct resulted in a significant increase in *Wolbachia* load. Unpaired t test: (WARP754 18°C vs 23°C; *t* = 4.173, df = 4, *P* = 0.0140) and [OreR(+) 18°C vs 23°C; *t* = 0.2190, df = 4, *P* = 0.8374].

We were able to take advantage of the GAL80ts system to further interrogate the impact of expression of WARP754 on *Wolbachia* titers in whole animals. As before, we kept crosses at 18°C and progeny at the same temperature until adult eclosion. Adult female flies were then sorted based on genetic markers and kept at 18°C or 23°C. Two controls are used in this experiment. First, the same genetic background (WARP754 flies harboring the GAL80ts system and Act5C-Gal4) was reared at two temperatures (18°C or 23°C) to compare the impact of expressing WARP754 on *Wolbachia* titer in a controlled background. As a second control, we also asked whether, regardless of genetic background, we would see an impact on *Wolbachia* titer for rearing temperature (18°C or 23°C). We used an antibody specific to *Wolbachia*, anti-wsp, to infer *Wolbachia* titer when normalized to fly host actin as a loading control. Wild-type, infected flies (OreR+) reared at either 18°C or 23°C did not exhibit any significant difference in *Wolbachia* load, as measured by western blot using anti-wsp as a proxy (unpaired t test, OreR(+) 18°C vs 23°C 1.747, 4, 0.1556) Flies expressing WARP754 at higher temperature, however, increased *Wolbachia* titers dramatically (unpaired t test, WARP754 18°C vs 23°C; *t* = 4.577, df = 4, *P* = 0.0102) (Fig. 8).

## DISCUSSION

Here, we present our discovery of novel host–microbe interactions in the *Wolbachia* symbiosis. The *Wolbachia* genus is a rich source of novel candidate effectors and bacterial toxins, as the microbes are extraordinarily diverse and have proliferated across huge swathes of arthropod and nematode hosts. *Wolbachia* species harbor a huge number of ankyrin repeat domains (29), with some strains encoding upward of 80 WARPs in a 1 Mb genome. Ankyrin repeat domains are known to mediate protein/protein interactions in microbial pathogens of eukaryotic hosts, so they most likely play a role in *Wolbachia* symbiosis. Moreover, WARPs might allow us to discover new aspects of cell biology in arthropods, as the co-evolution of bacterial protein effectors and their targets has led to significant discoveries of basic cellular biology. For example, novel protein modifications, such as AMPylation (50), were discovered by studying bacterial effectors. In the case of *Wolbachia*, the large number of WARPs harbored by this bacterial symbiont has captivated the imagination of scientists in the field (7), with many suggesting an involvement in reproductive parasitism (51–53). Indeed, WARPs are present in every *Wolbachia* genome sequenced to date and related microbes (*Anaplasma, Ehrlichia, Orientia, Rickettsia*) all use ankyrin repeat domain-containing proteins to interact with their hosts and facilitate infection (29). Although the characterized effectors involved in cytoplasmic incompatibility (CifAB) (54), parthenogenesis (PifAB) (55), or male killing (wmk) (56) do not contain ankyrin repeat domains, that does not mean that all *Wolbachia* effectors are ankyrin-free. Importantly, the male-killing protein Oscar recently identified from a *Wolbachia* strain infecting *Ostrinia scapulalis* moths is a WARP which targets the host Masculinizier (57). Additionally, there is much about *Wolbachia*’s basic biology that has yet to be discovered, beyond the reproductive manipulations that have made the genus infamous; many secreted effectors are likely involved in surviving the host cell environment and facilitating the symbiosis (9, 45). Here, we show that two WARPs encoded by *Wolbachia* strain *w*Mel, WARP434 and WARP754, cause severe toxic phenotypes upon overexpression and in combination with a Y2H screen and coIP, identify host targets of these WARPs. We use the toxic phenotype to confirm these targets through suppression of toxicity upon knockdown, suggesting that indeed, these are bona fide interactors of *Wolbachia* WARPs. This is the first large-scale characterization of *Wolbachia* ankyrin repeat domain-containing proteins and their host targets.

What is known about these two *Wolbachia w*Mel-encoded WARPs? WARP434 is understudied in the field, with no publications we could find addressing its putative function or evolution in *Wolbachia*. In contrast, WARP754 has been studied in the context of expression in whole flies (58), as a putative secreted effector (11), for homology across *Wolbachia* and variation in ankyrin domain architecture, SNP content, and pseudogenization (32, 53, 59–61), and putative role in cytoplasmic incompatibility, for which none was found (51). WARP754 was previously reported to have homology to the IncA protein of *Chlamydia trachomatis* (8); however, no such homology could be supported using current databases. Although our staining suggests localization of WARP754 to the *Wolbachia*-containing vacuole, further work is required to interrogate WARP754’s localization and to determine if interaction with Ptp61F or any other host target occurs on the vacuolar surface. Additionally, our work shows a higher phenotypic penetrance upon expression of WARP754 in the wing pouch in females compared to males (71% vs 9%). One possible explanation for this phenomenon is the increased expression of Ptp61F in female flies, according to the modENCODE project (62). Further research is necessary to interrogate any sex-specific effect for WARP754.

Our results suggest that the ankyrin repeat domains of WARPs facilitate binding to *Drosophila* targets and that this binding is necessary for inducing the observed toxicity. We come to this conclusion because expression of the ankyrin domain alone recovered interactions with *Drosophila* proteins via Y2H and because the ankyrin deletion construct in flies did not induce any toxicity whatsoever (Fig. 2 and 3). Many bacterial effectors use ankyrin repeats to bind to their host targets (47). For example, crystal structures of the *Legionella* effector AnkX bound to the Rab1 substrate suggest it uses its ankyrin repeats to mediate that specificity (63, 64), although importantly, associated domains in these multi-domain secreted effectors may also mediate toxicity (65). For WARPs 434 and 754, the ankyrin repeat domain seems to be sufficient for binding of CG11327 and Ptp61F (respectively) and necessary for induction of the toxic phenotype upon overexpression. Importantly, in our screen, we used the fly model to induce severe phenotypes and facilitate genetic dissection of WARP–host interactions; there is no evidence that these WARPs cause these specific phenotypes in these tissues when secreted by *Wolbachia*. All targets identified herein are expected to be expressed in tissues/organs colonized by *Wolbachia* (based on data from the modENCODE project on Flybase), but when and where these proteins are targeted by native WARPs 434 and 754 is still not known. In sum, the evidence presented here from WARP bioinformatic predictions, microscopy evidence for expression, protein–protein interaction, genetic screens, and phenotypic analyses suggests that these two host proteins are relevant for *Wolbachia*-host symbiosis.

Like WARP434 and WARP754, the targets of these two ankyrin repeat proteins are similarly understudied. Out of the eight targets we verified using RNAi knockdowns in our screen, two are uncharacterized *Drosophila* genes (CG11327, CG1236), while the others do not cluster by any cell biological category or mechanism [Ptp61F, Su(Tpl), Fkbp39, His2A, Prp19, Mppa]. CG11327, a target for WARP434, is an uncharacterized *Drosophila* gene for which few publications exist. It has been described as having weak similarity to transcription factors (66), but beyond this, little else is known regarding its function. For Ptp61F, because of its homology to mammalian proteins, we know a bit more about its putative functions. It is characterized as a non-receptor protein tyrosine phosphatase that negatively regulates JAK-STAT, insulin-like receptor, EGFR, and Pvr pathways, impacting fly fecundity, growth, and lifespan (67), organ size (68), but also coordination of the actin cytoskeleton (69). These functional connections are particularly exciting and relevant for *Wolbachia,* as infection by the microbe has been associated with changes in *Drosophila* fecundity and lifespan, as well as metabolism and the actin cytoskeleton (70–72), and our results provide a clear target for future mechanistic work determining *how* the microbe alters cell biology to achieve this organism-wide effect. Does a WARP modify a host target or alter its localization? Does a WARP inactivate the protein, increase its native activity, or expand its functional repertoire? And what are the ramifications for *Wolbachia?* These questions are the subject of future work in our laboratory.

### Conclusion

We have shown that WARPs bind directly to specific fly proteins, consistently inducing observable and quantifiable fly phenotypes. This work identifies direct interactions between *Wolbachia* ankyrin repeat proteins and host targets, setting the stage for more mechanistic dissection of this important symbiosis.

## MATERIALS AND METHODS

### Prediction of secreted substrates

All *Wolbachia pipientis w*Mel WARPs were used as input to BastionX via the BastionHub (44). BastionX is based on a set of independently trained binary machine-learning predictors, with each predictor trained to estimate the probability that a given protein belongs to a specific secretion substrate class. Each predictor outputs a score between 0 and 1, which can be interpreted as the model-estimated probability that the protein is a substrate of that particular type. Importantly, all predictors were trained using a subset of the same negative examples, which makes the resulting scores directly comparable across substrate classes. Conceptually, if a protein truly belongs to a given substrate class, the corresponding predictor is expected to assign it the highest score (Jiawei Wang, first author of BastionX [44], personal communication).

### Fly stocks, generation of UAS lines, and maintenance

All flies were grown at 23°C with a 12 h light-dark cycle with lights on at 9 a.m. and off at 9 p.m. on standard cornmeal fly medium. For a full list of flies used in this study, see Table S3. Each open reading frame (ORF) predicted to encode at least one ankyrin repeat motif (HHpred and AlphaFold citation) from the *Wolbachia w*Mel strain genome (REF) (WD_0035, WD_0147, WD_0191, WD_0206, WD_0285, WD_0286, WD_0291, WD_0292, WD_0294, WD_0385, WD_0434, WD_0438, WD_0441, WD_0498, WD_0514, WD_0550, WD_0566, WD_0596, WD_0633, WD_0636, WD_0637, WD_0754, WD_0766, WD_1213, RS04160) was codon optimized for expression in *Drosophila melanogaster* in Geneious V2021.2 and synthesized at Genewiz Inc. with 5′ and 3′ Gibson tails compatible with plasmid pJFRC7 Fasta Supplemental File, with the exception of WD_0633, which was sent as a gift from Dr. Seth Bordenstein (Vanderbilt University) in a *D. melanogaster* expression plasmid containing a *D. melanogaster* codon optimized version of ORF WD_0633. The 20X UAS expression vector pJFRC7 (Addgene Plasmid #26220) was Midi prepped (Qiagen Cat. No./ID: 12843) and digested with XhoI and XbaI to remove the mCD8::GFP insert. The resulting 8,145 bp band was separated on a 1% agarose gel and gel extracted (NEB Cat. No. T1020S) for Gibson Assembly (ThermoFisher Cat. No. A46624). Due to cloning difficulties from highly repetitive sequences, some *w*Mel ankyrin repeat genes (WD_0147, WD_0438, WD_0596, WD_0636, WD_1213) were cloned into the 20X UAS expression vector pPMW-attB (Addgene Plasmid #61814) via Gateway cloning. Briefly, synthesized dsDNA gene fragments were designed with 5′-CACC to facilitate ligation with the pENTR vector (ThermoFisher Cat. No. K240020). Products were transformed into chemically competent OneShot Top10 *E. coli* (ThermoFisher Cat. No. C404003), and three positive colonies were Mini prepped (Qiagen Cat. No./ID: 27106) and Sanger sequenced to confirm the presence of each insert. Positive pENTR plasmids were combined with LR Clonase II Enzyme Mix (ThermoFisher Cat. No. 11791020) and the destination vector pPMW-attB, transformed, and screened as described above. All positive plasmids were shipped to Rainbow Transgenics Inc. for injection into *D. melanogaster* embryos expressing the integrase PhiC31 to facilitate attP-attB integration into landing site attP40 (chr II) or attP2 (chr III). After being injected, P0 flies eclosed, they were crossed to siblings, and red-eyed F1 progeny were selected and crossed to keep as homozygous lines. All plasmid and Genewiz-synthesized gene sequences can be found in File S1.

### Ankyrin deletion lines

Ankyrin deletion 20X UAS expression constructs for ORFs WD_0434 and WD_0754 were created to test the necessity of ankyrin repeats in host toxicity. To identify the N- and C-terminal bounds of each ankyrin repeat, we aligned the conserved 33 amino acid ankyrin repeat motif NGRTPLHLAARNGHLEVVKLLLEAGADVNAKDK (73) with WD_0434 and WD_0754 amino acid sequences. To confirm the presence of each repeat motif, protein structure predictions were generated via AlphaFold (74, 75), and the resulting.PDB files were submitted to AnkPred (42). We identified two ankyrin repeat motifs in WD_0434 and four ankyrin repeat motifs in WD_0754. DNA sequences with these motifs removed (File S1) were codon optimized for expression in *D. melanogaster* in Geneious V2021.2, synthesized, cloned into pJFRC7, and injected into *D. melanogaster* as described above.

### Yeast-2-hybrid assays

Y2H assays were carried out using strains, media, and techniques described in reference 49. Briefly, due to toxicity, ORFs containing just the ankyrin repeat motifs from WD_0434 and WD_0754 were Gateway cloned into the destination vector pDest-DB-cen to create N-terminal fusion constructs of the *Saccharomyces cerevisiae* GAL4 DNA-binding domain (DB) with C-terminal ankyrin repeats (File S2). Primers (Integrated DNA Technologies) containing 5′-CACC to facilitate ligation with the pENTR vector (ThermoFisher Cat. No. K240020) were designed to amplify the ankyrin repeat regions from our *D. melanogaster* codon-optimized 20X UAS pJFRC7 constructs (described above). The resulting amplicons were column-purified (Qiagen Cat. No./ID: 28104) and cloned into the pENTR vector (ThermoFisher Cat. No. K240020). Products were transformed into chemically competent OneShot Top10 *E. coli* (ThermoFisher Cat. No. C404003), and three positive colonies were Mini prepped (Qiagen Cat. No./ID: 27106), and Sanger sequenced to confirm the presence of each insert. Positive pENTR plasmids were then combined with LR Clonase II Enzyme Mix (ThermoFisher Cat. No. 11791020) and the destination vector pDest-DB-cen and again screened via Sanger sequencing for each insert. The resulting pDest-DB-cen ankyrin constructs were then transformed into the yeast *S. cerevisiae* strain Y8930 and screened using techniques described in reference 49.

The initial Y2H primary screen was designed to identify *D. melanogaster* protein–protein binding partners for the ankyrin repeat motifs in WD_0434 and WD_0754. Dr. David Hill and Kerstin Spirohn (Dana-Farber Cancer Institute) kindly gifted the *S. cerevisiae* activation domain (AD) library (*Drosophila* ORFeome) as a collection of 118 glycerol plates containing 10,490 AD-*Drosophila* ORF fusion strains (File S1) representing ~2/3 of the *D. melanogaster* proteome (https://flybi.hms.harvard.edu/). A pool containing both Y8930 DB-ankyrin strains for WD_0434 and WD_0754 was screened for autoactivation by mating to a pDest-AD-empty Y8800 strain. After 96 h at 30°C, we found no evidence of growth on Y2H selective media containing 1 mM 3AT (3-amino-1,2,4-triazole; Sigma-Aldrich A8056), indicating no autoactivation (49). Using an Integra Biosciences Assist Plus pipetting robot, the pool was then mated against 10,168 Y8800 AD-*Drosophila* ORF strains (322 strains did not grow up from the glycerol stock; Table S1) plated on Y2H selective media containing 1 mM 3AT, and grown at 30°C for 72–96 h. AD strains that showed signs of growth when mated with the ankyrin DB pool were then re-mated in a secondary screen against the separated Y8930 DB-ankyrin strains to identify putative binding partners for WD_0434 and WD_0754 ankyrin repeat motifs.

### *Drosophila* melanogaster cell culture

*Drosophila melanogaster* embryonic JW18 or JW18-TET (45) cells were maintained as described by Martin et al. (45). In brief, cells with (JW18) and without *Wolbachia* (JW18-TET) were maintained in T25 unvented cell culture flasks, in a dark drawer at room temperature in Schneider’s insect medium with L-glutamine and sodium bicarbonate, supplemented with 10% heat-treated fetal bovine serum and 1% penicillin/streptomycin solution. S2R+ were maintained the same as JW18 cells (DGRC Stock 150; https://dgrc.bio.indiana.edu//stock/150; RRID:CVCL_Z831).

### Antibody production

Full-length *w*Mel WD0754 (WARP754) and WD0434 (WARP434) protein-encoding constructs were synthesized by GenScript using codons optimized for *E. coli* and subcloned into the pET30a vector with a 6xHis tag at the N terminus, in BL21 Star (DE3). Cultures were grown in LB medium containing kanamycin at 37°C and 200 rpm. Once cell densities reached OD600 = 0.6–0.8, 0.5 mM IPTG was introduced for induction, and cells were shifted to 15°C for 16 h. The proteins were purified using Ni-NTA columns (Sigma-Aldrich) and subsequently sent to Cocalico Biologicals for antibody generation. Pre-inoculation sera from four rabbits were screened on blots of whole fly protein with and without infection, and the rabbits with the lowest background reactivity were chosen for inoculation. Rabbit polyclonal antibody sera against both full-length purified proteins were generated (Cocalico Biologicals, Inc.) and used separately for immunochemistry (see below).

### Co-immunoprecipitation and mass spectrometry

We used Protein A/G Magnetic Bead (Pierce, Thermo Scientific Catalog #88802), following the manufacturer’s procedure for manual immunoprecipitation with slight modifications, as noted here. Initial bead preparation used 50 µL of bead slurry per sample, washing as indicated. We added 50 µL whole anti-WARP754 sera of the antibody (or 50 µL pre-bleed whole sera, when applicable) in 450 µL binding buffer to the beads for 1 h at room temperature with mixing, or at 4°C overnight. For the cell lysate, we used JW18 cells with a native *Wolbachia* infection and cells cleared of the infection, JW18-TET. Cells were counted, washed twice in sterile PBS, and pelleted. Each pellet of 7.5 × 10^6^ cells was lysed by 5 mL of lysis buffer (150 mM NaCl, 50 mM Tris-HCl, pH 8.8, 10% glycerol, 1% Triton, 1 M Arginine), with Protease Inhibitor included (1 tablet per 10 mL of Thermo 88665), and held on ice for 10 min with periodic vortexing. Antibody prep was removed from the magnetized beads and replaced with 1 mL of lysate and held at 4°C overnight with rotational mixing. Beads were washed per protocol, transferred on the last wash to fresh tubes, and left on the beads for mass spec analysis. Beads were stored in 50 µL 25 mM ammonium bicarbonate solution. For WARP434, our handling of the beads was identical to the above protocol, but our optimization for WD0434 enrichment required fractionation of the samples before protein lysis. We followed Senichkin et al. protocol (76) for nuclear and cytoplasmic fractionation, specifically outlined in their Appendix A for hypotonic and isotonic solutions, with the addition of NP40 for lysis as indicated before adding to the beads (prepared as above) for enrichment. Lysate was added to the beads for enrichment as above. This time, each bead sample was split into nuclear and cytoplasmic fractions, soluble and insoluble (pellets) for each. Pellets of each fraction were lysed in lysis buffer (150 mM NaCl, 50 mM Tris-HCl, pH 8.8, 10% glycerol, 1% Triton, with Halt Protease Inhibitor (1×) (Thermo Scientific #78430, without EDTA added) before bead addition. The washed beads, stored in 50 µL 25 mM ammonium bicarbonate solution, were submitted for analysis to the Indiana University Laboratory for Biological Mass Spectrometry. Peptides from *Drosophila melanogaster* and *Wolbachia pipientis* were identified and analyzed by LC-MS on an Orbitrap Fusion Lumos Tribrid equipped with an Easy NanoLC 1200.

### Western blots

Flies were ground in 1.5 mL centrifuge tubes using an electric hand drill and disposable pestles in lysis buffer: 150 mM NaCl, 1% Triton X-100, 50 mM TrisHCl (pH 8) containing HALT protease inhibitor cocktail (Thermo Scientific) and 5 mM EDTA. The lysates were centrifuged for 1 min at 8,000 × *g* to pellet debris. Samples were heated for 5 min at 95°C in Laemmli sample buffer containing 5% β-mercaptoethanol (Bio-Rad) prior to SDS-PAGE electrophoresis. Proteins were separated on 4%–20% Tris-Glycine NB precast gels (NuSep) in 1× Tris/Glycine/SDS running buffer (Bio-Rad) and transferred to PVDF membrane in Tris-Glycine transfer buffer with 15% methanol at 40 V on ice for 3–4 h. The membrane was blocked for 5 min in Starting Block T20 (TBS) Blocking Buffer (Thermo Scientific), followed by incubation in primary antibody (for 1 h at RT or O/N at 4°C) according to standard protocols. SuperSignal West Pico Chemiluminescent Substrate (Thermo Scientific) was used according to the manufacturer’s instructions to detect HRP (after incubation with secondary antibodies) on the immunoblots. Blots were re-probed after stripping in 100 mM Glycine, 0.15 ND-40, 1% SDS, pH 2 for 1 h at RT, then overnight at 4°C. PageRuler prestained protein ladder (Thermo Scientific) was used as a molecular mass marker. The following antibody was obtained through BEI Resources, NIAID, NIH: Monoclonal Anti-Wolbachia Surface Protein (WSP), NR-31029, and used at a dilution of 1:1,000. Additionally, we used anti-actin monoclonal at 1:10,000 (Seven Hills Bioreagents) as a loading control, as well as secondary antibodies: HRP enzyme conjugates (Invitrogen) at 1:5,000. Densitometry measures were made in ImageJ using scanned film with the same exposure times across multiple experiments.

### Drosophila melanogaster cultured cell immunochemistry and microscopy

The *Drosophila melanogaster* JW18 cells infected with *Wolbachia* strain *w*Mel or tetracycline-cleared counterparts (JW18-TET) were used to visualize the location of antibodies to native WARP434 and WARP754 (77). Monolayers grown to confluence were harvested from 25 cm^2^ non-vented tissue culture flasks, and cells were counted using disposable hemacytometers (Fisher Scientific). Cells were overlaid as 100 μL of 2 × 10^6^ or 200 μL of 1 × 10^6^ cells onto Concanavalin-A-coated 22 mm square No. 1.5 coverslips (ConA 0.5 mg/mL applied and dried onto sterile, acid-washed coverslips and leaving a 2 mm ConA-free border) (78, 79). Cells were allowed to settle, attach, and grow for two nights. Cell-coated coverslips were transferred to 6-well tissue culture plates, one coverslip/well, cell-side facing up, and the coverslips were washed twice in 500 μL of 1× PBS before cells were fixed in 4% paraformaldehyde in 500 μL PBS for 20 min, followed by four washes of 500 μL 1× PBST (0.2% Tween-20 added to 1× PBS). Coverslips were then incubated in 1 mL blocking solution (PBST 0.2% Tween-20 and 0.5% BSA) for 1 h at room temperature before replacing the block solution with primary antibody dilutions overnight in blocking solution at 4°C. Primary antibodies to *w*Mel proteins were rabbit anti-WD0434 pre-adsorbed for 6 h at 4°C against PVDF-blotted uninfected S2R+ (80) total protein lysate and used at 1:500 dilution; and rabbit anti-WD0754 purified with Thermo Scientific Melon Gel IgG Spin Purification Kit # 45206 and used at 1:500 dilution. In the morning, coverslips were washed three times for 5 min each with 1 mL of 1× PBST and then allowed to incubate for 2 h at room temperature in the dark in 100 μL of secondary antibodies diluted 1:1,000 in the blocking solution [Thermo-Fisher A-31573 Donkey anti-Rabbit IgG (H+L) conjugated to Alexa Fluor 647]. Coverslips were washed for 5 min three times with 1 mL PBST and then were incubated in 10 μg/mL DAPI solution (Sigma-Aldrich, D8417) for 30 min before washing three times with 1 mL of PBS, followed by a final dip in a 500 mL beaker of distilled water. Excess moisture was wicked off the edge of the coverslip with a tissue, followed by mounting in 10 μL of Prolong Gold Antifade Reagent with DAPI (Invitrogen, P36935) per coverslip on glass slides. Images were taken as Z-series stacks at 0.2 micron intervals using a Nikon Ti2 fluorescent microscope with a 100× oil objective and processed using NIS Elements software (Nikon). Exposure times and stack intervals were equivalent across the compared experimental conditions.

## Data Availability

Raw FASTA files are included as supplementary materials with this journal submission. Complete Excel spreadsheet of full coimmunoprecipitation results, as well as movies of fly behavior, are uploaded and freely available on Dryad (link here).

## SUPPLEMENTAL MATERIAL

Data S1 mbio.00172-26-s0001.xlsx Full coIP results for WARP434.

Data S2 mbio.00172-26-s0002.xlsx Full coIP results for WARP754.

Supplemental material mbio.00172-26-s0003.docx Figures S1-S4; supplemental table and file descriptions.

Supplemental sequences mbio.00172-26-s0004.docx Sequences of all constructs generated in this project.

Table S1 mbio.00172-26-s0005.xlsx *Drosophila* orfeome set, including full CDS sequences and plate locations.

Table S2 mbio.00172-26-s0006.xlsx Curated hits from the coimmunoprecipitation of both WARP434 and WARP754.

Table S3 mbio.00172-26-s0007.xlsx Full list of fly stocks generated and used in this study.

Table S4 mbio.00172-26-s0008.xlsx Full list of RNAi knockdown experiments for each putative *Drosophila* target, including phenotypic outcomes and counts for each.

ASM does not own the copyrights to Supplemental Material that may be linked to, or accessed through, an article. The authors have granted ASM a non-exclusive, world-wide license to publish the Supplemental Material files. Please contact the corresponding author directly for reuse.
