# Supplementary material for: Wolbachia uses ankyrin repeats to target specific fly proteins
Source: mBio. 2026 Apr 20;17(5):e00172-26. doi: 10.1128/mbio.00172-26 (PMC13170367; doi:10.1128/mbio.00172-26)
Supplement: Supplemental material — Figures S1-S4; supplemental table and file descriptions. [file mbio.00172-26-s0003.docx]

Supplementary Materials

**
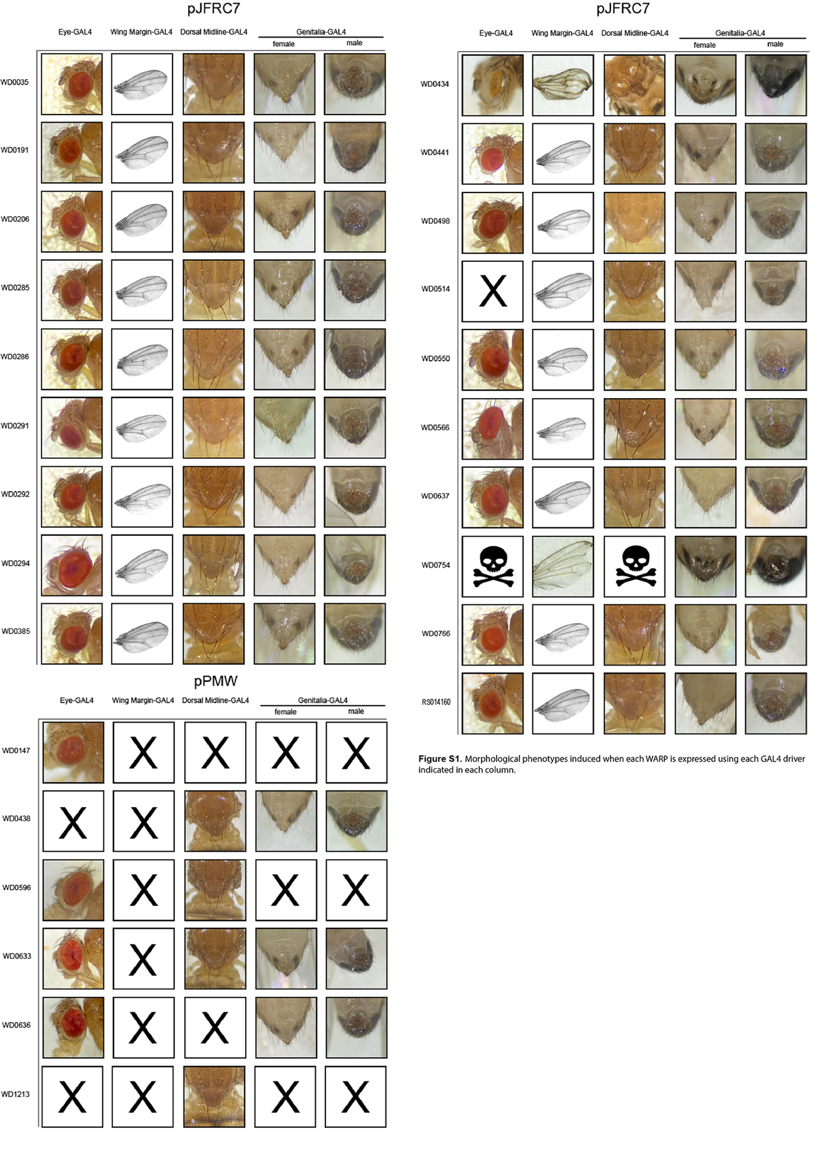
**

**Figure S1** – Morphological phenotypes induced when each WARP is expressed using each GaL4 driver indicated in each column. The majority of Wolbachia WARPs cause no apparent phenotype in Drosophila melanogaster flies upon over-expression in specific tissues. Crosses that were not completed shown with ‘X’.

**
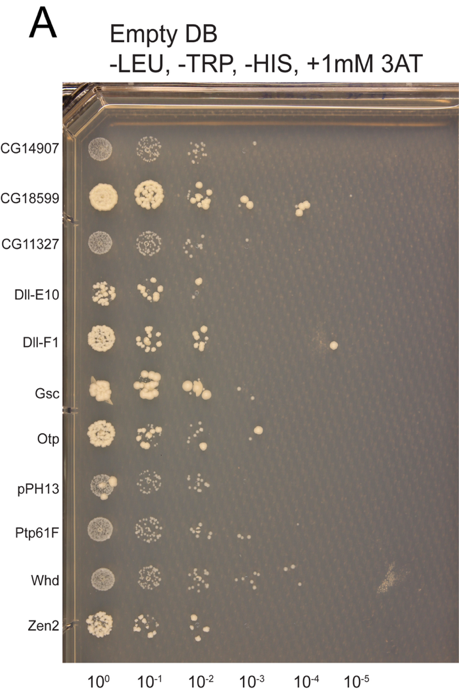
**

**Figure S2** – Haploid yeast carrying certain Drosophila orfeome targets are able to grow in the absence of any interacting partner, indicative of autoactivation. CG18599, DII, Gsc, Otp, and Zen2 all showed significant growth on these plates.

**
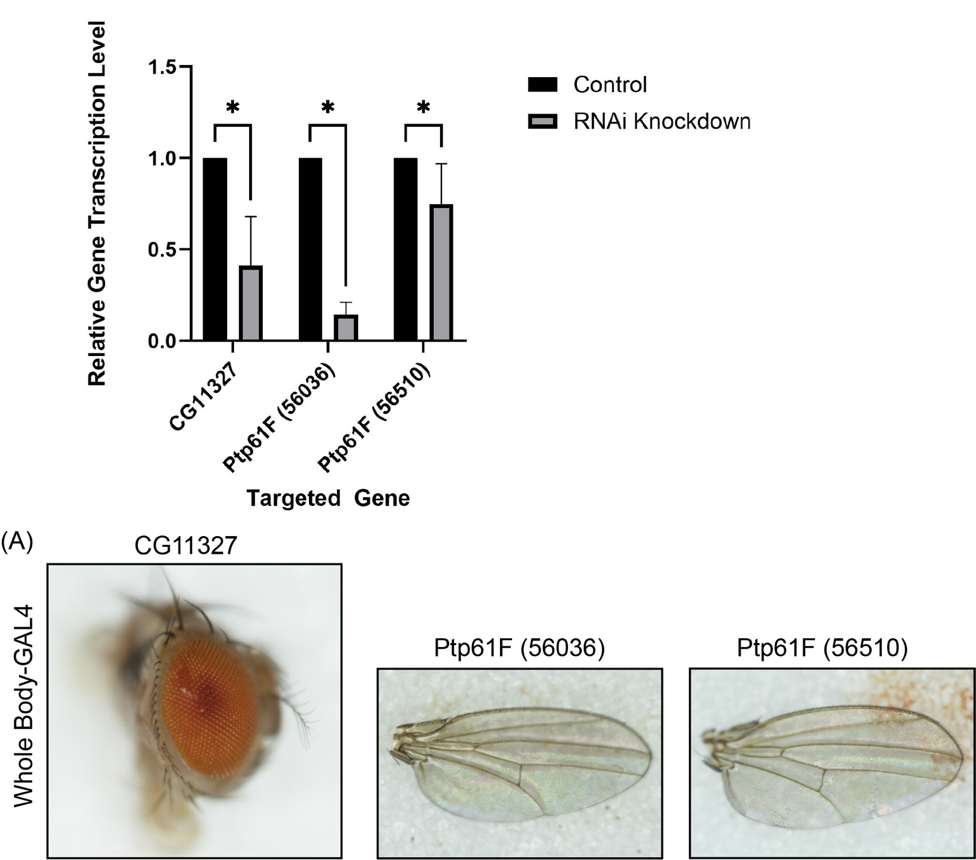
Figure S3** – Confirmation of knockdown of CG11327 and Ptp61F by qRT-PCR (above) and no apparent phenotype upon knockdown of these loci in otherwise wild-type flies (below).

**
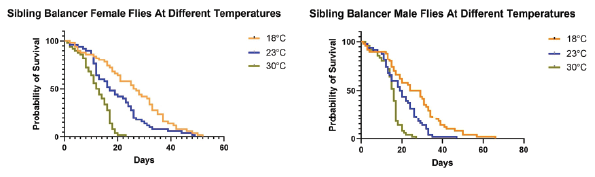
Figure S4** – Flies expressing WARP 754 die prematurely regardless of sex (A – female, B- male). Siblings of flies generated during these same crosses, not expressing WARP754, do not die prematurely upon shift to higher temperature (C - female, D – male).

**Table S1** – Fly AD collection - *Drosophila* orfeome set including full CDS sequences and plate locations.

**Table S2** – Curated hits from the coimmunoprecipitation of both WARP434 and WARP754.

**Table S3** – Full list of fly stocks generated and used in this study.

**Table S4** – Full list of RNAi knockdown experiments for each putative *Drosophila* target including phenotypic outcomes and counts for each.

**Supplementary Fasta Files** – All nucleotide sequences synthesized for expression in *Drosophila* and *Saccharomyces*.
