# Supplementary material for: Wolbachia uses ankyrin repeats to target specific fly proteins
Source: mBio. 2026 Apr 20;17(5):e00172-26. doi: 10.1128/mbio.00172-26 (PMC13170367; doi:10.1128/mbio.00172-26)
Supplement: Supplemental sequences — Sequences of all constructs generated in this project. [file mbio.00172-26-s0004.docx]

Supplementary Information for *Wolbachia* uses ankyrin repeats to target specific fly proteins

Fasta files:

**WARPs.fasta: all nucleotide sequences synthesized for expression in Drosophila and Saccharomyces.**

>gb|AE017196.1|:1-1267782_-_WD_0035_ORDERED,_RECEIVED,_CLONED,_MIDI_PURIFIED_extraction Wolbachia endosymbiont of Drosophila melanogaster, complete genome

ATGACCGAAGAGACCAAGGATGGTTACGCCAGCCTGTACGTCGCCATCCAGGAGGGCAACATCGAGGCCGCTGAGCTCCTGATCAAGTGCGGCACCAACGTGAACGACCACTACGAGCGCAACCGTACTCCCCTGCATATCGCCATCGGCCGCAAGCAGCTGGAGATCGCTAAGCTCTTGATCAAGAACGGCGCCAACGTGAACGCTAAGACCCAGAACCATGGAAAGGACGATCTGACCCCCATGCACTTCGCCGTCTTCGCCAACACCCCAGAGTTCATCGAGTTGCTGGCCAGCCATGGCGCCCTGATCAACGAGCGCGAGAGCACCGAGGGCTACACCCCACTGCACTTCGCTGCCCTGTACGGTAACAAGAACATTATCCAGGCCCTGATCGATAAGGGTCAGGATATCGAAGACGTGGATAACAATGGCCGCACCGCCCTGTTCCTGGCTGCCCGTCAGTGCACCGAGGCCGAGGACGATAGCCGCATCGAGATTATCAAGTACCTCATTGACAAGCTGAAGGCTGACGTCACCAAGAAGGACAATAACAATAACGCCGTGCTGTTCCCCGCTGCCAATAACTGCCCTGGCAAGGTGGTTGAGTTCATTATCGAGCAGTACATCAAGATCTTCGAGCTGGAGAACTTCATCAATCACAAGAATAACGATGGTATGGATGCTCTCGATATTGCCCTCAACAGCGGTAACGAGAAGGCCATCGAGGTCCTCCGTTCCTACGGAGCCGACATCAAGAACAAGGTCGATGGCTCGATCCAAAAGATCACCGCTGAGAAGCCCAGCTCCACCCTGGATCGCGCCGAGTCCGCTGAGGTCGCTCCCCCAATCAAGCAGCGCGTGTAA

>gb|AE017196.1|:1-1267782_-_WD_0147_ORDERED,_RECEIVED_extraction Wolbachia endosymbiont of Drosophila melanogaster, complete genome

CTGTGGCTCATCCTGTTCCTGATGCGCGTGATGGATAAGAAGTGCCCTTTCCGTCTGGCCACCGAGCTGAAGTCTGCTGCCGGCTTCGACGATGTTGTGTTGCAGTGCAAGCAGAACAAGACCACGATCCACCGTTTCGTGCAGGTGAAGCATAAGCAGGACGGAACCGAAAAGATCAGCGTGGGCTCCTTGCTGACCAAGTCGGGCGAATTCAACCTGCTCAAGTACTTCATCGCCTACTTGAAGATTAAGTCTAATGGTAAGTTCAAGGGTGAGATGAAGTACTTCGCCATCGTGACGAACATCGATTTCGATTTCACGGACTCGGCCCAGCACGAGGTTCGCAAGTTGCGCATGATGAGCTCGGGTAAGAACAAGGAGAAGGAGATTTCCGTCATTCGCATCGACACCCAGGATGAGTTCCTGGATGTCGGCGATGGCGTGCGCTACAAGTTCGACAACTCCATTATCTCCTATCTGCAGGAAAACAAGGATTTCATCAAGGGTAAGGTCGGTCGCGAGGTGTCCGACAAGGAGGTGGAGGATTTCCTGAATGAACTGGTCTTCGCCGTTAACCTGCCCAACGAGTCCCAGCTCAAGGAGCTGTTCAAGGGAGAGATGTCCGGTCGTCTGGCCAAGAAGTTCGGCTACGTGGGTGACAACGAGATCTTCTGCAACGATCTGTTGGAAAAGATTTCCGATTGGGTGAAGGACATCAAGGGTCGCTTCCTGTCCCCCGAAGAGGGAAAGGAGTTTTTCCAGAAGGTCGAGCTGTGGGCCTCCACCCTGTGCGGCATCGAGCGTGGCGTGAAGAAGGGTAACAAGGGTATCGACAAGATCGCCAAGCAACAGCACAAGGACTCGAAGACCTTGAACGAGATTCATGCTGCCGTGACCGCCCAGAATAACAAGCAGCGCCCACACAAGAAGCCGTACGGCCCCCGCGATAAGAAGTCCAAGGCCCGCGAAGAGGGCGACATTGAAGAGTCGGACGAGTTCGATGAAATTGAGGCCCTGGAGTTCGTGAAGCGCGCCCTGAACATCGAGAATAACCTGCAGAACAAGGAGATCAAGAAGTTGGCCCAAGAACTGCAGTACTCCCAGCTGGCCCTCAAGCTGGCCGTTACCTACATCTCCGAAGAGAACATCGTGTTCTCGCACCGTGGTCGTAAGCGCATCCGCGTGGGCGGTTACTTGAAGAAGTGCGAGAAGATCGCTGAGAAGTTGCTGGATTTCAAGTCCGAGTACAAGTCGGACCGTTATGCCAAGGCCACCTTCATCACCTGGAAGATTACCATCGACGCTATTGTGCAGAAGAAGTTCCGTCCCGAGGCCCTGTCGATTCTGGAGATCATGGCCTACTTCTCCCCCAACGAGATCTACATCGAGGAAATCTTCAGCCAACAGGTGGCTAACGATAAGGAAGCTCTGTGGAACGCCGTCGAACTGCTCAACCGCTACTCGATGATCAAGCTGCGCAAGGGCGTTGTGAACATCTACCGCCTGGTCCAGAAGGTCACCCGCCTGAAGCTGCAGGAGAAGGGTCGCGAGGAAGAGGTGCTCCGCAAGGCCCTGGAGCTGATCAATAGCTACGACGTGCTGATCGACAACTCGATCGTGGCCTCGGTCTGGGGCTACGTCTCCAAGTACGGAGAACTGATTGACGAGTTCTACTTCAACCCCATCTACGGTAAGTGGAAGTACACCCCTCTGCACTCCTTGGCCGAATCCGGCTCTTACGAAGCCGTCCGCTGCGTCTTGACCCACATGGAAGAGAAGCACCCGGATAAGTTCAACTGCGTGGTTAATGCCCGCGATGGCTCCGATTCGACCCCTCTGTGCTCCGCCGTGGAGAGCGGCGGACTGGATATCGTCCAGTACTTCATCAACAAGGGCGCCGATGTCAACGCTAAGTCCCGCTACGGAATCACTCCCCTCCACCAAGCCGTGTACGATGGACGCGTCGACATCGTTGAGTACCTGATCGGTAAGGGCGCCGACATCAACGCTAAGGATGAGTCGGGTTTCACCGCCCTGCACTGGGCCACCATGATGTATCGTGTGGATGTGGCTAAGGTCTTGCTGAAGCATAACGCCGACGTTAACGCCAAGGACAAGGATGGCGATACCTCGCTGCACCTGGCCACCAAGATGGGCCGTGTCGCCGTCGCCAAGGTGCTGCTCGAGCACAACGTCGACGTCAATGTGAAGAACGAGCAAAATCGTATCTCCCTGCATTACGTGGCCCGCAGCGGCAGCATCGAGACGATCGAATGCCTGATCGAGAAGGGCGCCGACGTGAACGCCAAGGATGAGAACGGCAACACTCCCCTGCACTTCGCTGCCATTATGGGCAACTTCGATACCGCCCGCGTTCTGCTCAAGCACAACGCCGATGTTGATACCAAGAATAACCGCGGAATGACTGCTCTGCATTATGCTACCGATTTCGACCATCAGGAGCTGGTTGAGTTGCTGTCCGCCCGCGATACCAACAGCATCGATGACGGTATGTGCATGGTCGCCCTGGCCAAGACGTTGCTGAAGCACACGACCAACGTGTCCGTCAAGAACGAGTGCAACAAGACGCCCCTCCATCACGCTGCCAAGATTGGCTCGGAGAAGCTCACCAAGTACTTCATTAAGGAGGGTGACGATGTCAACGCCAAGGACGAGAACGGAAACACTCCCCTCCATTTCGCTGCCATCATGGAGAACTTCGATACCGCCCGTGTCCTCCTGAAGCGTAAGGCCGATGTGAACGCTAAGAATAACCGCGGCATGACTGCTCTGCATTACGCCACCGACTTCGATCACCGCGATCTCGTCGAGTGGTTGCTGGCCCATGGCGCCTCCATCTTGTAA

>gb|AE017196.1|:1-1267782_-_WD_0191_ORDERED_extraction Wolbachia endosymbiont of Drosophila melanogaster, complete genome

ATGATCAATAACCTGGACTCGATCATTTTGGGAATCAAGGATGGTGAGAAGGCTATCGAGGACCTGCGCGTCGTGCTGAAGAAGCGTAAGGAAGAGGTCATCACCACTCAGACCTTCAACTACGCCCTCCAGTGCCCCCAGACCACTAAGGAGGCCATCGTGCACGAAATCCTCCTGCACTTCATCCAGAACCCAGGTGAGCAGTCGCTGGAGCAGGTCATTCAATACCTGGACGGCGCTATCCAGTCCCGTATTTATGCCAAGAATAACCCCATCCGTAACCTCGATGAAGAGTTCCGCACCCATATCAACTTCAAGGACGAAGAGGGCAACACCCTGCTCCACCATGCCGTGATTGGCAACAAGACCGAAGAGATTACTACCCTGTTGGTTACTTACAGCGCCAACCCCTTGATCCAGAACGCCGACAACAAGGTCCCTTCCGACTTGGCCCAGGGTAAGACCAAGGAGGTCCTGATCAAGTCGATGAAGAAGCAGGCCAACACCAAGAAGGAGAGCGCCATGGTCGGTTCGCTGGTGCCCGGTGTTATTATCGGTGGCTTCTTGGGAGTCGTGCTCGGTGCCGGTGTGTGCGTCGCCGTGTCGCTGAGCGGAGGTATGATTCTGGGTGTGATGATCGCCAGCTCCCTGGTCGCCTCTATCGCCATCGGCCTGGCCATGTACTTCCTCTCCCAAGATTACGAACAGGCCAAGGCCATTGAAAAGACGATCTCTACCGTCTCGTCCGAGATCTCGGTGGATGACACTACCGTGGCCAACGGTAAGAAGGGCCCGCAGCTGACTTAA

>gb|AE017196.1|:1-1267782_-_WD_0206_ORDERED_extraction Wolbachia endosymbiont of Drosophila melanogaster, complete genome

ATGATCTCTAACATTATCCGCTCGATTGTGAAGTACCTGATGCGCAAGGTCATTAAGTACATCAGCATTATCGGTATCGCCTGCCTGGTGCTGTTGTTTTTCATCTCCAACGTGGAGACTCGCGTCAAGACCCAGGAAGAGCAGCTCTTCCTGGCTGTGGAGGATGGCAACGCCCAGGAAGTGAAGCTCCTGCTCAAGAACGGCGCTGACCCAAACTAA

>gb|AE017196.1|:1-1267782_-_WD_0285_ORDERED_extraction Wolbachia endosymbiont of Drosophila melanogaster, complete genome

ATGTGGATCTCGCAGGAAGAGCGTGATGACTTCAACGAGCTGTTCCAGGAGTTGCTGGCCGGCAAGCTGAGCAAGAAGCACATCAATCGCAAGAACGAGAACGGCGAGACCATGCTGCACCGTGCTGCCAAGATGTCCACCCGCAAGAAGGTCAACTGGTTGATCTGGGAAGGCGCCGACGTGGACGCCCGTGACAACGAAGGCTATACTCCCTTGCATTCGGCTGCCCTGGGCATGCGCCTGGAGAACGTTAAGGAGCTGATCGAGGCCCGCGCCGATATTAACGCCACCGAGGAAGATGGTAACACCGCCCTCCACCTGGCCTGCATGGTGGGTGGCAAGAAGATCGTGGAAGAGCTGATTAAGGCTGGTGCCGAGATTAACCTGGTCTCGATCAGCGGCTTCTCTCCGATGTATTACGCTTCGGACGAGGAAACCCGCGAGGTGCTGAAGAAGAAGGGTGGAAAGATCGTCAACAAGCAGCGTGAGCTGATGGAGAAGATCAGCAAGGTCTCCGAGAAGCAGGTCGTGAACGGCGGAAAGAAGCTGAACGATGTTGAAGAGCGTGCTGTCGACGTGTGCCGTCTGTTCGTCCGTAAGTAA

>gb|AE017196.1|:1-1267782_-_WD_0286_ORDERED_extraction Wolbachia endosymbiont of Drosophila melanogaster, complete genome

ATGGTGAAGTTCTCCAAGAAGGAGCGTGAAGAGTTCAACAAGAGCTGGAAGGAAGTCTTGGACAACTCCATTGAAAACATTAACAAGAAGGACACCAAGGGACGCACCATTCTGCACTACGCCGTCGGTATGCCTGATCCAAAGAAGGTTAAGCTCCTGATTAAGAAGGGCGCCGACGTGGATGCTGCCGACGCCGGCAAGTACCGCCCCCTGCATCTGGCCGTCATGGGCCAGCGTCTCGAGAACACCAAGGAGCTGATTAAGGCCGGCGTTGACGTCAACGCCGTCGAACGTTCCAGCAAGTTCGCTGCCCTGCACTTGGCCTGCATGGTGGCCGAGATCAAGATCGTGGAAGAGCTGGTCAAGGCCGGCGGTAACGTCGAACAGAAGGACAAGTTCGGAAAGACCCCCATGGACTACGTCCGTAATAACAAGGAGATTAAGGAGGTCCTGGAGAACGTGAAGATGGCTAACAAGCAGCGCGAGTTCATCGAGCGCATCCGCGTCGTGTCCGAATCGACCGCTGCCGGCGTGATCGTGGCTAAGGAAGAGGTCAAGGAGCTGGAGACCATGGACGAGAAGGTCCTGTAA

>gb|AE017196.1|:1-1267782_-_WD_0291_ORDERED_extraction Wolbachia endosymbiont of Drosophila melanogaster, complete genome

ATGCACACTTCTAAGCCCACCGCTGCCGAGAAGGATCTGAACAGCAAGCTGTTCTACGCCGTGGAGCAGAATAACCTGGACAAGGTCAAGGAGCTGATCCGTAACGGTGCCGATATCCACGCCCGTGAGATCTCTTCCAAGAAGACGATGCACATTGCTGTGAAGAAGGGCAACAAGAACATTGTCGAGTTTTTCCTGAACGAGGGCATCTCTGTCAATGACACCAATAACTCCGGTTGGACCCCCTTGCACTACGCCGCTTTCGGCGGTGAGCTGGAGATCGCCAAGCTCCTGGTGGCCAACGGCGCCAACGTCCGCGCTGAGAACGCCTACGGCCAGAAGCCCATCGATCTGATCCACTACGGTAAGGATGACGGATACAAGGGCATCATGGAGCTGTTGCTGAACAAGGGCGGTGGAAAGGTCAACGACATCGACAAGGAGGGTTGGACCTTGCTGCATTACGCTGCCTTCAACGGAAACCTGGAGACTGTGAAGTTCCTGATCGACAAGGGCGCCTCGATTCACACCAAGAATAACGGTCGTGAGACTCCCCTGGACCTGGCCCGTGAGGGAGGCTCTACCGAGGTTGTGAACATGCTGTCCAGCATCAACACCAAGGTCACCGACGTTTCGGTCTCGCAGCTGTCGATCCCCCGCAAGCGTACCGTGTAG

>gb|AE017196.1|:1-1267782_-_WD_0292_ORDERED,_RECEIVED_extraction Wolbachia endosymbiont of Drosophila melanogaster, complete genome

ATGCTGACCCGCGACATTTCCCGTCCCGAGCAGGTCTTCTCCGGCGCCGAGATCAGCGAGAAGAAGGAACTGGAAGAGAAGTCGAACACTCGCCAGGGTCAGAGCGAGATTGGAGGCTCTTTCGATGACCAGTATGAGCTGATCATTGAGTACTTGCTGCAGATCCCGCCCTCCAACGATCGTCTGGAGATCCTCCGTAAGCAGGGACATTCCCAGAGCGATATCGACGAGATGTTCTCCCAGGGCGATTCTTGGTACACTCAGTCCACCATCTCCTCGTTCCGCTCGTACAACTCCCCACCTTCCTTGATGGATTCGAAGTACAGCCTCTCCAAGCAGGTGTCCGTGTGGAGCGCTCTGGATCACAACCTGACCGAGAGCCAGAAGAAGTTGAACATCGAACTCCTGAACGTCCTGAAGTACCTGGAGTGGCACGATGAGGGCGCCTTGTTTGACTACGACAACGGCCAGGTGGATGAGCTGGAAGAGTTCCTGAAGAATAACCGTGGAAATCCCGATCTCAAGGCCGTCCTGAACGTGAAGCGCGGCGAGTCCGGATCGACCGTGCTGCACGCCATCGCCGGAGCCCACATCGGCGCTTCCTACCGTCAGGAGGACCGCACCATCAATCTGTTGCTGGAGGCCGGCGCCAGCCCCAACATCCAGGATGACAAGGGCGAGACGCCCCTGCACCGCGCCTCCGCCATGGGTTACGACAAGAACATTTACAGCTTGCTGCGCGGTAACGCCGATCCTAACATCTGCGACGGCCAGGGAAAGACCCCTCAACAGACTGCTGTCGACAATCACAACTACCACGTGGAGCGTTGCTTTTTCACCGATAACCAGAAGAAGCTGCGTAAGGAGCTCAATAACATCCTGAACCGCCACCGTTACAAGGAGGGCAACCTCAACTACATCGTCACCCTGACCCTCTTCCAGTTTTTCGAGGATCTGGAGCGCTTCCTGCACGAGCACAAGAACAATAAGGACCTGAAGGTGGTTCTGAACACCCGCAACATTGCCGGCAAGTCGGAGGTGCTGGAGCACGTGAAGAACGCCTTCTCCGGTTTCCAGGCTGCCGACGAGATTAAGAAGCTGCTCCTGGAGGCTGGAGCTAAGGAGTTCACCTACGAGCGCAAGAAGTGCCTGCCCAAGTCTGGTGTGCTGTGGGGAAATATCATTCCTGCCCAGGAAGAGAAGCTCTCCCGCTCCCTGGGCATTTTGTCCGAGATCCAGGACATCAACCAGTTGGAGAAGTTCGTCAAGATCGCCATCAAGTCGGGTGTCCGCCTGAACTACCGCTTCACGGTTCCCTCCCCGTTCGAGGGTTATTCCTTCACTGATTACGTGATCAAGCGTATTTCGGAGCTCGAGAAGTGCCCCAAGGTCGCCTCCGGAATTATCTGCCAGCTGGTGTCCAAGGGAGCCGTGTTCGGTTCCCCAGAGAGCATCGATGTCATCGATGAGCTGGGCCTGGAGTGCAAGGATCATAAGGCCAATATGATCAAGGCCTTCGAAGGATACATCAACGACGCCCACCGTTTCATCAAGGTGGCCAAGTCCGCCACGACCTCCAAGCTGAACGATATCGCCATTGGTAACACGACCCTGTACCTGGAGTACTCTGAAGAGTCGAAGATCGACATCGCCAAGATCACGGACGGCGCCCGCTCTCTCTGGCTGAATTACGAGAACGCCGGCTACGAGCGCAATATCGTTAAGATCGGTGAGTCCGAAGTCGAGATTATCACCCAGAACGGCAAGCGTCATTACACCGACTTGACTGCCAACAGCAATATTGCCCTGATCTTCTGCACTAGCTTCGGCGAGCTGGAGGTCCGCCTGTACTCCGACAAGCAGAACGAGAACCGCATTCGCGTCGAGGCCCGCGACCAAGGTATGTTGAAGAAGCTGAAGGACTGCGGCGAAGAGATTGGCAAGAACTGCTCCCTCGGCTACCACTCCGTGTACGACGCCATCGAGCGTGGATACTTCGAGAAGCCCGCTTCCTCGAGCCGCATCGTGCAGGAAGAGAAGTTGCAGAACGGCAAGTGGGCCGACCAGGTTCGCTACACCCGTAAGAACGGCGCTCAGATTCGCTAA

>gb|AE017196.1|:1-1267782_-_WD_0294_ORDERED_extraction Wolbachia endosymbiont of Drosophila melanogaster, complete genome

ATGAAGGATCTGGTGAACGTGCCCCGTAACCGCCAGGGATGGACGTCCCTGCACTACGCCGTCAAGAACGGTAACGTCGGCAAGATCAATGACCTGATCAAGGGAGGCAAGAACGTCGACGCCCAGGATGAACAGGGCTGGACCCCACTGCATCTGGCTGCCACCGGCTCGTACACCAAGGTTGTGAACGCCCAGATGTACGGTGATGACATCCACGCCCGCGAGACCGGTTCCGAAGAGCCCATCTACATTAAGGCCTGCAAGAACATTATCGAGTCCTTCCTGGACAAGCTGCTCAACATCAAGGTTGTGGGTGCCTTGATCAAGGGTAAGGCCGAGATCAACGCCAAGGACAACCAGGGTATGGCCCCGCTGCATTGGGCCGTCAAGGTGGGCCACATCAACGTGGTCAACGGCCTGATCAAGGGCAAGGCCGAGATTAACGCCAAGGATAACCAGGGCCGCACCCCTTTGCACTGGGCTGCCCTGATCGACCGCACCTCCGCTGTGAAGGCCTTGATCAAGGGCAAGGCCGAGATCAACGCCAAGGATAACCAGGAACGTACCCCACTCCATCTGTCCATCCAGATCGGTCGCACCGACGTGGTTAATACCCTGATCGACAAGAAGGCCGAAATCAACGCCAAGGATCGTCAAGGACGTACGCCCCTGCACTGGGCTGCCTCGAAGGGAGGCATCGAGGTCGTGAACGCCCTGATCGAAAAGGGCGCTGACGTGAACGCCGTGAACAAGTACGGAGACGCTCCCCTGCGTTTCGCTGCCCGCGACGGCCACATTGACATCGTCAAGGCTTTGATCCAGGGAGGTGCTAATGTCAACGCCCGTAATTCGGACGGAACCCCGTTGCACACCGCTTACGGTCACGAAGAGATCGTTAAGTTGCTGATCGAGAAGGGCGCCGACGTCAACGCTGTGAACAGCAACGGTGACACCCCTCTGCGTTTCGCCGATCGTAACGGCCACATCGACACGGTCAAGGCTCTCATCAACTACGTCACCAAGCTGGAGGCTGCCGATTTGTACGTCAGCCAGAAGAACCTGGAAGAGAAGAACCGTCTGATTGGTGATCTGCACGACCTCCACTCCTCTTACCCACAGCACCTCCAGAACTGCAAGAAGGAGGTCAAGAAGATCGAGAAGGAGAGCCAGGAGCTGCACAGCTTCCTGAAGAAGTCCGACATCAACGAACTGATCTCCGTGTGGGAGCGTAACGCCGATATTCAGAACCAGATCGATAACCATGATAACCTGAAGGAGCAGTACCCCGAGTACGCCCACATTCTCATCAACAAGGCCAACGAGGTCAAGAAGGAGATCTTCCTGCACAACCATCAGCCCCTGATCGACGCCTTGTCCGCTCACTATAAGTGTGATATCAAGACTATGACCTTCGCCGGAATCGAGAACTTTTTCAAGGTGGTCCACCGCGACGATTTCAAGGAGAAGCTGGGCAACGGAGGCATTACGCTGAAGAACTTCGTCGACCTGAAGAACGTGGAAAAGCGTGACGATGTGCCCAAGCTGGGCGTGATCGCTTTCAAGCACGGATGCCAGCTGTCCGAGCCCCGTGTCACCCGTGTTATCGATCAGGCCCTGAGCCTGTAG

>gb|AE017196.1|:1-1267782_-_WD_0385_ORDERED_extraction Wolbachia endosymbiont of Drosophila melanogaster, complete genome

ATGTCCAGCTCCCAAAGCCGCAATAACTTTTTCAAGGCCGTCGAGCGCGGCGATATCGACGCCGTGAACCGTCTGATTAGCGAGGGCGCCGACGTCAAGGTCGAGAACGATAAGGGCGAGACTCCCCTGCACATTGCTGCCGTCTGGGGCCACAAGGAAGTCGTGGAGGCCCTCCTGGACAAGGGCGCCAACGTCAACGCCGAGGACGAGGAAGGCAACACGCCCCTGGTCCTGACCACGGACGAGGAAATCAAGACTTTGCTGCAGTCCACGGCCAAGTTGCTGGAGGTCGCTAAGTCCGGTAACATCCAGGAGGTGAACTCGCTGATCTCCGAGGGCGCCAAGGTCAACGTCAAGGACCAGGATAACAAGACCCCACTGCACTGGGCTGCCGAGAAGGGCCACAAGGAGGTGGTCGAGGCCCTGTTGGACAAGGGCGCCAACGTGGACGCTGAGGATGAGAACGGCGATACTCCCCTGGATCTGGCCACGACCCAGGACATCCGCACCCTCCTGCAGAACACCGACGAGCTGTTGAAGGCTGCCGGTCGTGGTGACATCGACACCGTTAATGACTTGATCAACCAGGGTGCCAGCGTGAACGCCACCGATCAGGACGGCAAGACTCCACTCCATTGCGCTGCCAAGAACTCCCACGAAGAGGTTGTCGAGGCTCTGCTCGGAAAGGACGGAATCGACGTCAACCTGGCCGACAAGAACAAGGACACCCCTTTGCACTCGGTCCTGAAGAAGGGAAACATCGATATCAACGTTTTGAACGCCCTCCTGCGTAAGGAAGGAATTGATGTCAACCTGGCCGACAAGAACAAGGACACTCCCCTGCACTCCGTGCTCAAGAAGGATAACATTGATATTAACGTCTTGAACGCCCTCCTGGGAGCCAAGGAGATCAACGTCAACGCCCAGGATAAGGATGACCGTACCCCCTTGCATCTGGCTGCCAAGAAGGACAACATCGACATTAACGTTCTGAATGCCTTGCTGGGCGCTGAGGGAATTGATGTGAACATTAAGGATAAGCTGGCCGAGCAGACTCCCCTGCATTGGGCCGTCGTGAAGGGTCACAAGGAGGCCGTTGAGGCCTTGCTGGGCAAGGATGGCATCGACGTGAACATCGAGGATAAGCACGGTAACACCCCCTTCAAGTTGGCCACCGACGAGGGAATCAAGACCCTGTTGCAGCCCGCCGAGAAGTCCGATGACGGCAGCGCCGGTGGCTCCTCGACTGATTCCGAGGGAGGCCAGGAGGAAGAGAAGCGCGTCGGCGATGACACCGAACTGCAGTCGGACAACTCCAAGGAGGGAGAAAAGACCAGCACGACCAGCGCTGAGCAGGGCACTGACGTCCAGGATAACGATGTCGGCCCGGTGGCCTCTACCGAGCCCGCCCAGACCGAAGAGCAGCCCTCGTCCTTTTTCGGCTCCTTGTTCTCCATTTTGATGAAGCCCTTCTCGCTGATTGCCTCCTTTTTCGGCGGATTTTTCCATGGCTGCCTGGGCTTGACCAAGAAGAGCCTGACCCACAATCTGATGATGATTCTCCTGCACTTGGGCAGCATTAACCAGCTGAACAAGATCATGGTGATCATTATGACCCGCGCCATGTAG

>gb|AE017196.1|:1-1267782_-_WD_0434_ORDERED_extraction Wolbachia endosymbiont of Drosophila melanogaster, complete genome

ATGGATGGCCTGGTGGACATCGCCAAGCTCCTGTCCGACAACGAGGCCAACATCAACTGGCGCTACACCGGTCCTTTCGATAAGACGAAGCAGCGTGTCCAGGCCGGCAACCTGTTGCACCTGGCTGCCCGCATCCAGAACAAGGGTAAGTTCGTTGATATCTGCAAGAAGAACATCGCTTCGGTGACCGCCCACAACGAGTACGGCGACAACCCCTTCCATGAGGCTGCCCGTTCCGGCATCCTGCTCCCAGCCGTCCAGGAGATCGTGAACGATCTCGAGAAGGAGGCTAATAACAAGATCACGAAGGCCGAAGAGGCCGGTGACCGTAAGGAGGTCTCGCGCCTGAAGGAAGAGCTGAAGTGCAACAAGAAGTACATCAAGGATGCCCTGTGCTCCAAGGGTTACGCCTTCAACAAGAAGCGTGAGACCCCACTGTATTACCTCAACGCCGCTCAGCAAAAGGAGATCAAGCAGATCGCCGGTATCAAGGATAGCTTCATCTGCAACCAGAAGTTCCATCTGTGCCTGTACATCATTGGTGCCATCGCCTGCATCGCTGCCCTGTGCCTGTCCCTCTACTTCCTGTTCCTGGTGTCGCAGTCCTTCGCCTTGTCCAGCATGGTCGCCATCGCCAGCGGTGGCGTGACCTACCTGTCCGTTAAGGCCTGCTCTGAGATCCATGCCTTGCACAACGAATCCACCCTGGTGGAGAACGCCAACGTTCAGCTCGCCGGCGAGTCGCTGGGCGTGTAA

>gb|AE017196.1|:1-1267782_-_WD_0438_extraction Wolbachia endosymbiont of Drosophila melanogaster, complete genome

ATGGGCATCGACATCACCGCCCTGACTACCAACGCTGACAAGCTCGGAGAGTGCATCGATAAGAATAAGAAGCAGGAACAGGATCGTAAGGATGAGCAGCTGTACGGTATTATCGAGCGTAAGGTCAAGAAGGAAGAGCACAAGCGCGAGAAGCCACGTGGCCAGGACGGTACCCTGAAGAAGTGCAAGCGTCTGTTCGAAAAGGATGCTTCCCCAGAGGTGCTGACCAAGCTGGAAGAGTCCCTGCGCGAGCAAGAGAAGTATTACCGTTATTACGTCCAGTGCTTCCTGCCAGTTCTGGATAAGGCTATCGAACGCTCGAAGAAGATCTCCAACGAGACCAAGGAGCGTGTGAAGCAGGCCATCTCGGAGATCACCTACAATTCCAAGTACTGCAACCAGAACGATCGTCGCGATTCCGGCATCGAGTCCGATTACAGCTCCGATGCTGGTGACGATTACGAGAAGCATAGCATCTCCAATCCCAACACCGCCAACGAGGATCAGGAGGTCGAGAAGTCGAATGAGATCACCAACTCCAACGCCCTGTTGCTCAAGGCCATCAAGAACGGCAATAACCGCAAGTTCGAGAAGTACCTGAAGGATTGTACCGACATTTCCAGCATCAAGGATGAGAAGGGCAATAACATTCTGCACTTGATTGTGTCCCTGGAGAAGAAGCAGAAGCGTAAGTTCCTGGGAATCTTGATCAAGATGGTCACCAAGAAGGACCTGGCCCAGTTGATCAACAGCGAGAACCAGACTCCCCTGCAGGTGGCCCTGATCAATAAGATCACCAAGGAAAAGCATGAGAGCCACACCATCCAGGATAACGACAACACCCTGAAGTTCCTGCGCAAGTTGCTGGAGCACGGCGCCTCTCACGATCAGCTGGAGCTGCCAGAAGAGTCCCTGAAGAAGCTGTCTAAGGGACAGAATGAATATTACTGCAAGTTCCTGAAGAAGCTGGCCAAGAAGGCCGAGCTGAAGCAGGAGATTAAGATCAAGACCGAGGCCAAGGTGAAGGAGATCGTTGCCCACACCGTGAACACCCCCAACTCCAACTCCGACTACCCCCTGCACCTGGCCATCAAGAATAACGACAAGGAGCTGTTCAAGGAGCTCCTGCAGGAGGGTGCCAACATCTCTCTGGAGGGTGCTAACAAGAATAACGCTCTGCACCATATCGCCCTCCTGAAGGGCGAGTACAAGATCAAGTACCTGAAGCTGATCCTGGACTTCGAAAAGAAGGGTGTTATCTCGTCCGAGAAGCTGCACAAGGCTATCAACGCCCAAAACAAGGATGACAAGTACCCTGCTCAGGTGGCCCTGATCCGCCAGACCGAGAAGATTTCGTCCAAGTTGAAGGACTTCTCCCGTTGGGTCCGCAGCCGTCCCACCAAGCACTATTACACCGCCGAGTTCTGTGCCGAGTTGCTGCAGAACGGAGCCGAGAACAAGTCCGACCACATCACCATCCCCAAGGAGCACCGTAAGCAGAAGTATTACCAGACGACCAAGCGCATCAAGGACATCACCGGAGTTAGCCCCGATCCCGAGACCCAGGAAGAGCTGGAAGAGCACTTCTCGAAGAAGAACGCCCGCAAGAACATCAGCCTCCCGGTGAAGTGGATCCTGGGTGGACTGATCTTCGCCACCGTTTTCAGCGCCCTGGCCGGTAACATGATTGCTGCCGCTGCCCTGAGCGTCACGATTGCCGTGTGCGCCATGTGCTACCCAATCTACCTGAAGCTCGAGAACGCCCTCAACGCCCGTAGCTCCGATCAGTCCGAACATACGGAGAACCCCGCCACCAAGATGTCGGACGTGTCTATCCTGCGCCAGTTCATGAACAAGTTGGATGGCCTGACCAACAAGCCAAGCTCCCTGGCCTTGTAA

>gb|AE017196.1|:1-1267782_-_WD_0441_ORDERED_extraction Wolbachia endosymbiont of Drosophila melanogaster, complete genome

ATGCTGGGCATCTTCATGAAGAACATCCTGTACTTCATCTTGCTGGCCGTCGTGTTCGGTTCGCCGATCCTCTTCGCCGTGGAGCAGCTGGAAGAGAAGAAGATCGAGTCCGTGCACAAGAACGAGAACGTGTGCGTGCGCAAGCAAGATTCCACCAACCAGAAGGATGAGCTGAAGTCCAACGTCGACGCCAAGCAAACCCAGGAGGCCGAGTCCAAGCGTCTGGATGGAACTACCGACAAGGAAAAGTTGCAGCATAACGAGAACAAGGCCTTCGTGGTCGAGAAGACCATCTCCGAGGGCGAGCGTATTGACAAGGATTTGCCCAACGATCAGCTGGAGGGATCGACGGACAAGTTCGCTCAGAACCTGCCGAACGTGGCTAACAAGGAAGTCAATAAGGATTTGAAGCCCGAGCCCCTGCCGCTGTCCGCCGATTTGAATGAGAACACCGCTAACCCCCAGAAGAACCTGCAGGCCGACCAGAAGATCGATATCAAGGATAACGAGCTCTCGAAGAGCGATGCCTCCCAGTTGCTGGAGGGAAAGAAGGAGAAGGTGGAGAACCAGTCGGAGGAAAAGAAGGTTAAGGAGACCAATTCCAATTCCAAGGACCGCAACCGTGTGAAGCCCATCACCAAGAAGGACGAGGAAGAGCAGTCCGAGAAGAAGAGCCTGCAGAAGTGGACCAAGCTGAACCGCGAGCCCATTAAGGAGTGGGGACACAAGGATATCCAGTCCAAGAGCATTTACAAGCGTCAGTACGATTCCCTGAACGAGCACCTGCCAACTACCGTGTTCATCGATGACTACAGCAAGCAGTTTTTCTACTGCATCAAGAAGAATAACCTCACCTGCCTGCGCGGAGTGATCAGCAAGCTGGAAAAGATCGGTCTGACTATCCAAGAGATCCTGCGTTTCCGTAACAAGCTGGGTGATACCCCTCTGATCTATTCCGTGAAGCAAGGCGAGGTGGACATCGTCCGTTTCCTGCTCCTGCAAGGTGCTGATCTGCGCGTTGTGAACAATAACTTCCAGTCGCCCATTGACATCGCCATCGAGAAGAAGCAGATCAACATTATCAACGCCATCGCCGAGATGATGCCCCATCTCCTGGAGGACCGCAAGATCGACAACAAGGAGTCTAGCGCTATGTACGACTGGGCTGTGAAGACCAAGGAGATCCAGTGCGACAAGCAGGACGACTAA

>gb|AE017196.1|:1-1267782_-_WD_0498_ORDERED_extraction Wolbachia endosymbiont of Drosophila melanogaster, complete genome

ATGGCCCGCGAAGAGGATATCAACCAGCTGGTCGAGAACGGCTTCGACATCAATAGCAAGGATGCCTCTGGAATTACCCTGCTCCACAAGTTCACGAAGGAGGGAGACTTGGTCGGAGTCAAGTCCCTGTTGGAGCACGAAGCCGACTTCAACGTGGTCGATAATGAGAACCGCAACCCACTCCACTACGCCATCATGCACGGTCATAAGAAGGTGGCCAAGCTGTTCGTGAACCAGTTGACCATCAACTCCAAGGACAAGAACGGATTCACTCCCCTGCACCTGGCCGCTCTCCAGGATGACACCGAGCTGATCGATTTCCTGATCACCAAGGGAGCCAAGATCAACGAGAAGGATGCCAAGGAGGGTTACACGCCCCTCCACATTGCCTCGCTCTACGGTTCTAAGAAGTCCGTGCAGATCTTGATTGACAGCGGAGCCAACCTGGAGTGCGAGGATAATAACTTTCGCACTCCACTGTTCCTGACCATCTACCAGTGCACCGCCCATTACGACTCCCGTGCCGAGATTATCGAGTACCTGATTAAGAAGGGTGCCAACATCGAGGCCAAGGACGCTGAGAATAACACTACCCTGTTCCTGGCTGCCTACAACAATAAGATGCAGATTGTCAAGCTGATTGCCAAGAAGCAACAGGCTAGCAACGACAAGATCAAGCTCAAGGAGTTTTTCTGCACCAAGAATAACCACGGCTTCGATGCCCTGGATTGCGCTATCGAGCACAATAACCGCAAGATGGTCACCTTCCTGGTGTCGAAGGGCATCGAGGCTAACGACCAGGACTTCAACGGCAACGCCCGCCTGCACAAGGCCTCCCACAACGGTAACACCAAGACCGTTAAGCTGCTCTTGAAGCTCAAGGTGAACGTTAACGCCGTTACCAAGTGCAACCGTACTCCCCTCCTGCTCGCCGTTAAGAAGGGCCATACCCAGATCGTCAAGATGTTGCTCGAGGTCCGCGCTAATATGAACATCTGCGAGCAGTAA

>gb|AE017196.1|:1-1267782_-_WD_0514_ORDERED_extraction Wolbachia endosymbiont of Drosophila melanogaster, complete genome

ATGGAGACCGTGCCCGAGAACCAGGAAGAGATCATTATCGGTCTGGAGAACAAGGTCCGCGAGCTCAAGAAGGAGATTGAAGAGATCAAGAACAGCTCGAGCGCCTCGGATGAGTCCTACAAGGAGCAGATTTCCTCGCTGGAGGGACAGCTGATCGTGCTGCAACGCTCCTTCAACGACATGAAGCGCCAGTCTTCGCAGACCCTGTCCGAGAAGGCCAACGAGATCAAGCGCTTGTCCGCCATTGTCAAGAACCTGCGCAAGCAGGGTGAAGACCTGAACAAGAAGATCCAGGAGAATGAGCGCAAGTACCTGTACGTGGAGTGCACCAAGGATATTGAGGCCCGTAAGTTCAAGGATGCCGAAGAGAAGCTGAAGCAGATCAATAACGAGACCGAGACCTCCAACGTCGTGAAGCAGGTCTACAGCGGCCGTATCAATAACTTTCCATTGCTCCTGGAGTTTGGTAAGAGCATCGGCGATGCTAAGCTGAAGTTCTGGGTCTACGAGTCCTTGAGCGTTGAGATGGAGAACACCCAGCCAATCGATCTGCCGAAGACCGCCGAGCTGGCTAAGGCCTTGCGCATCGGTTGCATCGATCGCACCAACGTCCCCAACGACATCCGTGGAAAGGCCCGCGGCATCTTCGAGAAGTTGCGCAGCCTGGTGGTCGGCGCTAACGACCTCTGCCACGCCACGACCATGCATCACGCCGCTGAGATGGGTGATTTGGATGTGGTCCGCTTGCTCATCGACGGACGTGCCTACGTGGACTACCAGGATCAGCAATTGAAGACCCCTTTGTATTACGCTGCCGAGATGGGCAATCTGGATGTGGTCCGCCTGCTCATCGATAAGGGAGCCGACGTGAACCACCAAGATGAGTACCTGCAGACCCCACTGTACCTGGCCGCTGAAGAGGGAAAGCTGGACGTCGTGCGCTTGCTGATCGACAAGGGTGCCGACGTGAACCACCAGGACGAGTACTTGCAGACTCCCCTGCACTACGCCGCTGAGATGGGCAAGCTGGATGTCGTGCGCCTCCTGATCGACTCCGGTGCCTACGTCGACTCCAAGGGCAAGTACTTCGAGACGCCCCTGCAGCTGGCTGCCAAGGTGGGAAAGTTGGATGTGGTTCGCCTCCTGATTGACAAGGGCGCCGATGTCAACCACCGCGACCAACAGTCGCGCACGGCCTTGGAGTACGCCACCTCCAACTCCCGCTTCGATGTTGTCAAGTTCCTCAAGGAGAAGCAGGGTCTGCGTTCCCGTCGCGACGTCAAGGAACTGGGCAATGTTTCCAACTCGACGCTGGGCTCTTTGCAGGAGTCGGTGAAGCCCAGCTCCTACGTCTCGGATATCGTCCTGGAGAAGACCAAGGAGTCCAACTCGTCCTTCGTCCTCAAGTAA

>gb|AE017196.1|:1-1267782_-_WD_0550_ORDERED_extraction Wolbachia endosymbiont of Drosophila melanogaster, complete genome

TGGGCAGCGAGTTCGAAGAGATGCTGAAGGAGATCTTGCAGGAGATCAACGACCAGGGCTTCAACAAGGATAACATCATTGATAAGATTAAGGATAAGCTGCAGGAGAAGGACCCCCGTGTTTACAACGGATGGAAGGACAACAAGTTCGACATTGATCACCTGTTTTTGCCCGGCATCATGCTCCGTATGATCGCCAAGGACACCAAGCTGACCGGTTTTTTCATTGAGTACGAGCGCACCCTGTTGTACGTTGCTGCCGAGCACGGACACATCCAGATCGTGGAAAACTTGCTGGACAACGGCGCCAAGACCGGCATCAAGAACGGATACTGCAAGGAGGCTCCCCTGCACGTGGCTGCCAAGCACGGCCACATTCGCATCGTCGAGATCCTGTCGAAGAAGGAGGCCGACATCGATTTGAAGAACCGCTACGGCGAGACCCCACTGCACTACGCTGCCAAGTACGGACACACCCAGGTCCTGGAGAACTTGCTGGGCCGCTCCACGAACGTCAACGTGCAGTCTGAGGTGGGACGTACGCCCCTGCATGACGCTGCCAATAACGGTCACATCGAGGTGGTCAAGCATCTGATTAAGAAGGGTGCCGATGTGAACGTGCAGAGCAAGGTGGGACGCACCCCCTTGCACAATGCTGCCAAGCACGGCCACACCCAGGTCGTGGAGGTCCTGCTCAAGAAGGGAGCCGACGTGAACATCCAAGATCGTGGCGGTCGCACTCCCCTGCACTACGCCGTGCAACGTCGCTACCCGAAGCTGGCCAAGCTGTTGCTGAACGACGGTGCCGATCCAAGCTTCATCCACCGCAGCAAGGCCATTACCGCTGGAGTCGCCGTCGGAATTCTGGCTGCCATCGTGACGCCCCTGGCCCTGGTCTACGCTACTGCCCTGCCGGCTCTCGCCATTATCGGCATTACCGTCGCCAGCTGCACCGATTCCTGGTGGAACTTCTTGTGGGGATGCGTGTAA

>gb|AE017196.1|:1-1267782_-_WD_0566_ORDERED_extraction Wolbachia endosymbiont of Drosophila melanogaster, complete genome

ATGAAGAAGATCAAGTACAACGAGCGTGACAAGCTGCACTTCGTCTGGTTCATTCTCCTGATCGTTTGCGTCGTTATCACCTACTGTTACCAGAAGTCCAAGGCCACCGACAACTACAACAAGACCCTGCAGGTCGCCACTTCGAATTGCAACCTCGGAATCGTGAAGCTGCTCGTCAAGGACATGGCCCCCAACCTGTCGGGCACGACCCTGCACTGCGCTGCCCGCAAGGGCTGCTTGGATATTATCCGTTTCCTGATTGAAGAGGAAAAGGTGAACATCAACGCCCTGGACCGTAACGCCTTCAAGCGTATCGCCCTCCATCACGCTGCCGGCGAGGGACACCTGGAGGTGATCAAGTTTCTCCTGGAGAAGGGCGCCAACCCCAACATCCGTGATATCGACGGTAAGAACCCCCGCGACGTGGCTGTTCTGCGCTCTCGTCATAACAAGGACAAGCCATACGACGAGATTATCCACCTCCTGTACAACGCCGAGAAGGAGCATGAGTCCGAGCAGTAA

>gb|AE017196.1|:1-1267782_-_WD_0596_ORDERED_extraction Wolbachia endosymbiont of Drosophila melanogaster, complete genome

ATGAGCGCCAACCTGTCCCTCGAGCTGATTAAGTGCCTGATCAATCAGCCCGGCGTGGACGTCAACGTCCGCGGTCTGAACGGCAAGACTCCCCTGCATTACGCCGTGGAGATCAATGAGTTGTCGATGGTGGCTCTCCTGCTCAACCGCAAGAACATTAACCCACTCATCACCGACGATAACGGAAAGTCGGCCCTGGATTGCGCCCGTGAAGAGATTCTGCAGGCCCTCATCAATCACAAGTATGGTTTGGAGAAGGATTCCTTGCTGCACTTGGCCGCTATGCTGAACGAGGCTAACGCCGTCCGCTTCCTGTTGGACAAGGGCACCAACGTCAACGAGCAGAACGCTTTGCTCCACACCCCGCTGCACCTGGCTGCCGGCGCTGGCCACGAACAGATCGTTGAGATTCTGATCCGCGAGGGAAACGCCGATAAGGACGTTCTGGACGCTCGTAACCACGCTGCCATCCACTACGCCGTGAATAACAAGAAGCTGGGTGTTGTGAAGCTCCTGTCGAACCTGGGAGCCAATGTCAATGTTGTCGGCAGCGGACGTAACGCTATGAAGCTCTCGAGCCTCCACGTGGCCATCTCCAGCTCGAACTACGACGAGCGTGATCTGTGCCTGGATATTGTCCGCTGCCTGATCAACGTCCCTAACGCCGGAGTTAATCTGCAGGACTACGAGAATAAGACGCCCCTGCACTACGCCGAACGTCTGAAGACCATCGAGGTGCTCCTGACTCGCGAGGACATTGACCCGCTGATCAAGGATGACAACGGCAAGACTCCGTTCTGCTACGCTAAGGAGGCCAACCGCCTGGATATCGTGAAGATTCTGGTTAGCAACCGCTACGGCGCCGATAAGAACTCCCTGCTCCACCTGGCTGCCCGCAAGGGATACGAAGATCTGATTGATGGCATTCTGGGCGAGGGTGTGGAGATTGACGCCGTCGACGAGTCCGGCAAGACCGGCATCTACGTGGCCGTGAAGCACGGCCATTTCAACGTCGTGAAGCTGTTGCTGAAGCGCGGCGCCGATGCTACCGACGTGTTCCAGTACGCCATTATCACTAATAACGCCAAGCTGATCAAGTTGCTGTCCAAGGAGAAGGAGATTGTGTTGTTTGGTCGTCAGAAGAACTTCCCCACCTTCCACCTGCTCTCTAACAAGTACTTCGAAGAGCGCAAGATCGCCGACAAGAAGATCAAGAAGTATATCAACATCGTCTGCGTGTCTATCACTGTGTGCGCCATCGTCATTGTGGGCATTTACCCCAACATTATCGCTGCCGTCATGGTGGGTATTGTTGCCCTCATCGCTGCCATCGCCATGTCCAATCTGACCCAGAAGTACATCGAAGAGGCCCTGGAGAAGAAGATGTTCATCGAACTCGAATCCGAAAAGACCTCCGAGTGCTCTTCCATCCTGTCCGACGTTGAGGTTTCCTCGAACGACGGCGAAGAGCTGGCTATCTAA

>gb|AE017196.1|:1-1267782_-_WD_0633_ORDERED,_RECEIVED_extraction Wolbachia endosymbiont of Drosophila melanogaster, complete genome

ATGGACACGGGTTCGGTGAAGTACATCGCCGAGTCCATCTCCGAGCAGTCCAAGCACACCGATGAGCAGAAGCTGAAGTGCGATCTGAGCGCCGGACAGGAGTTCTCCGAGATCGGCTCCCCCAAGACCGAGGCTACTGGCCTGCCCGTTGACTCCTATTACGAGTTCATCAAGCGCGATCTGTTTTGGGCCGATCGTTCGCTCATTGACGATAAGAAGTACCTGGAGGACCTGATCGATGAGTGGTTCATCCACGCCCCCAACCTGGGACTCGAAGAGTCCCAGAAGAAGCTGAACCAGAAGTTGCTGAACTCCATTATCAAGGACTTTAACATGGGCAACTACGATTCCTTCTCGAACCTGAAGCAGTTCCTGGAGTCTAACGAGAAGAACAAGGACCTCAAGTACGTCCTGAACCTGAAGCGCGGACACTTGGGCACTACCATTTTGAATGTGTTCCTGGAGTACGACGAGATTATCCCTTCGCTCCTGAAGGCCGGCGCCGATTTGAACATGCAGGACAACAAGGGTAAGACCCTGTTGCACGACACCGCCATCTACAGCTCCGGCTACGAGGACCTGGGCTACCTCCTGGACGCCAAGGCCGACCCCAACATCCAGGACGAGAAGGGAAACACCCCTCTCCATTACTATGCTGCCAAGTGCAGCGATCAGAGCCGTAAGACCATGGATCTCCTGATCTCGAAGGGCGCTGACTTGTCCATCAAGAATAACGATGGCAAGACTCCCCTCCAGGTGGCCATCGATAACGACAATATTATCGGCTGCTTGCTGACCAACTCCCAGAAGAAGCTGCGCGAGCAGTTGGGCAAGATGCTCCTGGCCACTTCCTCGGATGAGGACTGGGATAACGCCTATGACCCCGAGGTGGAGAACCTGCGTAAGTTCCTGAACCAGTATGAGAACGACAACGACCTGAAGATCGTGCTCAACGTGAAGGACGATTCTAGCGTCCTCCTGGATAGCCCCTCTCGTCGCTTTCCCAACGTTAAGGCTCTGCTCCTGAAGGCTGGCGCTGCCGACTTCATTGGTAAGAAGCAGGACAACTACGAGAAGTGCGACTCCTTCCTGTCCGAGATCTACCAGATCAACTACCTGGCCAAGCGTAACGAGTTCCTCTCCAAGGTCGTGAAGGCCAAGAGCATGATCGAGCTCCAGGAGGTGGTCAACGAGATTATCGCCAGCGGAATGCGTTTGAACTTCGCCAAGGATAAGGATTATTACTTCGCCGATCACGTCCTCGAGAAGATCGCCCAGCTGGAGGGTTCCTACGGCATCGCTTCCGACATCGTGTGCACCCTGATTTCCCGTGGTGCCAAGCTGAAGCGCTCCGAGAGCTTGAAGGTCATCGACACCATCGAGTTGAAGTTCAAGGCCCACAAGGCCAACATGATCAGCGCCCACCTGGAATACGTTTCCAACACCGAAGAGTTTTTCCGTATTGCTAAGGCTGCCACTTCTGGTCAGCTCTACGACGGCAAGATTGACAATAACGTGTTCTACCTGGAGTACAGCGAGGATAGCATTATCGACGTGGCCCGCATTACCGATCGTACCCGCAACCTCGAGCTGATTCAGGAGTCCTACCGTCGCGATATTATCAAGATCGGAAAGTCCAAGATGGAGATTATCACCGAGAATGGCATCCGTTATTACACCGATCTGACTGAGGGATCGGATATCGTGCTGACCTTCTACACCTCGCTGGGCAACATCGATCTCCGCCTGTACCCAGATATTCAGGACAAGTCCAAGATTATCGTCGAAGTTAGCAACCGCGAAGAGATCCTGGAGAAGTTCAAGGGCCGCGAGGAAGAGCTGGGAAACGACTGCGCCCTCGGTGGCTACTCCGTGTACAACGCTATCGAGCAGGGCTACTTCGAGCGTTCCCGCAAGCTGATGCGCCCCGAGGTCATCAGCGAGAGCAACAATAAGTGGACCGAGCGCGAAGAGCTGCGTCGCGACTCCATGGAAGAGATTGCCCGTCGCCACAAGTTGCTGCAGGATCTCCGCAATATTGAGTCCAACATCGTTCAGAAGGAGAAGAACTTCGATATCAAGACCTATCTGATCGACATTTTCAAGACCCTGTCTCGTTTCTACGAAGAGAAGGGTGACATCTCGAAGACCGATCTGGCCAAGGCCGCTGAGAAGGAGTCCAAGAAGCTGGGCCTGGAGGGCAAGTACAACTGGTCGAAGATCTTCGGACTGGAGGAAGAGATTATCGAGAAGGTGGAGAAGCAGGGCGACAAGGAGCAGAAGAGCAATATCCCCGACGATTTCTACCTGGGCCACGCCATCAATAACGGTTCCTGCTTTTTCGACTCTTTCCGTCAATCGCTCGAGCAACAGAAGGGCATCAAGGTCACCGTGGAGCAGCTGCGCAACGAGTGCAAGCGCTTCGCTCAGGACAATCCCCCGGAGTGGTTCATCTCCAAGATCGGCAACGACTTCGACGAGGTGGAATCGGAGCTGGTTAATCGTGGCATCACCTGCAACCAGTACATTAACTCGATCGGAAAGAACGAGTTCTGGGGTCGTTCCGACATCGAGGGCCGTGTGCTCTGCGATAAGTACGGCGTTAAGCTGCACGTGGCCGAGTCCAACCCGCTGCACACTATTGACAAGCAACAGGACCCCTTCCTGCACCAGCTGATTGATTCTTCCCGCAGCAAGGCCGGTAAGATCGATTACTCGCACAACTCGGCCCTCCACATGGTTAACGGAGGCCACGACCACTTCCAGCCCTTGCTGTACCGTAACAAGACTCTGGCCAAGCAGACCCAGGAGCAGAAGGACTCTCTGTGCTACTCCAGCCTCCCCTCCTGCAGCATGGATGAGCTGAAGATTGAGAAGGCCAACATCCGCCACTGCCTCTAA

>gb|AE017196.1|:1-1267782_-_WD_0636_ORDERED_extraction Wolbachia endosymbiont of Drosophila melanogaster, complete genome

ATGAGCAAGAAGGAGAAGGAGGACGAGCTGAAGGAGCTGTTGGAGAACTCCGGCAAGAATATCAAGGGCACCAAGAAGGAGCTGCATTATGCCGTTGACGGTAAGACCGTCTCCTTGCTCGTCGAGAAGGGCGCCAACGTTAACGCTGCCGACGTGGAAGGCTACACCGCTCTGCACCTGGCCATTACGGAGAAGCGTTTGGAGACCGTGCGTGAGCTCATCAAGTCTGGTGGAAACGTGAACGCTGAAGAGTACGGTTCTAAGTGCACTCCCCTGCACCTGGCCTGCATGGTTGGAAAGGTCGAAATCGTCGAAGAGCTGGTGAAGGCCGGAGCCGAGATCGAGCAGGCCGACAAGTTCGGTATGACCGCTATGGATTATGCTAAGAACAGCAAGGAGGTGACCGAGGTGCTGAAGAAGGAGACCGACCGTATTGAGAAGCTCTTCGAAAAGTTGTAG

>gb|AE017196.1|:1-1267782_-_WD_0637_ORDERED_extraction Wolbachia endosymbiont of Drosophila melanogaster, complete genome

TGCACCATGCCGTGGAGAACTCCGACCACAAGACCGTCCGCCTCCTGATCGAGAAGGGCGCTGAGATCAACGCTCGTGATAAGAACGGTTACACCCCGCTGCACTGCGCTGTGTTTGCCAAGTCGCTGGAGAACGTGAAGGTTCTCCTGCGCTCGGGTGCCGAGATCAACGCCACCCAGTATGTGTCCGGCTGCACCCCGCTGCACTCCGCCTGCAAGATTGGAGGTGCCGGCGTTGAGATTATCAAGGAGCTGGTCAAGGCCGGTTCCGAGGTGAACCAGCTGAACAAGTACGGCTCCACCCCCATGTATTACATCTGGGAGTCTGAGAAGTATTACCTCTGCGATCGCGAAGAGTCCGAGAAGGCCTCCAAGTTCCTCCGTGCCAAGGGCGGAGTGACCAAGTCTCGTAAGCTGACCTGCTACGGCATCGAGGGACTGGTGGGAGAAATCGCCGATAAGCTGGATCGCTCCTACATGCCAGAGCTGAAGATCATGGAGATCGAAGAGATTCGTAAGCGTGATAAGTCCCTGATCAAGAAGGAGTGCCAGAACCTGGCTACGAAGATCATGTGCCAGGTTAACGAGATGATTGACGAGGTGGTCCGCATCAAGAAGGGCACCTAA

>gb|AE017196.1|:1-1267782_-_WD_0754_ORDERED_extraction Wolbachia endosymbiont of Drosophila melanogaster, complete genome

ATGCTGTACGGTGGCGATGGAATCAATACCAACGGATACCGTTACAACCAGACTACCTCCCGCCAGCTGGACGAGCCGACCCAGAAGCTGTTCAAGGCCATTGATAATGAGAACCCTGAGGCCTTCAAGCAGGCCCTGAAGGAAGGAGCTGACGTTAACGCCTTCGACAAGGAGGGCATGACTCCCCTGATGTCGATCGTCAACGTCTGCGCCGTGTCCGGTGACGGCCAGGCCACCCTGGAGAAGATGGCCAAGTTGCTCATTCAGAATCGTTCCATCAACATCAACGCCCAGTCCAAGCAATCGGTGAGCACGACCCGCACCCGTTACGACCCCAGCACTCAGTCGGAGATCAGCGAGTTCATCACGACCAGCAATATGCGTAAGGACACTGCCCTGCATATCGCCTGCCAGGTGGGCGCCAAGGATGTGGTCAAGATCTTGCTGACTCATCCCGATATCAAGACCGACATCAAGAACTACGAATACAAGAGCCCCGAGGACTGCATCGCCCGCGGCTTCGAACGCGTGATCAAGCTGGAGTTCAAGAAGGCCCAGAAGGCCAATGAACTCCTGGGTGCCTTGTCGTCCCGCAACATTTACCAAGCCAAGCGTCCCCTGAACCAGGAGTTCAACCCGAACTGCTGGAAGCGCTCCCGTAACGAAGAGATCGAGACTCCTCTGTCTCTGATTATCCAGAGCTGCCTGCAGGGTATTACCAGCGATAACAAGGAGGTGCTGACCAAGTTGCTGAAGCATAAGGAGCTGGACTTTTCCCAGATCAAGCCCATCCAGGCCATTGAGCAAAACTCCTGGGTGAAGCAGATTATCGAGCAGGCCATCACCGAGCGTCTCACCGCTACCATTAACAAGAAGGATCTGGATGACGTCAAGAAGCTGGTGGAGGACAACTGCTTCATGAGCCACGCCATCGTGACCGCTGCCCTCCGCGGTGTCAATAACCCCATTGAGTCGATCACCAACTACCTGAACGAGAAGTTCCCAGCCAACACCCTGCAGCCCCTGGCCTCCACCAACGACATCCCCGTGGGCTCCGAACAGGTCATCCAGGAGCTCAAGGGCGAGCTGGAGCGTACGAAGGCCCAGCTGATCGAGAAGGAGCGCGAACTGGACCGCGTCGTGCGCGAGCGCACCCGCGGCATCAACAAGATCTCCCAGCTGGAAGAGGACCTGCGTCAGGAGAAGTCCGCCCAGAAGACTAAGATCAACGACCTGAACTCCGAGGTTACCCGTCTGAACCGCATCGTGTACGGTCGCGCTTCCGATACCGTGGAGATCTAA

>gb|AE017196.1|:1-1267782_-_WD_0766_ORDERED_extraction Wolbachia endosymbiont of Drosophila melanogaster, complete genome

ATGAAGTACGACAAGTTTATGGAGATCCTGAAGAAGATCAACGATCTGTCGGACCTCTCCAAGGATAACATTGTGGAGAAGATTAAGGCTAAGCTGCAGGAAGAGGACCCGGACCTGTGCCAGAAGTGGGAGAAGTCTAAGCCCGACAACGACAGCGGCTCCGGCATCAACTACATCTTCACCATTAGCCGCGGTCAAAACAGCCAGGAGGTGAAGTTGCTCCACTTCGCCAGCTACTGGAACTGCGCTAACGTCGCCAAGGCCCTGATTGAGAACGGCGCTGACATTAACGCCGAGCACGATAACAAGATCACTCCCCTGCATCTGGCTGCCCATTACGGCCATAAGGAGATCGTGCAGGTCCTGAGCAAGGCTGAGGGCATCAACGTCGATGCCAAGGACTCCGATGGACTCACGCCCCTCCACCTGGCCACCGCTAACTCGCACAAGGACGTTGTGGAGACCCTGATCGCTAACAAGGTGAACGTGAACGCCGAGGATGACGATCGCTGCACTCCCCTGCACCTGGCTGCCGAGGCCAACCACATCGAGGTGGTCAAGATCCTGGTCGAGAAGGCCGACGTCAACATCAAGGACGCCGACCGCTGGACCCCGCTGCACGTTGCTGCCGCTAACGGCCACAAGGATGTGGTCGAGACGCTGATCGCCAACAAGGTCAACGTGAACGCCGAAGATGACGATCGCTGCACGCCCCTGCATCTGGCCGCTGAGGCCAACCACATCGAGGTGGTCAAGATCCTGGTCGAGAAGGCCGATGTGAACATCAAGGACGCCGACCGCTGGACCCCCTTGCACGTTGCCGCTGCCAACGGACACGAAGATGTCGTGAAGACCTTGATCGCCAAGGGCGCCAAGGTCAAGGCCAAGAACGGCGATCGTCACACTCCCCTGCACTTCGCTGCCCAGAACGGCCATGAGGGCATCGTGAAGGTGCTCCTGGAGGCCGGAGCCGACCCGAGCCTGAAGGACGTGGACGGCAAGACTCCCCGTGACCTGACCAAGGACCAGGGCATTATCCAACTGCTCGAAGAGGCCGAGAAGAAGCAGACCTTGAAGAACGAGAACAAGAAGACCCCGAAGGACCTGACGGAGAACAAGGATGTCATGCAGTTGCCCGAGAAGAAGGAAGAGAAGCAGATCGGCAAGAACGCCATTGTCAAGGAGAAGGAGCAGTCCGCTAAGAATGCCATCGTCAAGGGCGTCATCGTCTGCTTTGTGACCGCCGTCATTGTTGGTGTCGCTCTCGCCTTTGCTACCGCCTTGTCCGTTCCAGCCATTATCGGCCTGGCTGCCGGCAGCGCTCTGATCGTGGGCGCCGGACAGTACATTATGAGCAAGCCCAAGCCCGAGATGAAGGAGGTGAAGGAGCCTGTCCCTCGTGAGACCGAGAAGGCCCTGACGTAA

>WD_1213_Ecoli_optimized_GeneWiz

CACCATGGTGGTCTCCTTCTACATCGGTGGCATCATTATGGGCATTATCGACAACTTCCTGATCAAGTCCGTCAAGAATAACGATATCAAGGGCGTCAAGTTGACCCTGATCCTGTATGACGCCTTGAACAGCTTCCGTAAGTTTTTCTCCAAGACCATGTCCCTGAAGAACACCGGCTCCTTCGACATCAACAATAACCAGGCTTTGAACCAGGCCTTGGGACACGCCACTGACCATAAGATTATGGAGCTCCTGATCAACCGCGGTGCCGACATCCACTCGATTGATGAGAACGGTAAGAACGTCATTATCCGTGTCATCGAGTCCCTCGCTGAGTCTAAGAACCTGAAGCTCGATTCCGAGAAGAACCTGCCCGCCGAGATTATCCTGCACCCCACGGTGAGCTTCTTGCTGGGTAAGGGCGCCGAGTATTACTCCACCGATGACCTGAACGTCGAGGCCATTCAGCAAAAGCCACCCCGCACCTCCGAGTACGAGCCCATTACTATGTACGCCGAAGTCAACAATAACGTCATTCCCCCACAGAAGCCCTCCCGCACCTTCGAATACAAGCCCAAGTCCACCAACTATGACGCCCTGCCCTCCAAGGAGCCCATCTACGCTGAGGTGTACGACGCCAAGGTGAGCAACTCCCTGGGCGAGAACGGATACCTGTCCGTGTCGGAAGAGTCCATCTACGCTGAGATCTACGACTCGCATAAGAAGTCGACCGAGAACCTGTCGCTGGAGGATTCTGGCTACCTGTCGATCTCGGAAGAGCCCATCTACTGCACCATCGATGACCTGTCCGAGCAGAAGAAGCCCACGAGCTTGAAGCAGAAGGAGCCCGAGCAGTCGTCCGAACCAATCTACGCTCGCGTGGATCTGTCTAAGAAGCGCGCTGAGCGCAAGGCCAAGGACATCAAGGCCAACTCCGGCACCTGCCATGGCCTGGTCCAGGATAAGGTTAACGACTGGCAGGTGAAGCTCAACGAGCTGTCCGCCAACAAGTCGATCAGCCAGCAACAGACTAACGACCAGAAGATCAAGACCAAGGCCGGTTTCTCGGTCGAAGAGATCCGCCAGAAGTACGAACGCCGTGATTCTGGTTACGACAGCGGCTACGATTCCGACACGAAGGTCGAGCAACAGCTGGAGCTGGCTCCCAAGTCCGAAATTGGCTCGGTTTCGATTGAGCATGGCGTGATCGGTGGCTGCCGTTACAAGATCACCGACATCTCGTTG

>WD_RS04160_ORDERED_extraction

ATGAGCATCAAGGAGCAGGAGATCGATGCCATCTTCCAGGAGATCGAGCAGGAATCCAAGGAGTCGGACATCATTGAAGAGGTGAAGCAGAAGTTGCTGGCCTGCAACCAGGACACTTACAACCAGTGGAAGCGCCTGAATTTCCACATCGATCACCTGTTCGAGATCCGTCAGGAGATCACCCACGCCAGCCGTTATTACACTACCGCCCTCAACATCGCTGCCCACAAGGGCCTGGTGAAGACCGTGCGTCGCCTCCTGGAAAAGGGTGCCGAGGTCTCTACTCGCGATGGAGGCGGTCACACCGCCCTGCACTGCGCCGCTTCGAGCGGCAACACGGAAATTATCGAACTGCTCCTGGAGCAGGGCGCCCACATCCACAGCTGCTCCAAGCTGGGATCCACCCCTTTGCATTTCGCTGCCACCAATAACCATATCAATGCTGTGCGCTGCTTGCTGAACAAGGGTGCCAGCCCCCTGGCCCTGGATAATAACAATTCTATTCCTTCCATGTTCGCCACCGACAAGGAGGTTATCGCCGTGCTGGAGAAGGCCGAGGAAGAGAAGCATAAGCGCGAGATGGAGTGCAAGAAGAAGCGCCCCGCCAAGATGATCGCCGTCGGCGGTATCGCTACCGCTCTCATCGTGGCCGGAATCGGTTACATCGTGGAGTTGCCCATTCTGGTCACCATTGGTATCTCCGTGTCCATCGCCCTGGTGTCTGTGTGCGTGGCTTACGTCATGAGCAAGCCCAACACCAAGATGGAAGGCACCGAGAGCTCCGTCCAGGAGATCGGCCAGTCTATTTAA

**DeltaAnks434_754.fasta:** Nucleotide sequences for constructs lacking ankyrin repeat domains expressed in Drosophila.

>WD_0434_delta_ank_(optimized)_Dmel_Gibson_Tails Wolbachia endosymbiont of Drosophila melanogaster, complete genome

ACTTCAGGCGGCCGCGGCATGGATGGCCTGGTGGACATCGCCAAGCTCCTGTCCGACAACGAGGCCAACATCAACTGGCGCTACACCGGTCCTTTCGATAAGACGAAGCAGCGTGTCCAGGCCGGCAACCTGTTGCACCTGGCTGCCCGCATCCAGAACAAGGGTAAGTTCGTTGATATCTGCAAGAAGAACATCGCTTCGGTGACCGCCCACAACGAGATCACGAAGGCCGAAGAGGCCGGTGACCGTAAGGAGGTCTCGCGCCTGAAGGAAGAGCTGAAGTGCAACAAGAAGTACATCAAGGATGCCCTGTGCTCCAAGGGTTACGCCTTCAACAAGAATCATCTGTGCCTGTACATCATTGGTGCCATCGCCTGCATCGCTGCCCTGTGCCTGTCCCTCTACTTCCTGTTCCTGGTGTCGCAGTCCTTCGCCTTGTCCAGCATGGTCGCCATCGCCAGCGGTGGCGTGACCTACCTGTCCGTTAAGGCCTGCTCTGAGATCCATGCCTTGCACAACGAATCCACCCTGGTGGAGAACGCCAACGTTCAGCTCGCCGGCGAGTCGCTGGGCGTGAGGATCTTTGTGAAGGAA

>WD_0754-deltaAnks_(optimized)_Dmel_Gibson_tails Wolbachia endosymbiont of Drosophila melanogaster, complete genome

ACTTCAGGCGGCCGCGGCATGAATTACGAGTACAAGTCGCCAGAGGATTGCATCGCCCGTGGTTTCGAGCGTGTGATTAAGCTCGAGTTCAAGAAGGCTCAGAAGGCCAACGAGTTGCTCGGCGCTTTGTCCTCGCGCAACATCTACCAGGCCAAGCGCCCCCTGAACCAGGAGTTCAACCCAAACTGCTGGAAGCGCTCGCGTAATGAAGAGATTGAGACGCCCCTCTCCCTGATTATCCAGAGCTGCTTGCAGGGAATCACCAGCGATAACAAGGAGGTGTTGACTAAGTTGCTGAAGCATAAGGAGCTCGACTTCTCGCAGATCAAGCCAATCCAGGCTATCGAGCAGAACTCGTGGGTCAAGCAGATTATCGAGCAGGCCATTACCGAGCGTCTGACCGCCACCATCAACAAGAAGGACCTGGATGACGTCAAGAAGCTGGTTGAGGACAACTGTTTCATGTCGCATGCCATTGTGACCGCCGCTCTCCGCGGTGTGAATAACCCCATCGAGTCGATTACCAACTACCTGAACGAAAAGTTCCCCGCCAACACCCTCCAGCCCCTGGCTAGCACTAATGACATCCCAGTTGGTTCCGAGCAGGTGATCCAGGAGCTCAAGGGCGAGCTGGAGCGTACCAAGGCCCAGCTGATCGAGAAGGAGCGCGAGCTCGACCGCGTCGTGCGTGAGCGCACCCGCGGCATTAATAAGATCTCCCAGCTGGAAGAGGATTTGCGCCAGGAGAAGTCTGCCCAAAAGACCAAGATCAACGACCTCAACTCCGAGGTCACCCGCCTGAACCGTATCGTGTATGGCCGCGCCAGCGACACTGTCGAGATCAGGATCTTTGTGAAGGAA

**AnksOnly_434_754.fasta:** Nucleotide sequences for constructs containing only ankyrin repeat domains expressed in Saccharomyces.

>gb|AE017196.1|:1-1267782_-_WD_0754_ORDERED_PCR_Product Wolbachia endosymbiont of Drosophila melanogaster, complete genome

CACCATGCTGTACGGTGGCGATGGAATCAATACCAACGGATACCGTTACAACCAGACTACCTCCCGCCAGCTGGACGAGCCGACCCAGAAGCTGTTCAAGGCCATTGATAATGAGAACCCTGAGGCCTTCAAGCAGGCCCTGAAGGAAGGAGCTGACGTTAACGCCTTCGACAAGGAGGGCATGACTCCCCTGATGTCGATCGTCAACGTCTGCGCCGTGTCCGGTGACGGCCAGGCCACCCTGGAGAAGATGGCCAAGTTGCTCATTCAGAATCGTTCCATCAACATCAACGCCCAGTCCAAGCAATCGGTGAGCACGACCCGCACCCGTTACGACCCCAGCACTCAGTCGGAGATCAGCGAGTTCATCACGACCAGCAATATGCGTAAGGACACTGCCCTGCATATCGCCTGCCAGGTGGGCGCCAAGGATGTGGTCAAGATCTTGCTGACTCATCCCGATATCAAGACCGACATCAAGAACTACGAATACAAGAGCCCCGAGGACTGCATCGCCCGCGGCTTCGAACGCGTGATCAAGCTGGAGTTCAAGAAGGCCCAGAAGGCCAATGAACTCCTGGGTGCCTTGTCGTCCCGCAACATTTACCAAGCC

>WD_0434_WT_PCR_Product Wolbachia endosymbiont of Drosophila melanogaster, complete genome

CACCATGAACATCGCTTCGGTGACCGCCCACAACGAGTACGGCGACAACCCCTTCCATGAGGCTGCCCGTTCCGGCATCCTGCTCCCAGCCGTCCAGGAGATCGTGAACGATCTCGAGAAGGAGGCTAATAACAAGATCACGAAGGCCGAAGAGGCCGGTGACCGTAAGGAGGTCTCGCGCCTGAAGGAAGAGCTGAAGTGCAACAAGAAGTACATCAAGGATGCCCTGTGCTCCAAGGGTTACGCCTTCAACAAGAAGCGTGAGACCCCACTGTATTACCTCAACGCCGCTCAGCAAAAGGAGATCAAGCAGATCGCCGGTATCAAGGATAGCTTCATCTGCAACCAGAAGTTCCATCTGTGCCTGTACATCATTGGTGCCATCGCCTGCATCGCTGCCCTGTGCCTG

**Drosophila_Expression_Plasmids.fasta:** Nucleotide sequences for the pJFRC7-20XUAS-IVS-mCD8::GFP and pPMW-attB_addgene pUASp plasmid with attB for Drosophila transgenesis

>pJFRC7-20XUAS-IVS-mCD8::GFP

GACTCAGGTGGCACTTTTCGGGGAAATGTGCGCGGAACCCCTATTTGTTTATTTTTCTAAATACATTCAAATATGTATCCGCTCATGAGACAATAACCCTGATAAATGCTTCAATAATATTGAAAAAGGAAGAGTATGAGTATTCAACATTTCCGTGTCGCCCTTATTCCCTTTTTTGCGGCATTTTGCCTTCCTGTTTTTGCTCACCCAGAAACGCTGGTGAAAGTAAAAGATGCTGAAGATCAGTTGGGTGCACGAGTGGGTTACATCGAACTGGATCTCAACAGCGGTAAGATCCTTGAGAGTTTTCGCCCCGAAGAACGTTTTCCAATGATGAGCACTTTTAAAGTTCTGCTATGTGGCGCGGTATTATCCCGTATTGACGCCGGGCAAGAGCAACTCGGTCGCCGCATACACTATTCTCAGAATGACTTGGTTGAGTACTCACCAGTCACAGAAAAGCATCTTACGGATGGCATGACAGTAAGAGAATTATGCAGTGCTGCCATAACCATGAGTGATAACACTGCGGCCAACTTACTTCTGACAACGATCGGAGGACCGAAGGAGCTAACCGCTTTTTTGCACAACATGGGGGATCATGTAACTCGCCTTGATCGTTGGGAACCGGAGCTGAATGAAGCCATACCAAACGACGAGCGTGACACCACGATGCCTGTAGCAATGGCAACAACGTTGCGCAAACTATTAACTGGCGAACTACTTACTCTAGCTTCCCGGCAACAATTAATAGACTGGATGGAGGCGGATAAAGTTGCAGGACCACTTCTGCGCTCGGCCCTTCCGGCTGGCTGGTTTATTGCTGATAAATCTGGAGCCGGTGAGCGTGGGTCTCGCGGTATCATTGCAGCACTGGGGCCAGATGGTAAGCCCTCCCGTATCGTAGTTATCTACACGACGGGGAGTCAGGCAACTATGGATGAACGAAATAGACAGATCGCTGAGATAGGTGCCTCACTGATTAAGCATTGGTAACTGTCAGACCAAGTTTACTCATATATACTTTAGATTGATTTAAAACTTCATTTTTAATTTAAAAGGATCTAGGTGAAGATCCTTTTTGATAATCTCATGACCAAAATCCCTTAACGTGAGTTTTCGTTCCACTGAGCGTCAGACCCCGTAGAAAAGATCAAAGGATCTTCTTGAGATCCTTTTTTTCTGCGCGTAATCTGCTGCTTGCAAACAAAAAAACCACCGCTACCAGCGGTGGTTTGTTTGCCGGATCAAGAGCTACCAACTCTTTTTCCGAAGGTAACTGGCTTCAGCAGAGCGCAGATACCAAATACTGTTCTTCTAGTGTAGCCGTAGTTAGGCCACCACTTCAAGAACTCTGTAGCACCGCCTACATACCTCGCTCTGCTAATCCTGTTACCAGTGGCTGCTGCCAGTGGCGATAAGTCGTGTCTTACCGGGTTGGACTCAAGACGATAGTTACCGGATAAGGCGCAGCGGTCGGGCTGAACGGGGGGTTCGTGCACACAGCCCAGCTTGGAGCGAACGACCTACACCGAACTGAGATACCTACAGCGTGAGCTATGAGAAAGCGCCACGCTTCCCGAAGGGAGAAAGGCGGACAGGTATCCGGTAAGCGGCAGGGTCGGAACAGGAGAGCGCACGAGGGAGCTTCCAGGGGGAAACGCCTGGTATCTTTATAGTCCTGTCGGGTTTCGCCACCTCTGACTTGAGCGTCGATTTTTGTGATGCTCGTCAGGGGGGCGGAGCCTATGGAAAAACGCCAGCAACGCGGCCTTTTTACGGTTCCTGGCCTTTTGCTGGCCTTTTGCTCACATGTTACCGTCGACGATGTAGGTCACGGTCTCGAAGCCGCGGTGCGGGTGCCAGGGCGTGCCCTTGGGCTCCCCGGGCGCGTACTCCACCTCACCCATCTGGTCCATCATGATGAACGGGTCGAGGTGGCGGTAGTTGATCCCGGCGAACGCGCGGCGCACCGGGAAGCCCTCGCCCTCGAAACCGCTGGGCGCGGTGGTCACGGTGAGCACGGGACGTGCGACGGCGTCGGCGGGTGCGGATACGCGGGGCAGCGTCAGCGGGTTCTCGACGGTCACGGCGGGCATGTCGACAAGCCGAACATATGGGCGCGCCTAGTATGTATGTAAGTTAATAAAACCCATTTTTGCGGAAAGTAGATAAAAAAAACATTTTTTTTTTTTACTGCACTGGATATCATTGAACTTATCTGATCAGTTTTAAATTTACTTCGATCCAAGGGTATTTGATGTACCAGGTTCTTTCGATTACCTCTCACTCAAAATGACATTCCACTCAAAGTCAGCGCTGTTTGCCTCCTTCTCTGTCCACAGAAATATCGCCGTCTCTTTCGCCGCTGCGTCCGCTATCTCTTTCGCCACCGTTTGTAGCGTTACGTAGCGTCAATGTCCGCCTTCAGTTGCATTTTGTCAGCGGTTTCGTGACGAAGCTCCAAGCGGTTTACGCCATCAATTAAACACAAAGTGCTGTGCCAAAACTCCTCTCGCTTCTTATTTTTGTTTGTTTTTTGAGTGATTGGGGTGGTGATTGGTTTTGGGTGGGTAAGCAGGGGAAAGTGTGAAAAATCCCGGCAATGGGCCAAGAGGATCAGGAGCTATTAATTCGCGGAGGCAGCAAACACCCATCTGCCGAGCATCTGAACAATGTGAGTAGTACATGTGCATACATCTTAAGTTCACTTGATCTATAGGAACTGCGATTGCAACATCAAATTGTCTGCGGCGTGAGAACTGCGACCCACAAAAATCCCAAACCGCAATTGCACAAACAAATAGTGACACGAAACAGATTATTCTGGTAGCTGTTCTCGCTATATAAGACAATTTTTGAGATCATATCATGATCAAGACATCTAAAGGCATTCATTTTCGACTATATTCTTTTTTACAAAAAATATAACAACCAGATATTTTAAGCTGATCCTAGATGCACAAAAAATAAATAAAAGTATAAACCTACTTCGTAGGATACTTCGGGGTACTTTTTGTTCGGGGTTAGATGAGCATAACGCTTGTAGTTGATATTTGAGATCCCCTATCATTGCAGGGTGACAGCGGAGCGGCTTCGCAGAGCTGCATTAACCAGGGCTTCGGGCAGGCCAAAAACTACGGCACGCTCCGGCCACCCAGTCCGCCGGAGGACTCCGGTTCAGGGAGCGGCCAACTAGCCGAGAACCTCACCTATGCCTGGCACAATATGGACATCTTTGGGGCGGTCAATCAGCCGGGCTCCGGATGGCGGCAGCTGGTCAACCGGACACGCGGACTATTCTGCAACGAGCGACACATACCGGCGCCCAGGAAACATTTGCTCAAGAACGGTGAGTTTCTATTCGCAGTCGGCTGATCTGTGTGAAATCTTAATAAAGGGTCCAATTACCAATTTGAAACTCAGTTTGCGGCGTGGCCTATCCGGGCGAACTTTTGGCCGTGATGGGCAGTTCCGGTGCCGGAAAGACGACCCTGCTGAATGCCCTTGCCTTTCGATCGCCGCAGGGCATCCAAGTATCGCCATCCGGGATGCGACTGCTCAATGGCCAACCTGTGGACGCCAAGGAGATGCAGGCCAGGTGCGCCTATGTCCAGCAGGATGACCTCTTTATCGGCTCCCTAACGGCCAGGGAACACCTGATTTTCCAAGCCATGGTGCGGATGCCACGACATCTGACCTATCGGCAGCGAGTGGCCCGCGTGGATCAGGTGATCCAGGAGCTTTCGCTCAGCAAATGTCAGCACACGATCATCGGTGTGCCCGGCAGGGTGAAAGGTCTGTCCGGCGGAGAAAGGAAGCGTCTGGCATTCGCCTCCGAGGCTCTAACCGATCCGCCGCTTCTGATCTGCGATGAGCCCACCTCCGGACTGGACTCCTTTACCGCCCACAGCGTCGTCCAGGTGCTGAAGAAGCTGTCGCAGAAGGGCAAGACCGTCATCCTGACCATTCATCAGCCGTCTTCCGAGCTGTTTGAGCTCTTTGACAAGATCCTTCTGATGGCCGAGGGCAGGGTAGCTTTCTTGGGCACTCCCAGCGAAGCCGTCGACTTCTTTTCCTAGTGAGTTCGATGTGTTTATTAAGGGTATCTAGTATTACATAACATCTCAACTCCTATCCAGCGTGGGTGCCCAGTGTCCTACCAACTACAATCCGGCGGACTTTTACGTACAGGTGTTGGCCGTTGTGCCCGGACGGGAGATCGAGTCCCGTGATCGGATCGCCAAGATATGCGACAATTTTGCCATTAGCAAAGTAGCCCGGGATATGGAGCAGTTGTTGGCCACCAAAAATCTGGAGAAGCCACTGGAGCAGCCGGAGAATGGGTACACCTACAAGGCCACCTGGTTCATGCAGTTCCGGGCGGTCCTGTGGCGATCCTGGCTGTCGGTGCTCAAGGAACCACTCCTCGTAAAAGTGCGACTTATTCAGACAACGGTGAGTGGTTCCAGTGGAAACAAATGATATAACGCTTACAATTCTTGGAAACAAATTCGCTAGATTTTAGATAGAATTGCCTGATTCCACACCCTTCTTAGTTTTTTTCAATGAGATGTATAGTTTATAGTTTTGCAGAAGATAAATAAATTTCATTTAACTCGCGAATATTAATGAGATGCGAGTAACATTTTAATTTGCAGATGGTTGCCATCTTGATTGGCCTCATCTTTTTGGGCCAACAACTCACGCAAGTGGGTGTGATGAATATCAACGGAGCCATCTTCCTCTTCCTGACCAACATGACCTTTCAAAACGTCTTTGCCACGATAAATGTAAGTCATGTTTAGAATACATTTGCATTTCAATAATTTACTAACTTTCTAATGAATCGATTCGATTTAGGTGTTCACCTCAGAGCTGCCAGTTTTTATGAGGGAGGCCCGAAGTCGACTTTATCGCTGTGACACATACTTTCTGGGCAAAACGATTGCCGAATTGCCGCTTTTTCTCACAGTGCCACTGGTCTTCACGGCGATTGCCTATCCGATGATCGGACTGCGGGCCGGAGTGCTGCACTTCTTCAACTGCCTGGCGCTGGTCACTCTGGTGGCCAATGTGTCAACGTCCTTCGGATATCTAATATCCTGCGCCAGCTCCTCGACCTCGATGGCGCTGTCTGTGGGTCCGCCGGTTATCATACCATTCCTGCTCTTTGGCGGCTTCTTCTTGAACTCGGGCTCGGTGCCAGTATACCTCAAATGGTTGTCGTACCTCTCATGGTTCCGTTACGCCAACGAGGGTCTGCTGATTAACCAATGGGCGGACGTGGAGCCGGGCGAAATTAGCTGCACATCGTCGAACACCACGTGCCCCAGTTCGGGCAAGGTCATCCTGGAGACGCTTAACTTCTCCGCCGCCGATCTGCCGCTGGACTACGTGGGTCTGGCCATTCTCATCGTGAGCTTCCGGGTGCTCGCATATCTGGCTCTAAGACTTCGGGCCCGACGCAAGGAGTAGCCGACATATATCCGAAATAACTGCTTGTTTTTTTTTTTTACCATTATTACCATCGTGTTTACTGTTTATTGCCCCCTCAAAAAGCTAATGTAATTATATTTGTGCCAATAAAAACAAGATATGACCTATAGAATACAAGTATTTCCCCTTCGAACATCCCCACAAGTAGACTTTGGATTTGTCTTCTAACCAAAAGACTTACACACCTGCATACCTTACATCAAAAACTCGTTTATCGCTACATAAAACACCGGGATATATTTTTTATATACATACTTTTCAAATCGCGCGCCCTCTTCATAATTCACCTCCACCACACCACGTTTCGTAGTTGCTCTTTCGCTGTCTCCCACCCGCTCTCCGCAACACATTCACCTTTTGTTCGACGACCTTGGAGCGACTGTCGTTAGTTCCGCGCGATTCGGTTCGCTCAAATGGTTCCGAGTGGTTCATTTCGTCTCAATAGAAATTAGTAATAAATATTTGTATGTACAATTTATTTGCTCCAATATATTTGTATATATTTCCCTCACAGCTATATTTATTCTAATTTAATATTATGACTTTTTAAGGTAATTTTTTGTGACCTGTTCGGAGTGATTAGCGTTACAATTTGAACTGAAAGTGACATCCAGTGTTTGTTCCTTGTGTAGATGCATCTCAAAAAAATGGTGGGCATAATAGTGTTGTTTATATATATCAAAAATAACAACTATAATAATAAGAATACATTTAATTTAGAAAATGCTTGGATTTCACTGGAACTAGGGCGCGCCTCCGGAACATAATGGTGCAGGGCGCTGACTTCCGCGTTTCCAGACTTTACGAAACACGGAAACCGAAGACCATTCATGTTGTTGCTCAGGTCGCAGACGTTTTGCAGCAGCAGTCGCTTCACGTTCGCTCGCGTATCGGTGATTCATTCTGCTAACCAGTAAGGCAACCCCGCCAGCCTAGCCGGGTCCTCAACGACAGGAGCACGATCATGCGCACCCGTGGCCAGGGCCGCAAGCTTGCATGCCTGCAGGTCGGAGTACTGTCCTCCGAGCGGAGTACTGTCCTCCGAGCGGAGTACTGTCCTCCGAGCGGAGTACTGTCCTCCGAGCGGAGTACTGTCCTCCGAGCGGAGACTCTAGCCCTAGGGCATGCCTGCAGGTCGGAGTACTGTCCTCCGAGCGGAGTACTGTCCTCCGAGCGGAGTACTGTCCTCCGAGCGGAGTACTGTCCTCCGAGCGGAGTACTGTCCTCCGAGCGGAGACTCTAGCGCTAGCGCATGCCTGCAGGTCGGAGTACTGTCCTCCGAGCGGAGTACTGTCCTCCGAGCGGAGTACTGTCCTCCGAGCGGAGTACTGTCCTCCGAGCGGAGTACTGTCCTCCGAGCGGAGACTCTAGCACTAGTGCATGCCTGCAGGTCGGAGTACTGTCCTCCGAGCGGAGTACTGTCCTCCGAGCGGAGTACTGTCCTCCGAGCGGAGTACTGTCCTCCGAGCGGAGTACTGTCCTCCGAGCGGAGACTCTAGCGACGTCGAGCGCCGGAGTATAAATAGAGGCGCTTCGTCTACGGAGCGACAATTCAATTCAAACAAGCAAAGTGAACACGTCGCTAAGCGAAAGCTAAGCAAATAAACAAGCGCAGCTGAACAAGCTAAACAATCTGCAGTAAAGTGCAAGTTAAAGTGAATCAATTAAAAGTAACCAGCAACCAAGTAAATCAACTGCAACTACTGAAATCTGCCAAGAAGTAATTATTGAATACAAGAAGAGAACTCTGAATAGATCTAAAAGGTAGGTTCAACCACTGATGCCTAGGCACACCGAAACGACTAACCCTAATTCTTATCCTTTACTTCAGGCGGCCGCGGCTCGAGCAAAATGGCCTCACCGTTGACCCGCTTTCTGTCGCTGAACCTGCTGCTGCTGGGTGAGTCGATTATCCTGGGGAGTGGAGAAGCTAAGCCACAGGCACCCGAACTCCGAATCTTTCCAAAGAAAATGGACGCCGAACTTGGTCAGAAGGTGGACCTGGTATGTGAAGTGTTGGGGTCCGTTTCGCAAGGATGCTCTTGGCTCTTCCAGAACTCCAGCTCCAAACTCCCCCAGCCCACCTTCGTTGTCTATATGGCTTCATCCCACAACAAGATAACGTGGGACGAGAAGCTGAATTCGTCGAAACTGTTTTCTGCCATGAGGGACACGAATAATAAGTACGTTCTCACCCTGAACAAGTTCAGCAAGGAAAACGAAGGCTACTATTTCTGCTCAGTCATCAGCAACTCGGTGATGTACTTCAGTTCTGTCGTGCCAGTCCTTCAGAAAGTGAACTCTACTACTACCAAGCCAGTGCTGCGAACTCCCTCACCTGTGCACCCTACCGGGACATCTCAGCCCCAGAGACCAGAAGATTGTCGGCCCCGTGGCTCAGTGAAGGGGACCGGATTGGACTTCGCCTGTGATATTTACATCTGGGCACCCTTGGCCGGAATCTGCGTGGCCCTTCTGCTGTCCTTGATCATCACTCTCATCTGCTACCACAGCCGCGGATCCATGAGTAAAGGAGAAGAACTTTTCACTGGAGTTGTCCCAATTCTTGTTGAATTAGATGGTGATGTTAATGGGCACAAATTTTCTGTCAGTGGAGAGGGTGAAGGTGATGCAACATACGGAAAACTTACCCTTAAATTTATTTGCACTACTGGAAAACTACCTGTTCCATGGCCAACACTTGTCACTACTTTAACTTATGGTGTTCAATGCTTTTCAAGATACCCAGATCATATGAAACAGCATGACTTTTTCAAGAGTGCCATGCCCGAAGGTTATGTCCAGGAAAGAACTATATTTTTCAAAGATGACGGGAACTACAAGACACGTGCTGAAGTCAAGTTTGAAGGTGATACCCTTGTTAATAGAATCGAGTTAAAAGGTATTGATTTTAAAGAAGATGGAAACATTCTTGGACACAAATTGGAATACAACTATAACTCACACAATGTATACATCATGGCAGACAAACAAAAGAATGGAATCAAAGTTAACTTCAAAATTAGACACAACATTGAAGATGGAAGCGTTCAACTAGCAGACCATTATCAACAAAATACTCCAATTGGCGATGGCCCTGTCCTTTTACCAGACAACCATTACCTGTCCACACAATCTGCCCTTTCGAAAGATCCCAACGAAAAGAGAGACCACATGGTCCTTCTTGAGTTTGTAACAGCTGCTGGGATTACACATGGCATGGATGAACTATACAAATAATCTAGAGGATCTTTGTGAAGGAACCTTACTTCTGTGGTGTGACATAATTGGACAAACTACCTACAGAGATTTAAAGCTCTAAGGTAAATATAAAATTTTTAAGTGTATAATGTGTTAAACTACTGATTCTAATTGTTTGTGTATTTTAGATTCCAACCTATGGAACTGATGAATGGGAGCAGTGGTGGAATGCCTTTAATGAGGAAAACCTGTTTTGCTCAGAAGAAATGCCATCTAGTGATGATGAGGCTACTGCTGACTCTCAACATTCTACTCCTCCAAAAAAGAAGAGAAAGGTAGAAGACCCCAAGGACTTTCCTTCAGAATTGCTAAGTTTTTTGAGTCATGCTGTGTTTAGTAATAGAACTCTTGCTTGCTTTGCTATTTACACCACAAAGGAAAAAGCTGCACTGCTATACAAGAAAATTATGGAAAAATATTTGATGTATAGTGCCTTGACTAGAGATCATAATCAGCCATACCACATTTGTAGAGGTTTTACTTGCTTTAAAAAACCTCCCACACCTCCCCCTGAACCTGAAACATAAAATGAATGCAATTGTTGTTGTTAACTTGTTTATTGCAGCTTATAATGGTTACAAATAAAGCAATAGCATCACAAATTTCACAAATAAAGCATTTTTTTCACTGCATTCTAGTTGTGGTTTGTCCAAACTCATCAATGTATCTTATCATGTCTGGATCGATCTGGCCGGCCGTTTAAACGAATTCTTGAAGACGAAAGGGCCTCGTGATACGCCTATTTTTATAGGTTAATGTCATGATAATAATGGTTTCTTA

>pPMW-attB_addgene pUASp plasmid with attB for Drosophila transgenesis.

CATGATGAAATAACATAAGGTGGTCCCGTCGATAGCCGAAGCTTACCGAAGTATACACTTAAATTCAGTGCACGTTTGCTTGTTGAGAGGAAAGGTTGTGTGCGGACGAATTTTTTTTTGAAAACATTAACCCTTACGTGGAATAAAAAAAAATGAAATATTGCAAATTTTGCTGCAAAGCTGTGACTGGAGTAAAATTAATTCACGTGCCGAAGTGTGCTATTAAGAGAAAATTGTGGGAGCAGAGCCTTGGGTGCAGCCTTGGTGAAAACTCCCAAATTTGTGATACCCACTTTAATGATTCGCAGTGGAAGGCTGCACCTGCAAAAGGTCAGACATTTAAAAGGAGGCGACTCAACGCAGATGCCGTACCTAGTAAAGTGATAGAGCCTGAACCAGAAAAGATAAAAGAAGGCTATACCAGTGGGAGTACACAAACAGAGTAAGTTTGAATAGTAAAAAAAATCATTTATGTAAACAATAACGTGACTGTGCGTTAGGTCCTGTTCATTGTTTAATGAAAATAAGAGCTTGAGGGAAAAAATTCGTACTTTGGAGTACGAAATGCGTCGTTTAGAGCAGCAGCCGAATTAATTCTAGTTCCAGTGAAATCCAAGCATTTTCTAAATTAAATGTATTCTTATTATTATAGTTGTTATTTTTGATATATATAAACAACACTATTATGCCCACCATTTTTTTGAGATGCATCGTCGACGATGTAGGTCACGGTCTCGAAGCCGCGGTGCGGGTGCCAGGGCGTGCCCTTGGGCTCCCCGGGCGCGTACTCCACCTCACCCATCTGGTCCATCATGATGAACGGGTCGAGGTGGCGGTAGTTGATCCCGGCGAACGCGCGGCGCACCGGGAAGCCCTCGCCCTCGAAACCGCTGGGCGCGGTGGTCACGGTGAGCACGGGACGTGCGACGGCGTCGGCGGGTGCGGATACGCGGGGCAGCGTCAGCGGGTTCTCGACGGTCACGGCGGGCATGTCGACATGCATCTACACAAGGAACAAACACTGGATGTCACTTTCAGTTCAAATTGTAACGCTAATCACTCCGAACAGGTCACAAAAAATTACCTTAAAAAGTCATAATATTAAATTAGAATAAATATAGCTGTGAGGGAAATATATACAAATATATTGGAGCAAATAAATTGTACATACAAATATTTATTACTAATTTCTATTGAGACGAAATGAACCACTCGGAACCATTTGAGCGAACCGAATCGCGCGGAACTAACGACAGTCGCTCCAAGGTCGTCGAACAAAAGGTGAATGTGTTGCGGAGAGCGGGTGGGAGACAGCGAAAGAGCAACTACGAAACGTGGTGTGGTGGAGGTGAATTATGAAGAGGGCGCGCGATTTGAAAAGTATGTATATAAAAAATATATCCCGGTGTTTTATGTAGCGATAAACGAGTTTTTGATGTAAGGTATGCAGGTGTGTAAGTCTTTTGGTTAGAAGACAAATCCAAAGTCTACTTGTGGGGATGTTCGAAGGGGAAATACTTGTATTCTATAGGTCATATCTTGTTTTTATTGGCACAAATATAATTACATTAGCTTTTTGAGGGGGCAATAAACAGTAAACACGATGGTAATAATGGTAAAAAAAAAAACAAGCAGTTATTTCGGATATATGTCGGCTACTCCTTGCGTCGGGCCCGAAGTCTTAGAGCCAGATATGCGAGCACCCGGAAGCTCACGATGAGAATGGCCAGACCCACGTAGTCCAGCGGCAGATCGGCGGCGGAGAAGTTAAGCGTCTCCAGGATGACCTTGCCCGAACTGGGGCACGTGGTGTTCGACGATGTGCAGCTAATTTCGCCCGGCTCCACGTCCGCCCATTGGTTAATCAGCAGACCCTCGTTGGCGTAACGGAACCATGAGAGGTACGACAACCATTTGAGGTATACTGGCACCGAGCCCGAGTTCAAGAAGAAGCCGCCAAAGAGCAGGAATGGTATGATAACCGGCGGACCCACAGACAGCGCCATCGAGGTCGAGGAGCTGGCGCAGGATATTAGATATCCGAAGGACGTTGACACATTGGCCACCAGAGTGACCAGCGCCAGGCAGTTGAAGAAGTGCAGCACTCCGGCCCGCAGTCCGATCATCGGATAGGCAATCGCCGTGAAGACCAGTGGCACTGTGAGAAAAAGCGGTAATTCGGCAATCGTTTTGCCCAGAAAGTATGTGTCACAGCGATAAAGTCGACTTCGGGCCTCCCTCATAAAAACTGGCAGCTCTGAGGTGAACACCTAAATCGAATCGATTCATTAGAAAGTTAGTAAATTATTAATATGCAAATGTATTCTAAACAAGACTTACATTTATCGTGGCAAAGACGTTTTGAAAGGTCATGTTGGTCAGGAAGAGGAAGATGGCTCCGTTGATATTCATCACGCCCACTTGCGTGAGTTGTTGGCCCAAAAAGATGAGGCCAATCAAGATGGCAACCATCTGCAAATTAAAATGTTACTCGCATCTCATTAATATTCATATCTTCAACATGTTCGCGAGTTAAATGAAATTTATTTATTTTCTGCAAAACTATAAACTATACATCTCATTGAAAAAAACTAAGAAGGGTGTGGAATCAGGCAATTCTAACTAAAATCTAGCGAATTTGTTTCCAAGAATTGTAAGCGTTATATCATTTGTTTCCACTGGAACCACTCACCGTTGTCTGAATAAGTCGCACTTTTACGAGGAGTGGTTCCTTGAGCACCGACAGCCAGGATCGCCACAGGACCGCCCGGAACTGCATGAACCAGGTGGCCTTGTAGGTGTACCCATTCTCCGGCTGCTCCAGTGGCTTCTCCAAATTTTTGGTGGCCAACAACTGCTCCATATCCCGGGCTACTTTGCTAATAGCAAAATTGTCGCCATATCTTGGCGATCCGATCACGGGACTCGATCTCCCGTCCGGGCACAACGGCCAACACCTGTACGTAAAAGTCCGCCGGATTGTAGTTGGTAGGACACTGGGCACCCACGCTGGATAGGAGTTGAGATGTAATGTAATGCTAGATACCCTTAATAAACACATCGAACTCACTAGGAAAAGAAGTCGACGGCTTCGCTGGGAGTGCCCAAGAAAGCTACCCTGCCCTCGGCCATCAGAAGGATCTTGTCAAAGAGCTCAAACAGCTCGGAAGACGGCTGATGAATGGTCAGGATGACGGTCTTGCCCTTCTGCGACAGCTTCTTCAGCACCTGGACGACGCTGTGGGCGGTAAATGAGTCCAGTCCGGAGGTGGGCTCATCGCAGATCAGAAGCGGCGGATCGGTTAGTGCCTCGGAGGCGAATGCCAGACGCTTCCTTTCTCCGCCGGACAGACCTTTCACCCTGCCGGGCACACCGATGATCGTGTGCTGACATTTGCTGAGCGAAAGCTCCTGGATCACCTGATCCACGCGGGCCACTCGCTGCCGATAGGTCAGATGTCGTGGCATCCGCACCATGGCCTGGAAAATCAGGTGTTCCCTGGCCGTTAGGGAGCCGATAAAGAGGTCATCCTGCTGGACATAGGCGCACCTGGCCTGCATCTCCTTGGCGTCCACAGGTTGGCCATTGAGCAGTCGCATCCCGGATGGCGATACTTGGATGCCCTGCGGCGATCGAAAGGCAAGGGCATTCAGCAGGGTCGTCTTTCCGGCACCGGAACTGCCCATCACGGCCAAAAGTTCGCCCGGATAGGCCACGCCGCAAACTGAGTTTCAAATTGGTAATTGGACCCTTTATTAAGATTTCACACAGATCAGCCGACTGCGAATAGAAACTCACCGTTCTTGAGCAAATGTTTCCTGGGCGCCGGTATGTGTCGCTCGTTGCAGAATAGTCCGCGTGTCCGGTTGACCAGCTGCCGCCATCCGGAGCCCGGCTGATTGACCGCCCCAAAGATGTCCATATTGTGCCAGGCATAGGTGAGGTTCTCGGCTAGTTGGCCGCTCCCTGAACCGGAGTCCTCCGGCGGACTGGGTGGCAGGAGCGTGCCGTAGTTTTTGGCCTGCCCGAAGCCCTGGTTAATGCAGCTCTGCGAAGCGTCCGCTGTCACCCTGCAATGATAGGGGATCTCAAATATCAACTACAAGCGTTATGCTCATCTAACCCCGAACAAAACGAAGTATCCTACGAAGTAGGTTTATACTTTTATTTATTTTTTGTGCATCTAGGATCAGCTTAAAATATCTGGTTGTTATATTTTTTGTAAAAAAGAATGTAGTCGAAAATGAATGCCTTTAGATGTCTTGATCATGATATGATCTTAAAAATTGTCTTATATAGCGAGCACAGCTACCAGAATAATCTGTTTCGTGTCACTATTTGTTTGTGCGATTGCGGTTTGGGATTTTTGTGGGTCGCAGTTCTCACGCCGCAGACAATTTGATGTTGCAATCGCAGTTCCTATAGATCAAGTGAACTTAAGATGTATGCACATGTACTACTCACATTGTTCAGATGCTCGGCAGATGGGTGTTTGCTGCCTCCGCGAATTAATAGCTCCTGATCCTCTTGGCCCATTGCCGGGATTTTTCACACTTTCCCCTGCTTACCCACCCAAAACCAATCACCACCCCAATCACTCAAAAAACAAACAAAAATAAGAAGCGAGAGGAGTTTTGGCACAGCACTTTGTGTTTAATTGATGGCGTAAACCGCTTGGAGCTTCGTCACGAAACCGCTGACAAAATGCAACTGAAGGCGGACATTGACGCTACGTAACGCTACAAACGGTGGCGAAAGAGATAGCGGACGCAGCGGCGAAAGAGACGGCGATATTTCTGTGGACAGAGAAGGAGGCAAACAGCGCTGACTTTGAGTGGAATGTCATTTTGAGTGAGAGGTAATCGAAAGAACCTGGTACATCAAATACCCTTGGATCGAAGTAAATTTAAAACTGATCAGATAAGTTCAATGATATCCAGTGCAGTAAAAAAAAAAAAAAAAATATGTTTTTTTAAATCTACTTCTCCGCAAAAAATGGGTTTTATTAACTTACATACATACTAGAATTGGCCGCTCTAGCCCCCCTCGAATGTTCTCTCTCTTCTCTTCTCTCTCTCTTTCTCGAATGTTCTCTCTCTTCTCTTCTCTCTCTCTTTCTCGAGGTCATCAAGCTTAGGCCTCCAAGGCGGAGTACTGTCCTCCGGGCTGGCGGAGTACTGTCCTCCGGCAAGGCGGAGTACTGTCCTCCGGGCTGGCGGAGTACTGTCCTCCGGCAAGGCGGAGTACTGTCCTCCGGGCTGGCGGAGTACTGTCCTCCGGCAAGGCGGAGTACTGTCCTCCGGGCTGGCGGAGTACTGTCCTCCGGCAAGGCGGAGTACTGTCCTCCGGGCTGGCGGAGTACTGTCCTCCGGCAAGGCGGAGTACTGTCCTCCGGGCTGGCGGAGTACTGTCCTCCGGCAAGGCGGAGTACTGTCCTCCGGGCTGGCGGAGTACTGTCCTCCGGCAAGGGTCGAGTCGATAGCCGAAGCTTACCGAAGTATACACTTAAATTCAGTGCACGTTTGCTTGTTGAGAGGAAAGGTTGTGTGCGGACGAATTTTTTTTTGAAAACCGGTGATAGAGCCTGAACCAGAAAAGATAAAAGAAGGCTATACCAGTGGGAGTACACAAACAGAGTAAGTTTGAATAGTAAAAAAAATCATTTATGTAAACAATAACGTGACTGTGCGTTAGGTCCTGTTCATTGATCGAGGCCTGTCTAGAGAAGCTCCGCCACCATGGAGCAAAAGCTCATTTCTGAAGAGGACTTGAATGAAATGGAGCAAAAGCTCATTTCTGAAGAGGACTTGAATGAAATGGAGCAAAAGCTCATTTCTGAAGAGGACTTGAATGAAATGGAGCAAAAGCTCATTTCTGAAGAGGACTTGAATGAAATGGAGCAAAAGCTCATTTCTGAAGAGGACTTGAATGAAATGGAGAGCTTGGGCGACCTCACCATGGAGCAAAAGCTCATTTCTGAAGAGGACTTGAATCACCGGTATACAAGTTTGTACAAAAAAGCTGAACGAGAAACGTAAAATGATATAAATATCAATATATTAAATTAGATTTTGCATAAAAAACAGACTACATAATACTGTAAAACACAACATATCCAGTCACTATGGCGGCCGCATTAGGCACCCCAGGCTTTACACTTTATGCTTCCGGCTCGTATAATGTGTGGATTTTGAGTTAGGATCCGTCGAGATTTTCAGGAGCTAAGGAAGCTAAAATGGAGAAAAAAATCACTGGATATACCACCGTTGATATATCCCAATGGCATCGTAAAGAACATTTTGAGGCATTTCAGTCAGTTGCTCAATGTACCTATAACCAGACCGTTCAGCTGGATATTACGGCCTTTTTAAAGACCGTAAAGAAAAATAAGCACAAGTTTTATCCGGCCTTTATTCACATTCTTGCCCGCCTGATGAATGCTCATCCGGAATTCCGTATGGCAATGAAAGACGGTGAGCTGGTGATATGGGATAGTGTTCACCCTTGTTACACCGTTTTCCATGAGCAAACTGAAACGTTTTCATCGCTCTGGAGTGAATACCACGACGATTTCCGGCAGTTTCTACACATATATTCGCAAGATGTGGCGTGTTACGGTGAAAACCTGGCCTATTTCCCTAAAGGGTTTATTGAGAATATGTTTTTCGTCTCAGCCAATCCCTGGGTGAGTTTCACCAGTTTTGATTTAAACGTGGCCAATATGGACAACTTCTTCGCCCCCGTTTTCACCATGGGCAAATATTATACGCAAGGCGACAAGGTGCTGATGCCGCTGGCGATTCAGGTTCATCATGCCGTTTGTGATGGCTTCCATGTCGGCAGAATGCTTAATGAATTACAACAGTACTGCGATGAGTGGCAGGCGGGGCGTAATCTAGAGGATCCGGCTTACTAAAAGCCAGATAACAGTATGCGTATTTGCGCGCTGATTTTTGCGGTATAAGAATATATACTGATATGTATACCCGAAGTATGTCAAAAAGAGGTATGCTATGAAGCAGCGTATTACAGTGACAGTTGACAGCGACAGCTATCAGTTGCTCAAGGCATATATGATGTCAATATCTCCGGTCTGGTAAGCACAACCATGCAGAATGAAGCCCGTCGTCTGCGTGCCGAACGCTGGAAAGCGGAAAATCAGGAAGGGATGGCTGAGGTCGCCCGGTTTATTGAAATGAACGGCTCTTTTGCTGACGAGAACAGGGGCTGGTGAAATGCAGTTTAAGGTTTACACCTATAAAAGAGAGAGCCGTTATCGTCTGTTTGTGGATGTACAGAGTGATATTATTGACACGCCCGGGCGACGGATGGTGATCCCCCTGGCCAGTGCACGTCTGCTGTCAGATAAAGTCCCCCGTGAACTTTACCCGGTGGTGCATATCGGGGATGAAAGCTGGCGCATGATGACCACCGATATGGCCAGTGTGCCGGTCTCCGTTATCGGGGAAGAAGTGGCTGATCTCAGCCACCGCGAAAATGACATCAAAAACGCCATTAACCTGATGTTCTGGGGAATATAAATGTCAGGCTCCCTTATACACAGCCAGTCTGCAGGTCGACCATAGTGACTGGATATGTTGTGTTTTACAGTATTATGTAGTCTGTTTTTTATGCAAAATCTAATTTAATATATTGATATTTATATCATTTTACGTTTCTCGTTCAGCTTTCTTGTACAAAGTGGTGACGTAAGCTAGAGTCGACCTCGAACGTTAACGTTAACGTAACGTTAACTCGAGGAGCTTGATAACATTATACCTAAACCCATGGTCAAGAGTAAACATTTCTGCCTTTGAAGTTGAGAACACAATTAAGCATCCCCTGGTTAAACCTGACATTCATACTTGTTAATAGCGCCATAAACATAGCACCAATTTCGAAGAAATCAGTTAAAAGCAATTAGCAATTAGCAATTAGCAATAACTCTGCTGACTTCAAAACGAGAAGAGTTGCAAGTATTTGTAAGGCACAGTTTATAGACCACCGACGGCTCATTAGGGCTCGTCATGTAACTAAGCGCGGTGAAACCCAATTGAACATATAGTGGAATTATTATTATCAATGGGGAAGATTTAACCCTCAGGTAGCAAAGTAATTTAATTGCAAATAGAGAGTCCTAAGACTAAATAATATATTTAAAAATCTGGCCCTTTGACCTTGCTTGTCAGGTGCATTTGGGTTCAATCGTAAGTTGCTTCTATATAAACACTTTCCCCATCCCCGCAATAATGAAGAATACCGCAGAATAAAGAGAGATTTGCAACAAAAAATAAAGGCATTGCGAAAACTTTTTATGGGGGATCATTACACTCGGGCCTACGGTTACAATTCCCAGCCACTTAAGCGACAAGTTTGGCCAACAATCCATCTAATAGCTAATAGCGCAATCACTGGTAATCGCAAGAGTATATAGGCAATAGAACCCATGGATTTGACCAAAGGTAACCGAGACAATGGAGAAGCAAGAGGATTTCAAACTGAACACCCACAGTGCTGTGTACTACCACTGGCGCGTTTGGGAGCTCACTGGCCTGATGCGTCCTCCGGGCGTTTCAAGCCTGCTTTACGTGGTATACTCCATTACGGTCAACTTGGTGGTCACCGTGCTGTTTCCCTTGAGCTTGCTGGCCAGGCTGCTGTTCACCACCAACATGGCCGGATTGTGCGAGAACCTGACCATAACTATTACCGATATTGTGGCCAATTTGAAGTTTGCGAATGTGTACATGGTGAGGAAGCAGCTCCATGAGATTCGCTCTCTCCTAAGGCTCATGGACGCTAGAGCCCGGCTGGTGGGCGATCCCGAGGAGATTTCTGCCTTGAGGAAGGAAGTGAATATCGCACAGGGCACTTTCCGCACCTTTGCCAGTATTTTCGTATTTGGCACTACTTTGAGTTGCGTCCGCGTGGTCGTTCGCCCGGATCGAGAGCTCCTGTATCCGGCCTGGTTCGGCGTTGACTGGATGCACTCCACCAGAAACTATGTGCTCATCAATATCTACCAGCTCTTCGGCTTGATAGTGCAGGCTATACAGAACTGCGCTAGTGACTCCTATCCGCCTGCGTTTCTCTGCCTGCTCACGGGTCATATGCGTGCTTTGGAGCTGAGGGTGCGGCGGATTGGCTGCAGCCAAGCTTTGCGTACTCGCAAATTATTAAAAATAAAACTTTAAAAATAATTTCGTCTAATTAATATTATGAGTTAATTCAAACCCCACGGACATGCTAAGGGTTAATCAACAATCATATCGCTGTCTCACTCAGACTCAATACGACACTCAGAATACTATTCCTTTCACTCGCACTTATTGCAAGCATACGTTAAGTGGATGTCTCTTGCCGACGGGACCACCTTATGTTATTTCATCATGGTCTGGCCATTCTCATCGTGAGCTTCCGGGTGCTCGCATATCTGGCTCTAAGACTTCGGGCCCGACGCAAGGAGTAGCCGACATATATCCGAAATAACTGCTTGTTTTTTTTTTTACCATTATTACCATCGTGTTTACTGTTTATTGCCCCCTCAAAAAGCTAATGTAATTATATTTGTGCCAATAAAAACAAGATATGACCTATAGAATACAAGTATTTCCCCTTCGAACATCCCCACAAGTAGACTTTGGATTTGTCTTCTAACCAAAAGACTTACACACCTGCATACCTTACATCAAAAACTCGTTTATCGCTACATAAAACACCGGGATATATTTTTTATATACATACTTTTCAAATCGCGCGCCCTCTTCATAATTCACCTCCACCACACCACGTTTCGTAGTTGCTCTTTCGCTGTCTCCCACCCGCTCTCCGCAACACATTCACCTTTTGTTCGACGACCTTGGAGCGACTGTCGTTAGTTCCGCGCGATTCGGTGCGGTATTTCACACCGCATATGGTGCACTCTCAGTACAATCTGCTCTGATGCCGCATAGTTAAGCCAGCCCCGACACCCGCCAACACCCGCTGACGCGCCCTGACGGGCTTGTCTGCTCCCGGCATCCGCTTACAGACAAGCTGTGACCGTCTCCGGGAGCTGCATGTGTCAGAGGTTTTCACCGTCATCACCGAAACGCGCGAGACGAAAGGGCCTCGTGATACGCCTATTTTTATAGGTTAATGTCATGATAATAATGGTTTCTTAGACGTCAGGTGGCACTTTTCGGGGAAATGTGCGCGGAACCCCTATTTGTTTATTTTTCTAAATACATTCAAATATGTATCCGCTCATGAGACAATAACCCTGATAAATGCTTCAATAATATTGAAAAAGGAAGAGTATGAGTATTCAACATTTCCGTGTCGCCCTTATTCCCTTTTTTGCGGCATTTTGCCTTCCTGTTTTTGCTCACCCAGAAACGCTGGTGAAAGTAAAAGATGCTGAAGATCAGTTGGGTGCACGAGTGGGTTACATCGAACTGGATCTCAACAGCGGTAAGATCCTTGAGAGTTTTCGCCCCGAAGAACGTTTTCCAATGATGAGCACTTTTAAAGTTCTGCTATGTGGCGCGGTATTATCCCGTATTGACGCCGGGCAAGAGCAACTCGGTCGCCGCATACACTATTCTCAGAATGACTTGGTTGAGTACTCACCAGTCACAGAAAAGCATCTTACGGATGGCATGACAGTAAGAGAATTATGCAGTGCTGCCATAACCATGAGTGATAACACTGCGGCCAACTTACTTCTGACAACGATCGGAGGACCGAAGGAGCTAACCGCTTTTTTGCACAACATGGGGGATCATGTAACTCGCCTTGATCGTTGGGAACCGGAGCTGAATGAAGCCATACCAAACGACGAGCGTGACACCACGATGCCTGTAGCAATGGCAACAACGTTGCGCAAACTATTAACTGGCGAACTACTTACTCTAGCTTCCCGGCAACAATTAATAGACTGGATGGAGGCGGATAAAGTTGCAGGACCACTTCTGCGCTCGGCCCTTCCGGCTGGCTGGTTTATTGCTGATAAATCTGGAGCCGGTGAGCGTGGGTCTCGCGGTATCATTGCAGCACTGGGGCCAGATGGTAAGCCCTCCCGTATCGTAGTTATCTACACGACGGGGAGTCAGGCAACTATGGATGAACGAAATAGACAGATCGCTGAGATAGGTGCCTCACTGATTAAGCATTGGTAACTGTCAGACCAAGTTTACTCATATATACTTTAGATTGATTTAAAACTTCATTTTTAATTTAAAAGGATCTAGGTGAAGATCCTTTTTGATAATCTCATGACCAAAATCCCTTAACGTGAGTTTTCGTTCCACTGAGCGTCAGACCCCGTAGAAAAGATCAAAGGATCTTCTTGAGATCCTTTTTTTCTGCGCGTAATCTGCTGCTTGCAAACAAAAAAACCACCGCTACCAGCGGTGGTTTGTTTGCCGGATCAAGAGCTACCAACTCTTTTTCCGAAGGTAACTGGCTTCAGCAGAGCGCAGATACCAAATACTGTCCTTCTAGTGTAGCCGTAGTTAGGCCACCACTTCAAGAACTCTGTAGCACCGCCTACATACCTCGCTCTGCTAATCCTGTTACCAGTGGCTGCTGCCAGTGGCGATAAGTCGTGTCTTACCGGGTTGGACTCAAGACGATAGTTACCGGATAAGGCGCAGCGGTCGGGCTGAACGGGGGGTTCGTGCACACAGCCCAGCTTGGAGCGAACGACCTACACCGAACTGAGATACCTACAGCGTGAGCATTGAGAAAGCGCCACGCTTCCCGAAGGGAGAAAGGCGGACAGGTATCCGGTAAGCGGCAGGGTCGGAACAGGAGAGCGCACGAGGGAGCTTCCAGGGGGAAACGCCTGGTATCTTTATAGTCCTGTCGGGTTTCGCCACCTCTGACTTGAGCGTCGATTTTTGTGATGCTCGTCAGGGGGGCGGAGCCTATGGAAAAACGCCTTCTTCTTGAACTCGGGCTCGGTGCCAGTATACCTCAAATGGTTGTCGTACCTCTCATGGTTCCGTTACGCCAACGAGGGTCTGCTGATTAACCAATGGGCGGACGTGGAGCCGGGCGAAATTAGCTGCACATCGTCGAACACCACGTGCCCCAGTTCGGGCAAGGTCATCCTGGAGACGCTTAACTTCTCCGCCGCCGATCTGCCGCTGGACTACGTGGGTCTGGCC

**pPDEST-DB-cen.fasta**: Nucleotide sequence for yeast expression plasmid for Y2H assay.

>pDEST-DB-cen

AGCGGATAACAATTTCACACAGGAAACAGCTATGACCATGATTACGCCAAGCTCGGAATTAACCCTCACTAAAGGGAACAAAAGCTGGGTACCGGGCCCCCCCTCGAGATCCGGGATCGAAGAAATGATGGTAAATGAAATAGGAAATCAAGGAGCATGAAGGCAAAAGACAAATATAAGGGTCGAACGAAAAATAAAGTGAAAAGTGTTGATATGATGTATTTGGCTTTGCGGCGCCGAAAAAACGAGTTTACGCAATTGCACAATCATGCTGACTCTGTGGCGGACCCGCGCTCTTGCCGGCCCGGCGATAACGCTGGGCGTGAGGCTGTGCCCGGCGGAGTTTTTTGCGCCTGCATTTTCCAAGGTTTACCCTGCGCTAAGGGGCGAGATTGGAGAAGCAATAAGAATGCCGGTTGGGGTTGCGATGATGACGACCACGACAACTGGTGTCATTATTTAAGTTGCCGAAAGAACCTGAGTGCATTTGCAACATGAGTATACTAGAAGAATGAGCCAAGACTTGCGAGACGCGAGTTTGCCGGTGGTGCGAACAATAGAGCGACCATGACCTTGAAGGTGAGACGCGCATAACCGCTAGAGTACTTTGAAGAGGAAACAGCAATAGGGTTGCTACCAGTATAAATAGACAGGTACATACAACACTGGAAATGGTTGTCTGTTTGAGTACGCTTTCAATTCATTTGGGTGTGCACTTTATTATGTTACAATATGGAAGGGAACTTTACACTTCTCCTATGCACATATATTAATTAAAGTCCAATGCTAGTAGAGAAGGGGGGTAACACCCCTCCGCGCTCTTTTCCGATTTTTTTCTAAACCGTGGAATATTTCGGATATCCTTTTGTTGTTTCCGGGTGTACAATATGGACTTCCTCTTTTCTGGCAACCAAACCCATACATCGGGATTCCTATAATACCTTCGTTGGTCTCCCTAACATGTAGGTGGCGGAGGGGAGATATACAATAGAACAGATACCAGACAAGACATAATGGGCTAAACAAGACTACACCAATTACACTGCCTCATTGATGGTGGTACATAACGAACTAATACTGTAGCCCTAGACTTGATAGCCATCATCATATCGAAGTTTCACTACCCTTTTTCCATTTGCCATCTATTGAAGTAATAATAGGCGCATGCAACTTCTTTTCTTTTTTTTTCTTTTCTCTCTCCCCCGTTGTTGTCTCACCATATCCGCAATGACAAAAAAAATGATGGAAGACACTAAAGGAAAAAATTAACGACAAAGACAGCACCAACAGATGTCGTTGTTCCAGAGCTGATGAGGGGTATCTTCGAACACACGAAACTTTTTCCTTCCTTCATTCACGCACACTACTCTCTAATGAGCAACGGTATACGGCCTTCCTTCCAGTTACTTGAATTTGAAATAAAAAAAGTTTGCCGCTTTGCTATCAAGTATAAATAGACCTGCAATTATTAATCTTTTGTTTCCTCGTCATTGTTCTCGTTCCCTTTCTTCCTTGTTTCTTTTTCTGCACAATATTTCAAGCTATACCAAGCATACAATCAACTCCAAGCTTGAAGCAAGCCTCCTGAAAGATGAAGCTACTGTCTTCTATCGAACAAGCATGCGATATTTGCCGACTTAAAAAGCTCAAGTGCTCCAAAGAAAAACCGAAGTGCGCCAAGTGTCTGAAGAACAACTGGGAGTGTCGCTACTCTCCCAAAACCAAAAGGTCTCCGCTGACTAGGGCACATCTGACAGAAGTGGAATCAAGGCTAGAAAGACTGGAACAGCTATTTCTACTGATTTTTCCTCGAGAAGACCTTGACATGATTTTGAAAATGGATTCTTTACAGGATATAAAAGCATTGTTAACAGGATTATTTGTACAAGATAATGTGAATAAAGATGCCGTCACAGATAGATTGGCTTCAGTGGAGACTGATATGCCTCTAACATTGAGACAGCATAGAATAAGTGCGACATCATCATCGGAAGAGAGTAGTAACAAAGGTCAAAGACAGTTGACTGTATCGTCGAGGTCGAATCAAACAAGTTTGTACAAAAAAGCTGAACGAGAAACGTAAAATGATATAAATATCAATATATTAAATTAGATTTTGCATAAAAAACAGACTACATAATACTGTAAAACACAACATATCCAGTCACTATGGCGGCCGCGGGTGATGCTGCCAACTTAGCGGCCGCTAAGTTGGCAGCATCACCCGACGCACTTTGCGCCGAATAAATACCTGTGACGGAAGATCACTTCGCAGAATAAATAAATCCTGGTGTCCCTGTTGATACCGGGAAGCCCTGGGCCAACTTTTGGCGAAAATGAGACGTTGATCGGCACGTAAGAGGTTCCAACTTTCACCATAATGAAATAAGATCACTACCGGGCGTATTTTTTGAGTCATCGAGATTTTCAGGAGCTAAGGAAGCTAAAATGGAGAAAAAAATCACTGGATATACCACCGTTGATATATCCCAATGGCATCGTAAAGAACATTTTGAGGCATTTCAGTCAGTTGCTCAATGTACCTATAACCAGACCGTTCAGCTGGATATTACGGCCTTTTTAAAGACCGTAAAGAAAAATAAGCACAAGTTTTATCCGGCCTTTATTCACATTCTTGCCCGCCTGATGAATGCTCATCCGGAATTCCGTATGGCAATGAAAGACGGTGAGCTGGTGATATGGGATAGTGTTCACCCTTGTTACACCGTTTTCCATGAGCAAACTGAAACGTTTTCATCGCTCTGGAGTGAATACCACGACGATTTCCGGCAGTTTCTACACATATATTCGCAAGATGTGGCGTGTTACGGTGAAAACCTGGCCTATTTCCCTAAAGGGTTTATTGAGAATATGTTTTTCGTCTCAGCCAATCCCTGGGTGAGTTTCACCAGTTTTGATTTAAACGTGGCCAATATGGACAACTTCTTCGCCCCCGTTTTCACCATGGGCAAATATTATACGCAAGGCGACAAGGTGCTGATGCCGCTGGCGATTCAGGTTCATCATGCCGTTTGTGATGGCTTCCATGTCGGCAGAATGCTTAATGAATTACAACAGTACTGCGATGAGTGGCAGGCGGGGCGTAATCTAGAGGATCCGGCTTACTAAAAGCCAGATAACAGTATGCGTATTTGCGCGCTGATTTTTGCGGTATAAGAATATATACTGATATGTATACCCGAAGTATGTCAAAAAGAGGTATGCTATGAAGCAGCGTATTACAGTGACAGTTGACAGCGACAGCTATCAGTTGCTCAAGGCATATATGATGTCAATATCTCCGGTCTGGTAAGCACAACCATGCAGAATGAAGCCCGTCGTCTGCGTGCCGAACGCTGGAAAGCGGAAAATCAGGAAGGGATGGCTGAGGTCGCCCGGTTTATTGAAATGAACGGCTCTTTTGCTGACGAGAACAGGGGCTGGTGAAATGCAGTTTAAGGTTTACACCTATAAAAGAGAGAGCCGTTATCGTCTGTTTGTGGATGTACAGAGTGATATTATTGACACGCCCGGGCGACGGATGGTGATCCCCCTGGCCAGTGCACGTCTGCTGTCAGATAAAGTCCCCCGTGAACTTTACCCGGTGGTGCATATCGGGGATGAAAGCTGGCGCATGATGACCACCGATATGGCCAGTGTGCCGGTCTCCGTTATCGGGGAAGAAGTGGCTGATCTCAGCCACCGCGAAAATGACATCAAAAACGCCATTAACCTGATGTTCTGGGGAATATAAATGTCAGGCTCCCTTATACACAGCCAGTCTGCAGGTCGACCATAGTGACTGGATATGTTGTGTTTTACAGTATTATGTAGTCTGTTTTTTATGCAAAATCTAATTTAATATATTGATATTTATATCATTTTACGTTTCTCGTTCAGCTTTCTTGTACAAAGTGGTTTGATGGCCGCTAAGTAAGTAAGACGTCGAGCTCTAAGTAAGTAACGGCCGCCACCGCGGTGGAGCTTTGGACTTCTTCGCCAGAGGTTTGGTCAAGTCTCCAATCAAGGTTGTCGGCTTGTCTACCTTGCCAGAAATTTACGAAAAGATGGAAAAGGGTCAAATCGTTGGTAGATACGTTGTTGACACTTCTAAATAAGCGAATTTCTTATGATTTATGATTTTTATTATTAAATAAGTTATAAAAAAAATAAGTGTATACAAATTTTAAAGTGACTCTTAGGTTTTAAAACGAAAATTCTTATTCTTGAGTAACTCTTTCCTGTAGGTCAGGTTGCTTTCTCAGGTATAGCATGAGGTCGCTCTTATTGACCACACCTCTACCGGCATGCCGAGCAAATGCCTGCAAATCGCTCCCCATTTCACCCAATTGTAGATATGCTAACTCCAGCAATGAGTTGATGAATCTCGGTGTGTATTTTATGTCCTCAGAGGACAACACCTGTTGTAATCGTTCTTCCACACGGATCCCAATTCGCCCTATAGTGAGTCGTATTACAATTCACTGGCCGTCGTTTTACAACGTCGTGACTGGGAAAACCCTGGCGTTACCCAACTTAATCGCCTTGCAGCACATCCCCCTTTCGCCAGCTGGCGTAATAGCGAAGAGGCCCGCACCGATCGCCCTTCCCAACAGTTGCGCAGCCTGAATGGCGAATGGACGCGCCCTGTAGCGGCGCATTAAGCGCGGCGGGTGTGGTGGTTACGCGCAGCGTGACCGCTACACTTGCCAGCGCCCTAGCGCCCGCTCCTTTCGCTTTCTTCCCTTCCTTTCTCGCCACGTTCGCCGGCTTTCCCCGTCAAGCTCTAAATCGGGGGCTCCCTTTAGGGTTCCGATTTAGTGCTTTACGGCACCTCGACCCCAAAAAACTTGATTAGGGTGATGGTTCACGTAGTGGGCCATCGCCCTGATAGACGGTTTTTCGCCCTTTGACGTTGGAGTCCACGTTCTTTAATAGTGGACTCTTGTTCCAAACTGGAACAACACTCAACCCTATCTCGGTCTATTCTTTTGATTTATAAGGGATTTTGCCGATTTCGGCCTATTGGTTAAAAAATGAGCTGATTTAACAAAAATTTAACGCGAATTTTAACAAAATATTAACGCTTACAATTTCCTGATGCGGTATTTTCTCCTTACGCATCTGTGCGGTATTTCACACCGCATATCGACCGGTCGAGGAGAACTTCTAGTATATCTACATACCTAATATTATTGCCTTATTAAAAATGGAATCCCAACAATTACATCAAAATCCACATTCTCTTCAAAATCAATTGTCCTGTACTTCCTTGTTCATGTGTGTTCAAAAACGTTATATTTATAGGATAATTATACTCTATTTCTCAACAAGTAATTGGTTGTTTGGCCGAGCGGTCTAAGGCGCCTGATTCAAGAAATATCTTGACCGCAGTTAACTGTGGGAATACTCAGGTATCGTAAGATGCAAGAGTTCGAATCTCTTAGCAACCATTATTTTTTTCCTCAACATAACGAGAACACACAGGGGCGCTATCGCACAGAATCAAATTCGATGACTGGAAATTTTTTGTTAATTTCAGAGGTCGCCTGACGCATATACCTTTTTCAACTGAAAAATTGGGAGAAAAAGGAAAGGTGAGAGCGCCGGAACCGGCTTTTCATATAGAATAGAGAAGCGTTCATGACTAAATGCTTGCATCACAATACTTGAAGTTGACAATATTATTTAAGGACCTATTGTTTTTTCCAATAGGTGGTTAGCAATCGTCTTACTTTCTAACTTTTCTTACCTTTTACATTTCAGCAATATATATATATATATTTCAAGGATATACCATTCTAATGTCTGCCCCTAAGAAGATCGTCGTTTTGCCAGGTGACCACGTTGGTCAAGAAATCACAGCCGAAGCCATTAAGGTTCTTAAAGCTATTTCTGATGTTCGTTCCAATGTCAAGTTCGATTTCGAAAATCATTTAATTGGTGGTGCTGCTATCGATGCTACAGGTGTTCCACTTCCAGATGAGGCGCTGGAAGCCTCCAAGAAGGCTGATGCCGTTTTGTTAGGTGCTGTGGGTGGTCCTAAATGGGGTACCGGTAGTGTTAGACCTGAACAAGGTTTACTAAAAATCCGTAAAGAACTTCAATTGTACGCCAACTTAAGACCATGTAACTTTGCATCCGACTCTCTTTTAGACTTATCTCCAATCAAGCCACAATTTGCTAAAGGTACTGACTTCGTTGTTGTCAGAGAATTAGTGGGAGGTATTTACTTTGGTAAGAGAAAGGAAGACGATGGTGATGGTGTCGCTTGGGATAGTGAACAATACACCGTTCCAGAAGTGCAAAGAATCACAAGAATGGCCGCTTTCATGGCCCTACAACATGAGCCACCATTGCCTATTTGGTCCTTGGATAAAGCTAATGTTTTGGCCTCTTCAAGATTATGGAGAAAAACTGTGGAGGAAACCATCAAGAACGAATTCCCTACATTGAAGGTTCAACATCAATTGATTGATTCTGCCGCCATGATCCTAGTTAAGAACCCAACCCACCTAAATGGTATTATAATCACCAGCAACATGTTTGGTGATATCATCTCCGATGAAGCCTCCGTTATCCCAGGTTCCTTGGGTTTGTTGCCATCTGCGTCCTTGGCCTCTTTGCCAGACAAGAACACCGCATTTGGTTTGTACGAACCATGCCACGGTTCTGCTCCAGATTTGCCAAAGAATAAGGTCAACCCTATCGCCACTATCTTGTCTGCTGCAATGATGTTGAAATTGTCATTGAACTTGCCTGAAGAAGGTAAGGCCATTGAAGATGCAGTTAAAAAGGTTTTGGATGCAGGTATCAGAACTGGTGATTTAGGTGGTTCCAACAGTACCACCGAAGTCGGTGATGCTGTCGCCGAAGAAGTTAAGAAAATCCTTGCTTAAAAAGATTCTCTTTTTTTATGATATTTGTACATAAACTTTATAAATGAAATTCATAATAGAAACGACACGAAATTACAAAATGGAATATGTTCATAGGGTAGACGAAACTATATACGCAATCTACATACATTTATCAAGAAGGAGAAAAAGGAGGATGTAAAGGAATACAGGTAAGCAAATTGATACTAATGGCTCAACGTGATAAGGAAAAAGAATTGCACTTTAACATTAATATTGACAAGGAGGAGGGCACCACACAAAAAGTTAGGTGTAACAGAAAATCATGAAACTATGATTCCTAATTTATATATTGGAGGATTTTCTCTAAAAAAAAAAAAATACAACAAATAAAAAACACTCAATGACCTGACCATTTGATGGAGTTTAAGTCAATACCTTCTTGAACCATTTCCCATAATGGTGAAAGTTCCCTCAAGAATTTTACTCTGTCAGAAACGGCCTTAACGACGTAGTCGATATGGTGCACTCTCAGTACAATCTGCTCTGATGCCGCATAGTTAAGCCAGCCCCGACACCCGCCAACACCCGCTGACGCGCCCTGACGGGCTTGTCTGCTCCCGGCATCCGCTTACAGACAAGCTGTGACCGTCTCCGGGAGCTGCATGTGTCAGAGGTTTTCACCGTCATCACCGAAACGCGCGAGACGAAAGGGCCTCGTGATACGCCTATTTTTATAGGTTAATGTCATGATAATAATGGTTTCTTAGGACGGATCGCTTGCCTGTAACTTACACGCGCCTCGTATCTTTTAATGATGGAATAATTTGGGAATTTACTCTGTGTTTATTTATTTTTATGTTTTGTATTTGGATTTTAGAAAGTAAATAAAGAAGGTAGAAGAGTTACGGAATGAAGAAAAAAAAATAAACAAAGGTTTAAAAAATTTCAACAAAAAGCGTACTTTACATATATATTTATTAGACAAGAAAAGCAGATTAAATAGATATACATTCGATTAACGATAAGTAAAATGTAAAATCACAGGATTTTCGTGTGTGGTCTTCTACACAGACAAGATGAAACAATTCGGCATTAATACCTGAGAGCAGGAAGAGCAAGATAAAAGGTAGTATTTGTTGGCGATCCCCCTAGAGTCTTTTACATCTTCGGAAAACAAAAACTATTTTTTCTTTAATTTCTTTTTTTACTTTCTATTTTTAATTTATATATTTATATTAAAAAATTTAAATTATAATTATTTTTATAGCACGTGATGAAAAGGACCCAGGTGGCACTTTTCGGGGAAATGTGCGCGGAACCCCTATTTGTTTATTTTTCTAAATACATTCAAATATGTATCCGCTCATGAGACAATAACCCTGATAAATGCTTCAATAATATTGAAAAAGGAAGAGTATGAGTATTCAACATTTCCGTGTCGCCCTTATTCCCTTTTTTGCGGCATTTTGCCTTCCTGTTTTTGCTCACCCAGAAACGCTGGTGAAAGTAAAAGATGCTGAAGATCAGTTGGGTGCACGAGTGGGTTACATCGAACTGGATCTCAACAGCGGTAAGATCCTTGAGAGTTTTCGCCCCGAAGAACGTTTTCCAATGATGAGCACTTTTAAAGTTCTGCTATGTGGCGCGGTATTATCCCGTATTGACGCCGGGCAAGAGCAACTCGGTCGCCGCATACACTATTCTCAGAATGACTTGGTTGAGTACTCACCAGTCACAGAAAAGCATCTTACGGATGGCATGACAGTAAGAGAATTATGCAGTGCTGCCATAACCATGAGTGATAACACTGCGGCCAACTTACTTCTGACAACGATCGGAGGACCGAAGGAGCTAACCGCTTTTTTTCACAACATGGGGGATCATGTAACTCGCCTTGATCGTTGGGAACCGGAGCTGAATGAAGCCATACCAAACGACGAGCGTGACACCACGATGCCTGTAGCAATGGCAACAACGTTGCGCAAACTATTAACTGGCGAACTACTTACTCTAGCTTCCCGGCAACAATTAATAGACTGGATGGAGGCGGATAAAGTTGCAGGACCACTTCTGCGCTCGGCCCTTCCGGCTGGCTGGTTTATTGCTGATAAATCTGGAGCCGGTGAGCGTGGGTCTCGCGGTATCATTGCAGCACTGGGGCCAGATGGTAAGCCCTCCCGTATCGTAGTTATCTACACGACGGGCAGTCAGGCAACTATGGATGAACGAAATAGACAGATCGCTGAGATAGGTGCCTCACTGATTAAGCATTGGTAACTGTCAGACCAAGTTTACTCATATATACTTTAGATTGATTTAAAACTTCATTTTTAATTTAAAAGGATCTAGGTGAAGATCCTTTTTGATAATCTCATGACCAAAATCCCTTAACGTGAGTTTTCGTTCCACTGAGCGTCAGACCCCGTAGAAAAGATCAAAGGATCTTCTTGAGATCCTTTTTTTCTGCGCGTAATCTGCTGCTTGCAAACAAAAAAACCACCGCTACCAGCGGTGGTTTGTTTGCCGGATCAAGAGCTACCAACTCTTTTTCCGAAGGTAACTGGCTTCAGCAGAGCGCAGATACCAAATACTGTTCTTCTAGTGTAGCCGTAGTTAGGCCACCACTTCAAGAACTCTGTAGCACCGCCTACATACCTCGCTCTGCTAATCCTGTTACCAGTGGCTGCTGCCAGTGGCGATAAGTCGTGTCTTACCGGGTTGGACTCAAGACGATAGTTACCGGATAAGGCGCAGCGGTCGGGCTGAACGGGGGGTTCGTGCACACAGCCCAGCTTGGAGCGAACGACCTACACCGAACTGAGATACCTACAGCGTGAGCTATGAGAAAGCGCCACGCTTCCCGAAGGGAGAAAGGCGGACAGGTATCCGGTAAGCGGCAGGGTCGGAACAGGAGAGCGCACGAGGGAGCTTCCAGGGGGAAACGCCTGGTATCTTTATAGTCCTGTCGGGTTTCGCCACCTCTGACTTGAGCGTCGATTTTTGTGATGCTCGTCAGGGGGGCGGAGCCTATGGAAAAACGCCAGCAACGCGGCCTTTTTACGGTTCCTGGCCTTTTGCTGGCCTTTTGCTCACATGTTCTTTCCTGCGTTATCCCCTGATTCTGTGGATAACCGTATTACCGCCTTTGAGTGAGCTGATACCGCTCGCCGCAGCCGAACGACCGAGCGCAGCGAGTCAGTGAGCGAGGAAGCGGAAGAGCGCCCAATACGCAAACCGCCTCTCCCCGCGCGTTGGCCGATTCATTAATGCAGCTGGCACGACAGGTTTCCCGACTGGAAAGCGGGCAGTGAGCGCAACGCAATTAATGTGAGTTAGCTCACTCATTAGGCACCCCAGGCTTTACACTTTATGCTTCCGGCTCGTATGTTGTGTGGAATTGTG
